# Evaluation of stress distributions of calcium silicate-based root canal sealer in bulk or with main core material: A finite element analysis study

**DOI:** 10.1371/journal.pone.0299552

**Published:** 2024-03-14

**Authors:** Ahlam Smran, Mariam Abdullah, Norasmatul Akma Ahmad, Faycal ben Yahia, Ahmed Mahmoud Fouda, Sami A. Alturaiki, Nassr AL-Maflehi, Abdulaziz Samran

**Affiliations:** 1 Department of Restorative Dentistry, Faculty of Dentistry, University of Malaya, Kuala Lumpur, Malaysia; 2 Department of Restorative and Prosthetic Dental Sciences, College of Dentistry, Dar Al-Uloom University, Riyadh, Saudi Arabia; 3 Department of Mechanical Engineering, College of Engineering, King Saud University, Riyadh, Saudi Arabia; 4 Department of Oral Technology, University Hospital Bonn, Bonn University, Bonn, Germany; 5 Department of Fixed Prosthodontics, Suez Canal University, Ismailia, Egypt; 6 Department of Endodontics, King Fahad Medical City, Riyadh, Saudi Arabia; 7 Periodontics and Community Dentistry, College of Dentistry, King Saud University, Riyadh, Saudi Arabia; 8 Department of Prosthodontics, School of Dentistry, Ibb University, Ibb, Yemen; University of Puthisastra, CAMBODIA

## Abstract

This research aimed to assess the stress distribution in lower premolars that were obturated with BioRoot RCS or AH Plus, with or without gutta percha (GP), and subjected to vertical and oblique forces. One 3D geometric model of a mandibular second premolar was created using SolidWorks software. Eight different scenarios representing different root canal filling techniques, single cone technique with GP and bulk technique with sealer only with occlusal load directions were simulated as follows: Model 1 (BioRoot RCS sealer and GP under vertical load [VL]), Model 2 (BioRoot RCS sealer and GP under oblique load [OL]), Model 3 (AH Plus sealer with GP under VL), Model 4 (AH Plus sealer with GP under OL), Model 5 (BioRoot RCS sealer in bulk under VL), Model 6 (BioRoot RCS in bulk under OL), Model 7 (AH Plus sealer in bulk under VL), and Model 8 (AH Plus sealer in bulk under OL). A static load of 200 N was applied at three occlusal contact points, with a 45° angle from lingual to buccal. The von Mises stresses in root dentin were higher in cases where AH Plus was used compared to BioRoot RCS. Furthermore, shifting the load to an oblique direction resulted in increased stress levels. Replacing GP with sealer material had no effect on the dentin maximum von Mises stress in BioRoot RCS cases. Presence of a core material resulted in lower stress in dentin for AH Plus cases, however, it did not affect the stress levels in dentin for cases filled with BioRoot RCS. Stress distribution in the dentin under oblique direction was higher regardless of sealer or technique used.

## Introduction

Vertical root fracture (VRF) is a major clinical problem, characterized by increasing occurrence and a poor prognosis. Whether VRF arises from an endodontic procedure or a post and core restoration, the pressures are expected to be transmitted from the inner root canal to the outer surface of the root [1]. Jamleh et al [2] reported that stresses and strains were increase during canal shaping especially in the apical region of the root. In addition, stress is produced within a structure as a result of internal resistance that opposes the applied force [3]. Notably, stress distribution in dentin plays a central role in causing tooth fractures. Consequently, the most effective approach to reducing damage to a loaded tooth is to minimize strain [4].

Recent studies emphasize the significance of Finite element analysis (FEA) in analysing stress distribution patterns in teeth that have been restored using various prefabricated posts, crowns, intracanal post cementation techniques, and differing tooth lengths [5]. FEA is a computational numerical analysis method that provides a comprehensive approach to calculating complex stress distribution conditions and identifying regions where stress concentration is likely to lead to failure [6].

In a prior FEA investigation conducted by Belli et al. [3], it was observed that when the tooth model was simulated with MTA (Mineral Trioxide Aggregate) filling, stresses at the lingual cervical region decreased. However, stresses within the root structure were found to increase. Another FEA study by Belli et al. [7] found that when an apical bone defect was present, the utilisation of an MTA plug or an MTA-based sealer was found to increase stresses within the root structure, as indicated by a study.

From a biomechanical standpoint, filling the root canals of endodontically treated teeth (ETT) with materials that closely match the stiffness of dentin can help preserve the remaining tooth structure [8]. BioRoot RCS, a calcium silicate root canal sealer (Septodont, Saint Maur des Fosses, France), can be used in two ways: solely as a filling material, which is easier and faster compared to other techniques, or in combination with a gutta percha (GP) point using a single cone technique [9]. Both obturation techniques require a larger amount of sealer compared to other methods, making the properties of the sealer crucial [10]. Therefore, this study aims to assess the impact of BioRoot RCS and AH Plus, with or without GP, on stress distribution in an endodontically treated mandibular premolar. FEA has been employed to accomplish this.

## Methodology

### Finite element model geometry

A three-dimensional finite element model was constructed to simulate a mandibular second premolar tooth and its supporting tissues. Four scenarios were simulated: the tooth filled with gutta percha (GP) in combination with either AH Plus or BioRoot RCS sealer, and the tooth filled with only one of the sealers. A composite resin filling was added to the model to fill the access cavity.

**Fig 1:**
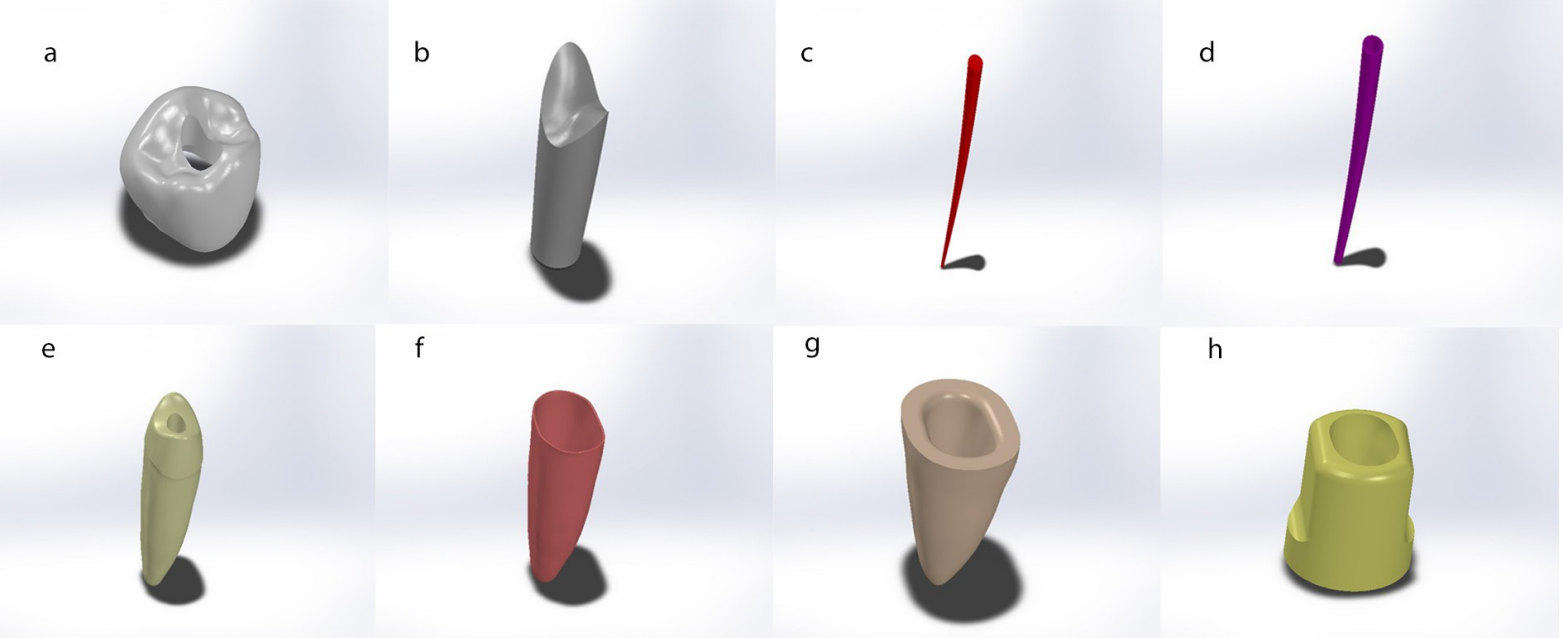
Model geometries from Solid Works screen; (a) Crown, (b) Composite resin, (c) GP, (d) Sealer, (e) Dentine, (f) PDL, (g) Cancellous bone, and (h) Cortical bone.

SolidWorks, an engineering CAD/CAM software developed by SolidWorks Corp, Concord, MA, USA, was used to create the geometries of all the model components. The geometry of the mandibular second premolar tooth used in the model was described by Nelson [11]. Other components such as the periodontal ligament (PDL) and surrounding bone (cortical and cancellous) were also included in the model. Two cylindrical bodies represented the bone, with a height of 22 mm and a diameter of 10 mm. The cortical layer of the bone was 2 mm thick. The apical 10 mm of the root was modelled as being surrounded by a 0.5 mm thick cortical bone socket and had a periodontal ligament thickness of 0.175 mm. The remaining bone was modelled as cancellous bone [12] (Fig 1).

**Fig 2:**
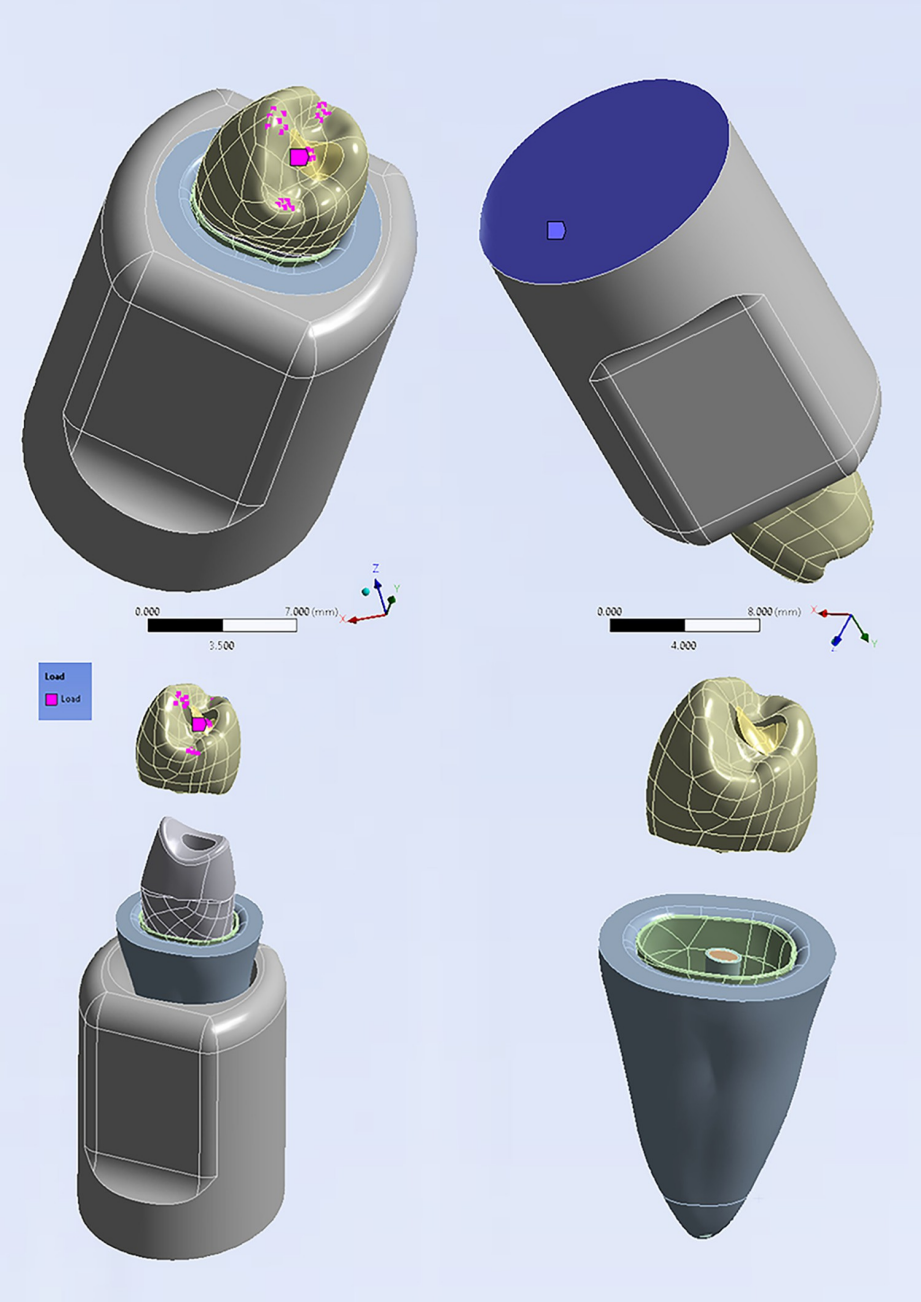
Model description: Assembly, load location and boundary conditions.

The model components were assumed to be firmly connected through bonded contacts to transfer the load between them and ensure their connectivity. Fig 2 illustrates the region where the load was applied and the fixed bottom side of the tooth support structure.

### Material properties

During the analysis using the FEA, it was assumed that the tissues and all materials used in the model were homogeneous, isotropic, and exhibited linear elasticity. The necessary properties for the FEA, namely the elastic modulus (Young’s modulus, E) and Poisson’s ratio (ν), were obtained from various sources in the literature [6, 12–16], and the corresponding values can be found in Table 1.

**Table 1:** Material properties for FEA.

Material	Elastic modulus	Poisson’s ratio	Reference
	(GPa)	Ν	
Enamel	41	0.31	[13]
Composite resin	12	0.3	[6]
Gutta-percha	0.14	0.45	[6]
BioRoot RCS	3.2	0.33	[14, 15]
AH Plus	0.30	0.3	[14, 16]
Dentin	18.6	0.31	[12]
Periodontal ligament	0.0000689	0.45	[13]
Cortical bone	13.7	0.3	[13]
Cancellous bone	1.37	0.3	[13]

### Meshing

**Fig 3:**
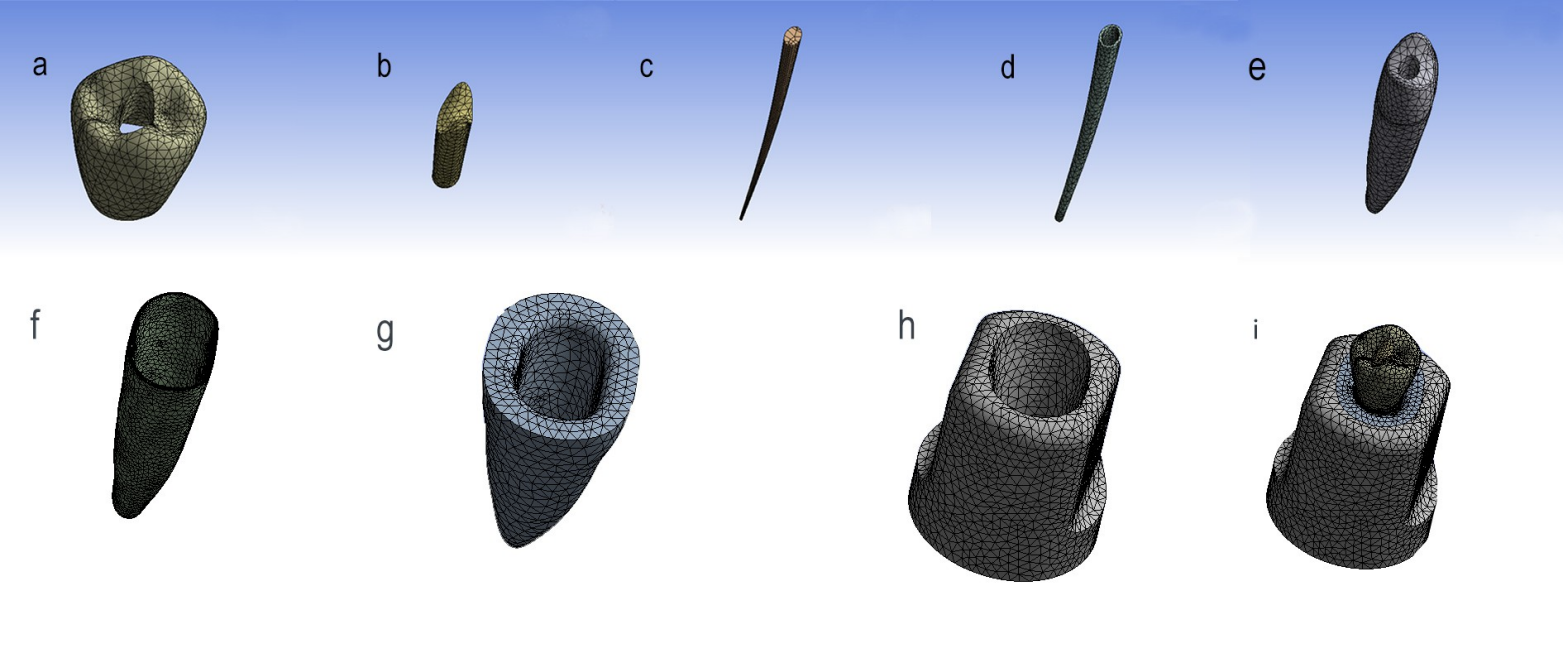
Model components after meshing; (a) Crown, (b) Composite resin, (c) GP, (d) Sealer, (e) Dentine, (f) PDL, (g) Cancellous bone, (h) Cortical bone, and (i)complete model.

Meshing was accomplished by utilizing a 3D tetrahedral quadratic solid element known as "187". This element possesses three degrees of freedom at each node, allowing for translation in the main axes directions. The number of nodes and elements obtained after multiple attempts is presented in Table 2. These numbers were determined by finding a balance between the number of nodes, elements, and the analysis time. Screenshots from the software ANSYS (v.16.0, ANSYS, Inc, PA, USA) screen showcasing the final model components and the meshed model components are provided in Fig 3.

**Table 2:** Approximate number of nodes and elements for meshing.

Component	No of Nodes	No of Elements
Enamel	25,044	16,720
Composite resin	9,915	6,590
Gutta-percha	11,634	2,268
Sealer	13,480	7,151
Dentin	116,928	82,821
Periodontal ligament	36,286	20,654
Cortical bone	31,219	20,398
Cancellous bone	18,477	11,762

### Loads and boundary conditions

In order to replicate the forces exerted during chewing, a static load of 200 N was applied to the tooth in two different directions: (1) vertically and (2) obliquely at a 45° angle from the lingual to the buccal side. This load was applied at three occlusal contact points: the buccal cusp and both marginal ridges. The lowest surface of the model was fixed in place as a boundary condition.

### Case studies

The linear static analyses using the FEA were conducted on an HP Z820 Workstation equipped with Dual Intel Xeon E5-2670 v2 processors, operating at 2.5 GHz, and with 64.0 GB of RAM. A commercially available multipurpose finite element software package, specifically ANSYS version 16.0, was utilized for the analyses. The obtained results were validated by comparing them to findings from similar studies conducted by Benazzi et al. [17] and deMoya et al. [18], and demonstrated a satisfactory level of agreement. Within the scope of this research, eight cases were examined, compared, and extensively discussed in order to derive meaningful conclusions.

Case 1: BioRoot RCS sealer and GP were used under vertical load.

Case 2: BioRoot RCS sealer and GP were used under oblique load.

Case 3: AH Plus sealer and GP were used under vertical load.

Case 4: AH Plus sealer and GP were used under oblique load.

Case 5: BioRoot RCS sealer in bulk under vertical load.

Case 6: BioRoot RCS sealer in bulk under oblique load.

Case 7: AH Plus sealer in bulk under vertical load.

Case 8: AH Plus sealer in bulk under oblique load.

## Results

**Fig 4:**
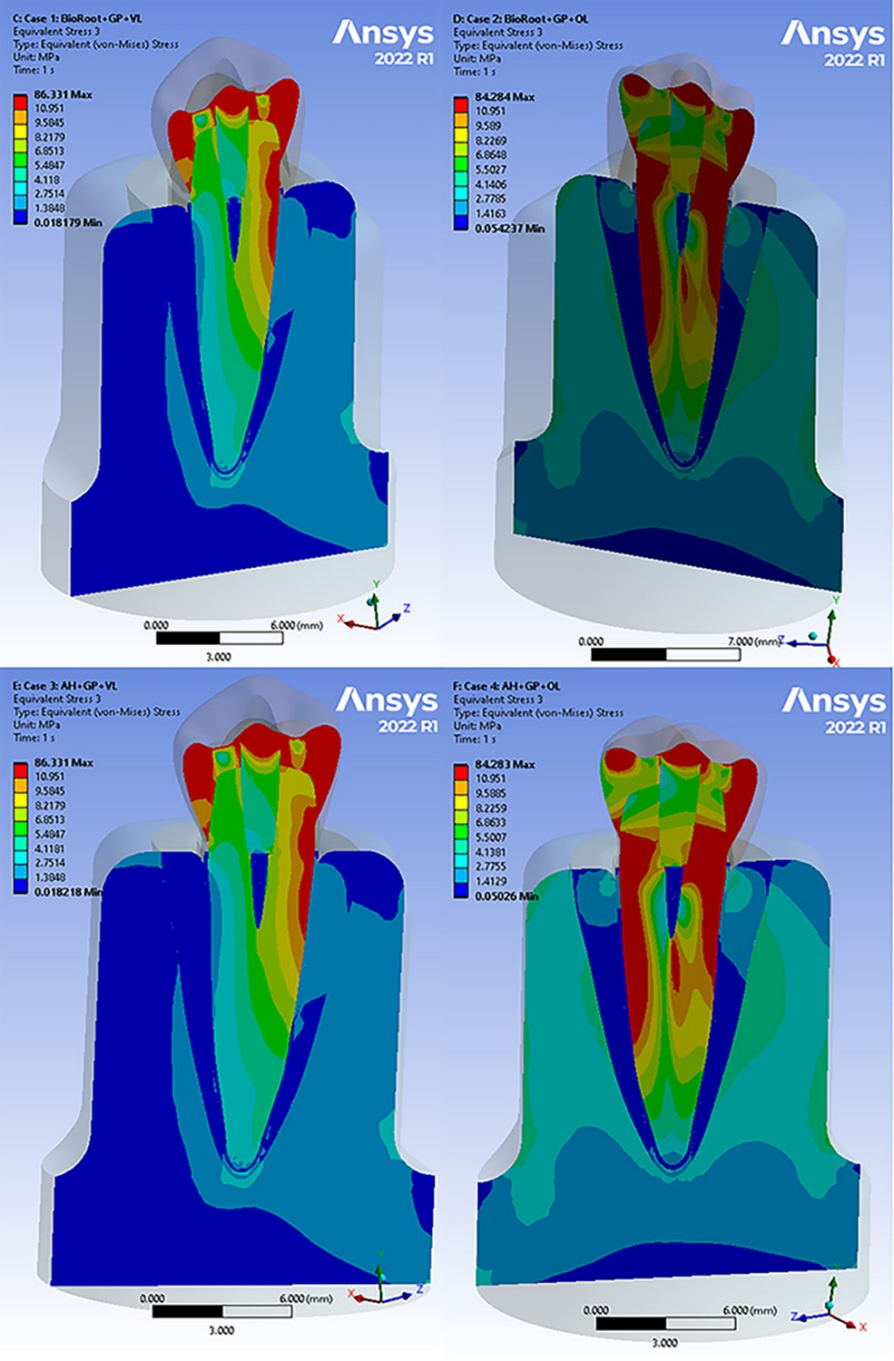
Stress analysis of loading conditions of 200 N vertical and oblique with 45° for BioRoot RCS and AH Plus cases with GP. (a) Von Mises stress in BioRoot RCS with GP under vertical loading (b) Von Mises stress in BioRoot RCS with GP under oblique loading. (c) Von Mises stress in AH Plus with GP under vertical loading (d) Von Mises stress in AH Plus with GP under oblique loading.

**Fig 5:**
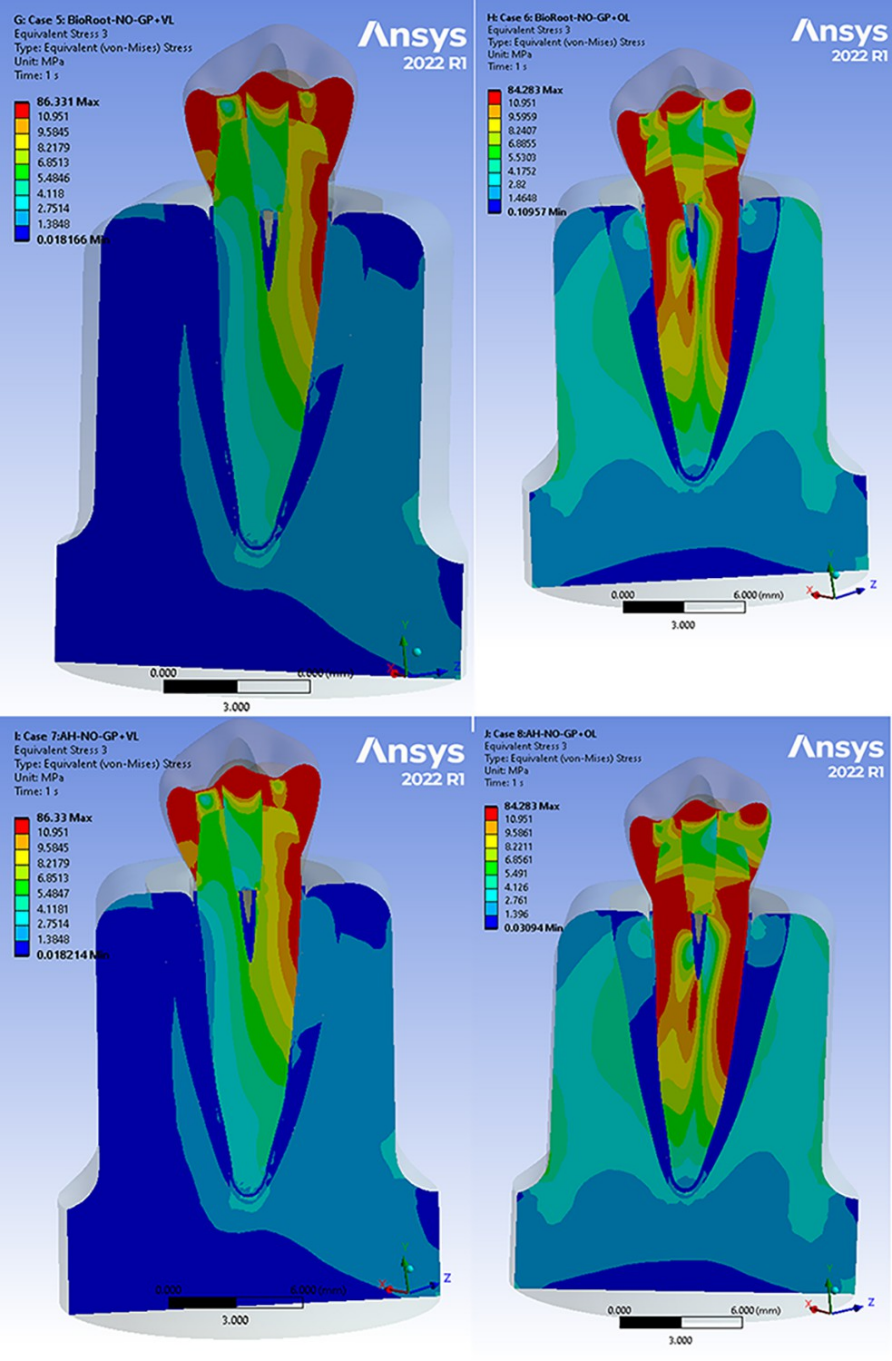
Stress analysis of loading conditions of 200 N vertical and oblique with 45° for BioRoot RCS and AH Plus cases without GP. (a) Von Mises stress in BioRoot RCS only under vertical loading (b) Von Mises stress in BioRoot RCS only under oblique loading. (c) Von Mises stress in AH Plus only under vertical loading (d) Von Mises stress in AH Plus only under oblique loading.

Figs 4 and 5 present the von Mises stress distributions of the models using different root canal sealers across all cases. To analyse the stress distribution and magnitude in each component, the results of all structures were separated from the rest of the model. It was observed that there were no changes in the stress distributions or the locations of maximum and minimum values. Therefore, the extreme von Mises stress values in MPa for each component were compared. When a total occlusal load of 200 N was applied to the functional cusp and marginal ridges, the maximum stress values were found between the dentin and sealers. The stress patterns showed lower values when BioRoot RCS was used compared to AH Plus (Table 3). The sealer material did not affect the von Mises stresses in the periodontal ligament, cancellous bone, and cortical bone. AH Plus experienced less stress than BioRoot RCS, even when the gutta percha was replaced by the sealer material. Additionally, the maximum stress values on the dentin were higher in all AH Plus cases compared to BioRoot RCS (Table 3). While the maximum stress values on the dentin were similar in the BioRoot RCS bulk and BioRoot RCS with GP cases, the stress distribution changed when the load was applied in an oblique direction, resulting in a 10–15% increase in stress levels. Furthermore, it was evident that in the AH Plus bulk cases, the maximum stress values on the dentin were higher compared to AH Plus with GP.

**Table 3:** Maximum von Mises stresses in MPa in dentin and sealer, GP: Gutta percha, VL: Vertical load, OL: Oblique load.

CASE	Dentin	Sealer
BioRoot RCS +GP+VL	51.593	1.2328
BioRoot RCS +GP+OL	61.8	2.7821
AH Plus +GP+VL	73.87	0.13125
AH Plus +GP+OL	88.361	0.25283
BioRoot RCS +VL	51.982	1.2895
BioRoot RCS +OL	61.803	2.8691
AH Plus +VL	76.197	0.12056
AH Plus +OL	91.169	0.28578

**Fig 6:**
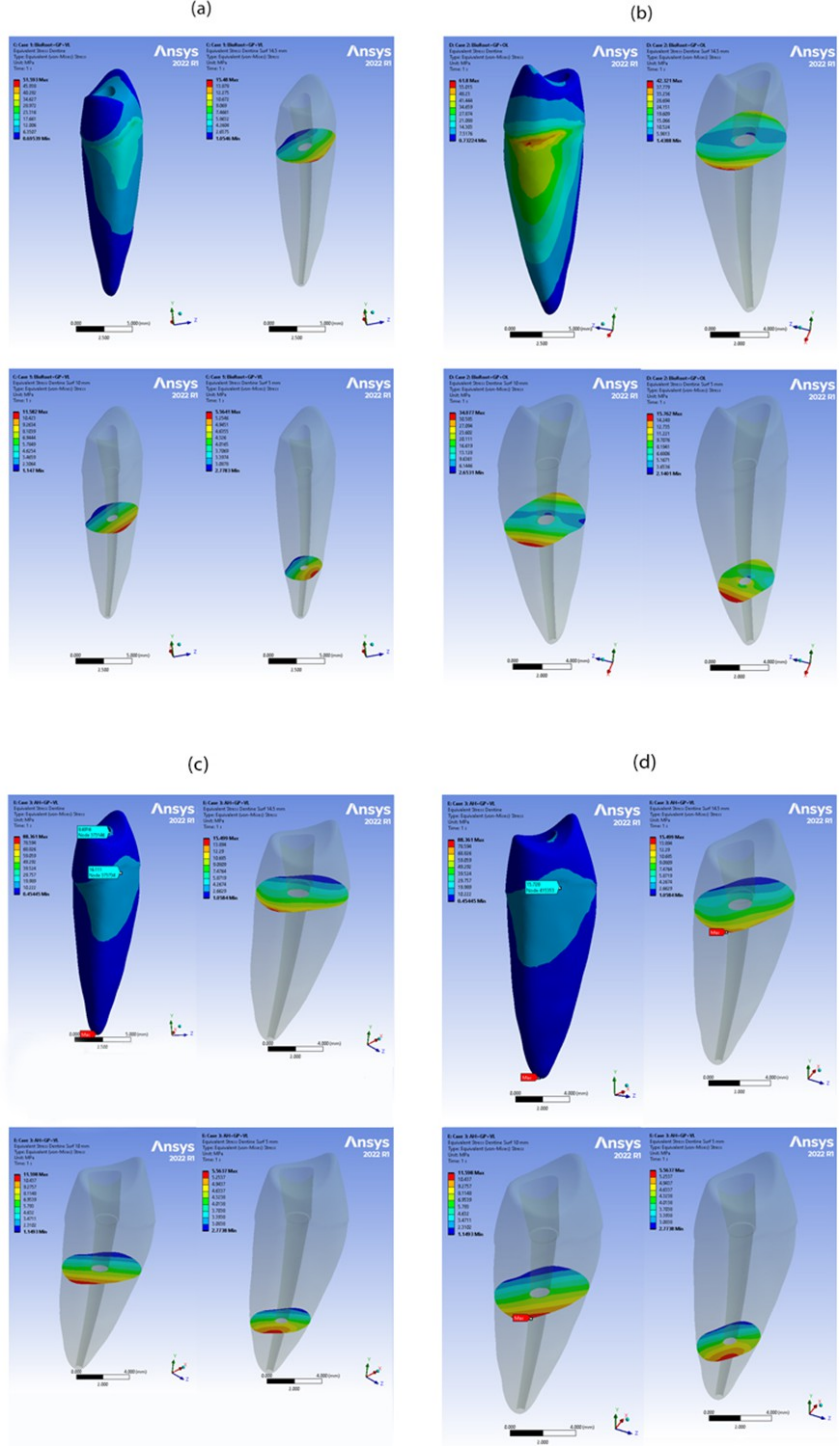
Stress analysis of loading conditions of 200 N vertical and oblique with 45° for all BioRoot RCS cases in different root levels. Highest and lowest stress values are indicated by red and blue colours, respectively. (a) Von Mises stress in BioRoot RCS with GP under vertical loading (b) Von Mises stress in BioRoot RCS with GP under oblique loading (c) Von Mises stress in BioRoot RCS only under vertical loading (d) Von Mises stress in BioRoot RCS only under oblique loading.

**Fig 7:**
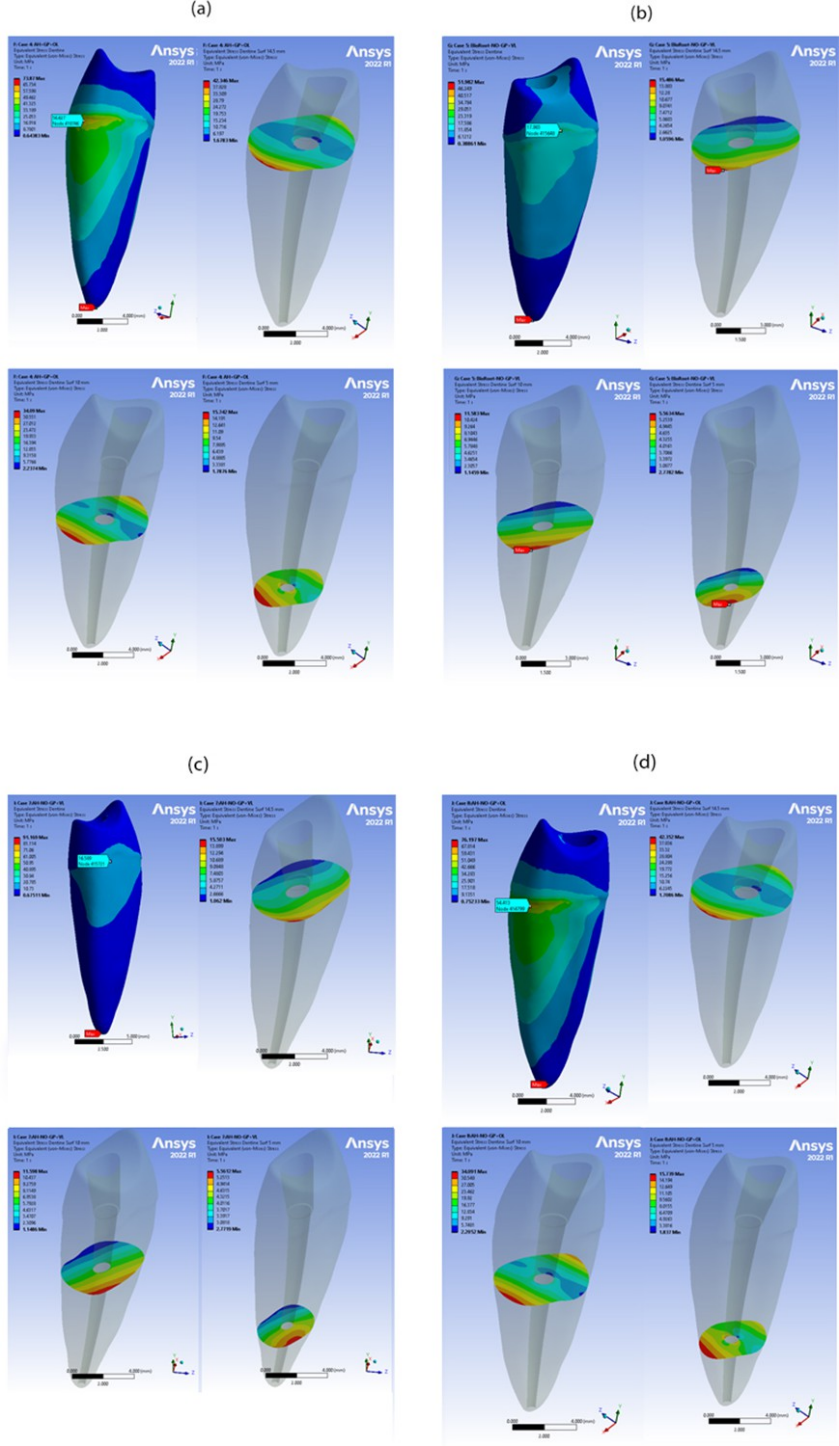
Stress analysis of loading conditions of 200 N vertical and oblique with 45° for all AH Plus cases in different root levels. Highest and lowest stress values are indicated by red and blue colours, respectively. (a) Von Mises stress in AH Plus with GP under vertical loading (b) Von Mises stress in AH Plus with GP under oblique loading (c) Von Mises stress in AH Plus only under vertical loading (d) Von Mises stress in AH Plus only under oblique loading.

Examining the cross-section of the root revealed that the maximum von Mises stresses were in the coronal thirds, while the lowest stresses were in the apical thirds for all sealers (Figs 6 and 7). It should be noted that all the obtained values of deformations, stresses, and strains were within the acceptable range for the proposed materials under both types of loading. Therefore, no sudden failure should occur under these loading conditions.

## Discussions

FEA is an approximate numerical technique that offers detailed qualitative information about the interaction between restorative materials and the surrounding tooth structure [19]. One advantage of FEM is its flexibility in adjusting various conditions, allowing estimation of stress distribution around model components in clinically challenging sites [20]. Moreover, FEM can provide numerical estimates of the effects of new materials or treatments on natural tissues before clinical testing, suggesting potential improvements [21]. Root canal sealers must possess biocompatibility as a crucial requirement. Additionally, they should exhibit reasonable compressive strength and hardness to withstand occlusal forces [22]. BioRoot RCS is a calcium silicate–based root canal sealer mainly composed of tricalcium silicate and zirconium oxide powder, which requires mixing with a liquid containing calcium chloride. After setting, BioRoot RCS releases calcium hydroxide and exhibits high levels of calcium leaching [23]. On the other hand, AH Plus, an epoxy resin–based sealer (Dentsply DeTrey GmbH, Constanz, Germany), is commonly regarded as the standard sealer due to its strong bond strength to dentin compared to other root canal sealers [24]. AH Plus is frequently used because of its solubility, resistance to disintegration [25], and satisfactory dimensional stability [26].

This study aimed to assess stress distributions in root dentin following simulated root canal treatment using AH Plus and BioRoot RCS. The objective was to identify areas of stress concentration that could potentially lead to fractures. To achieve this, a 3D elastic FEA was conducted, enabling examination of all aspects of root dentin that are typically inaccessible in vivo. The analysis of biomechanical responses in tooth structures is a complex task due to the intricate nature of biomaterials, dental anatomy, and microstructural characteristics. The FEA provides a suitable and analytical approach to assess the biomechanical behaviours in complex geometries, as it standardizes parameters and allows for accurate evaluations [27]. FEA models can be either 2D or 3D, but 2D models have limitations in capturing out-of-plane deformations, strains, and stresses, leading to errors in the analysis due to artificial constraints. Therefore, the utilization of 3D models for analysing biological or biocompatible structures yields more realistic results compared to 2D models [28]. In addition, it is important to note that improving the quality of the mesh has a beneficial effect on the accuracy of the results by reducing the impact of sharp edges. However, increasing the density of the mesh prolongs the time needed for analysis. The stress values were depicted as von Mises stresses in our analysis. Von Mises stress is a composite measure that combines the magnitudes of stresses along the x-, y-, and z-axes, providing insight into the locations of maximum stress concentration [29]. This measure is valuable for identifying areas that are prone to potential damage [30].

This study focused on investigating the highest stress values in dentin and sealers. Identifying these maximum stress values in dentin tissue and their corresponding locations is of clinical significance, as they can potentially lead to the formation of cracks and fractures, affecting the long-term integrity of the tooth [15]. Jiang et al. emphasized that stress concentrations in specific regions indicate the location and probability of initial failure, thus influencing the tooth’s fracture resistance [31]. Consequently, we conducted an examination specifically targeting the highest stress areas and values.

Our study employed both vertical and oblique occlusal directions to simulate the complex dynamics that occur during functional chewing in individuals. By including an oblique load, we aimed to replicate the effects of parafunctional forces in conditions such as bruxism and the consumption of hard food items. It is important to note that the distribution of stress within the models is influenced by the direction and position of the applied load [32]. To simulate normal occlusal forces, a load of 200 N was applied to the crown at the contact points on the occlusal surface [33]. The findings indicate that the choice of core material did not affect the maximum von Mises stress levels in dentin for cases filled with BioRoot RCS, whether it was used in bulk or in combination with the core material. However, when AH Plus was used, the presence of a core material resulted in lower maximum von Mises stress levels in dentin compared to AH Plus used in bulk. One possible explanation for this outcome is that gutta-percha (GP), being a low-modulus material, is capable of absorbing some of the generated stress [34]. On the other hand, the calcium silicate-based sealer, having a higher elastic modulus than the epoxy resin-based sealer, can effectively distribute the stresses within its own structure, leading to a more uniform stress distribution observed in tooth models filled with BioRoot RCS [7]. In both vertical and oblique load directions during mastication simulation, the dentin maximum von Mises stress levels were found to be lower in cases filled with BioRoot RCS compared to cases filled with AH Plus. This outcome can be attributed to the unique properties of BioRoot RCS, such as its high tensile strength and elastic modulus similar to dentin, which contribute to lower stress levels at the interface [35]. When a root is filled with BioRoot RCS, the material mimics dentin by effectively containing stresses within itself and promoting a homogeneous stress distribution [7]. Conversely, the low elastic modulus of AH Plus is believed to provide insufficient support and generate more stress in the surrounding structure. These findings align with a previous FEA study conducted by Eram et al., which demonstrated lower stress levels in teeth filled with calcium silicate materials [36]. Another study reported that Biodentine models exhibited lower maximum von Mises stress values compared to MTA models [15].

The FEA images revealed that cases subjected to vertical loading exhibited lower levels of stress distribution compared to cases subjected to oblique loading at the root dentin. This difference can be attributed to the vertical load being divided between the buccal and palatal cusps, while the oblique load was directly applied to the palatal cusp [37]. This finding aligns with previous research [17], which also reported similar results. Furthermore, it has been documented that oblique forces generate higher levels of stress compared to vertical loading [38]. The evaluation of outcome measures was performed at different distances from the cementoenamel junction (CEJ), specifically at 2, 4, and 6 mm. The maximum von Mises stress values were consistently observed in the coronal third of all samples, and these values decreased gradually towards the apical region of the root, regardless of the type of sealer used. When examining the isolated sealer using FEA, the highest stress levels were found in proximity to the area where the load was applied, followed by a stepwise reduction towards the opposite side. This finding is in line with the research conducted by Brito-Júnior et al., who reported that the coronal third exhibited the highest maximum stress values, while the apical third had the lowest values in root canals filled with AH Plus sealer [16].

Structural failure or fracture occurs when the von Mises stress values surpass the yield strength of the tooth, as highlighted in previous research [39]. The location of the maximum von Mises stress in the FEA model indicates the area where stress concentration is likely to initiate a crack [40]. However, our numerical simulations demonstrated that the different material properties utilized in this dental structure had no impact on the current investigation, as the maximum calculated stress values remained well below the reported median tensile strength of dentin. Nevertheless, further studies are required to simulate more challenging clinical scenarios, such as badly distractive crown involves the marginal ridges can help to replicate complex and demanding conditions encountered in real-clinical dental practice, involving the use of calcium silicate-based sealers in bulk as an obturation technique.

## Conclusion

Based on the constraints of this FEA analysis, the following findings were derived:

1- When subject to a total occlusal load of 200 N on the functional cusp and marginal ridges, the von Mises stresses in root dentin were higher in AH Plus cases compared to BioRoot RCS cases.
2- Shifting the load to an oblique direction resulted in a complete alteration of the stress results, with increased stress levels observed in all cases.
3- The dentin maximum von Mises stress was similar whether BioRoot RCS was used with a core material or in bulk form.

## Supporting information

S1 File Finite element data (construction, vertical and oblique load, and stress distribution). (PDF)
