# Supplementary material for: Evaluation of stress distributions of calcium silicate-based root canal sealer in bulk or with main core material: A finite element analysis study
Source: PLoS One. 2024 Mar 14;19(3):e0299552. doi: 10.1371/journal.pone.0299552 (PMC10939189; doi:10.1371/journal.pone.0299552)
Supplement: S1 File — (PDF) [file pone.0299552.s001.pdf]

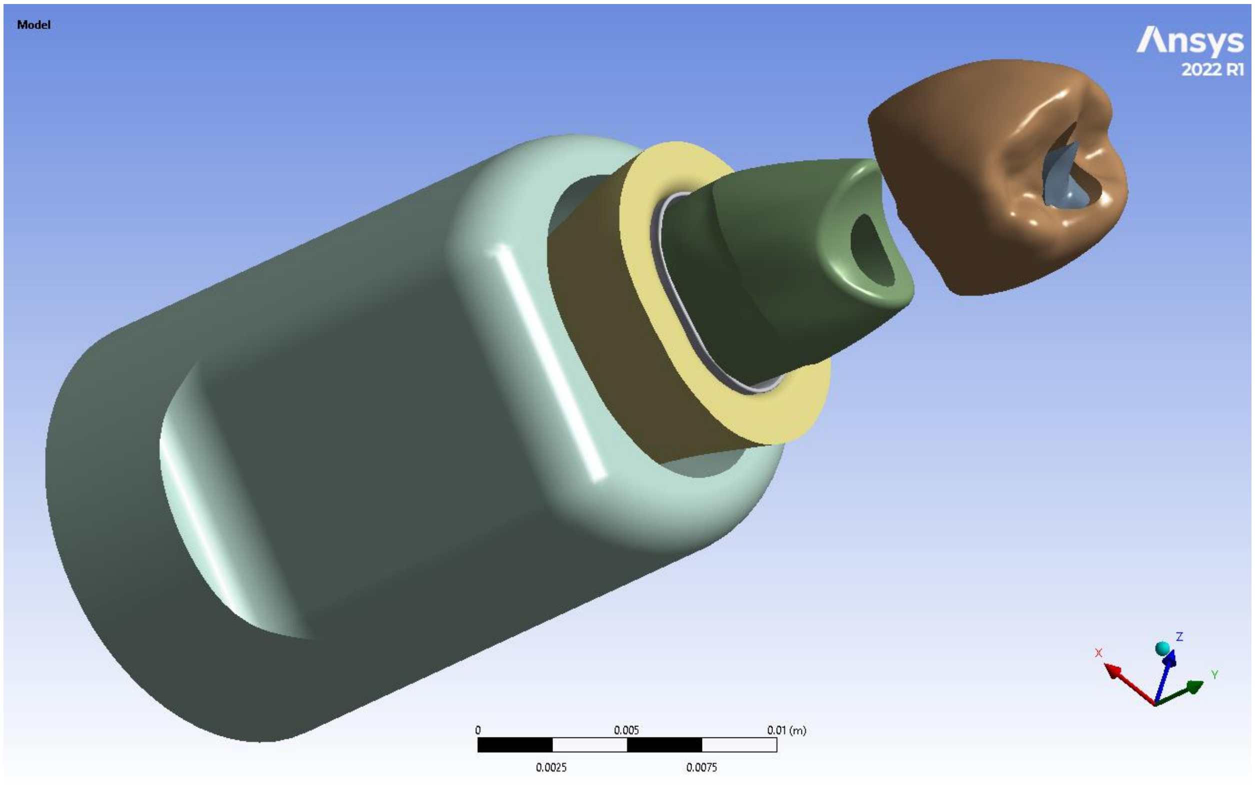

Model
Ansys
2022 R1
0
0.005
0.01 (m)
0.0025
0.0075
X
Y
Z

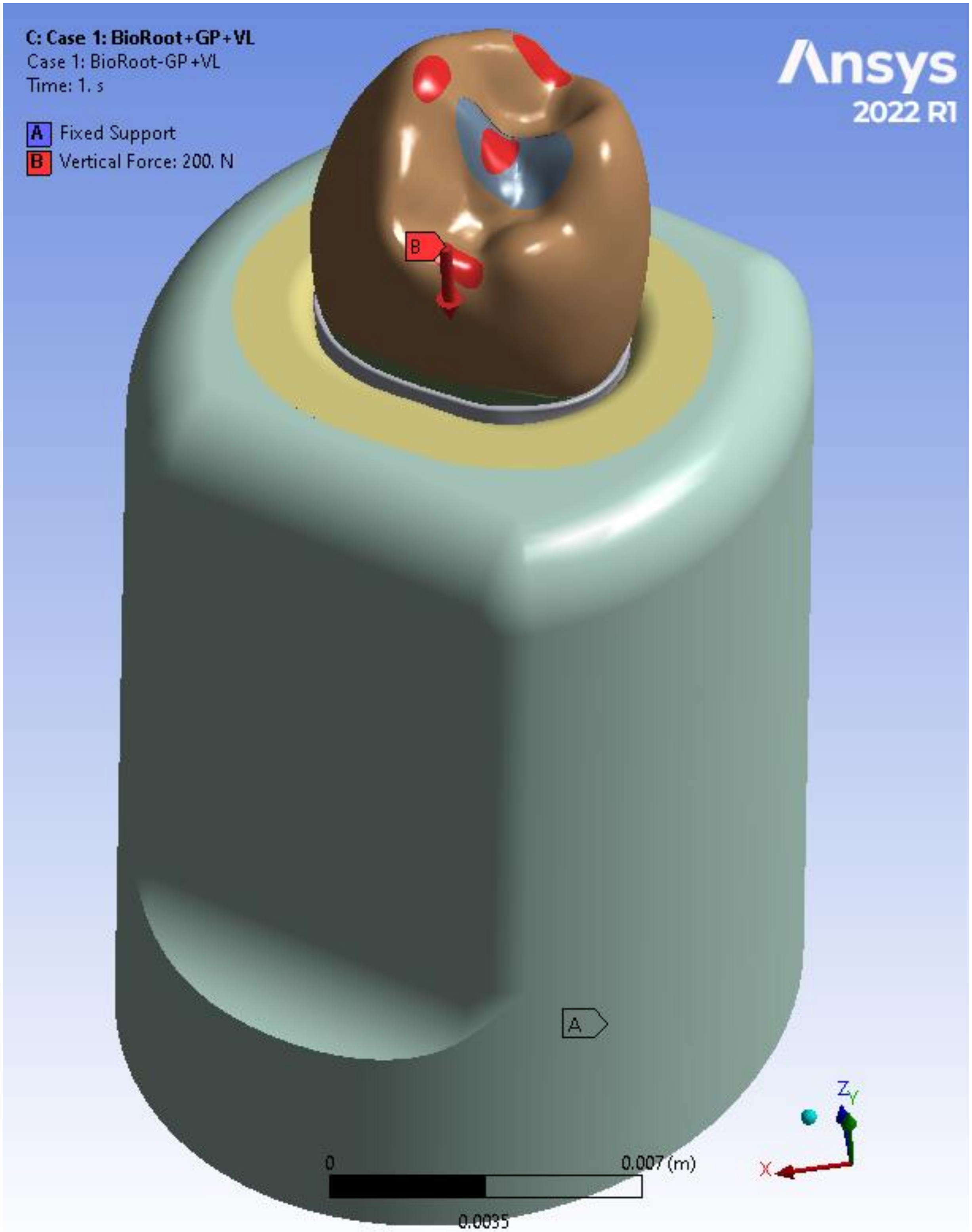

C: Case 1: BioRoot+GP+VL
Case 1: BioRoot-GP+VL
Time: 1. s
A Fixed Support
B Vertical Force: 200. N
Ansys
2022 R1
B
A
0
0.007 (m)
0.0035
Z
Y
X

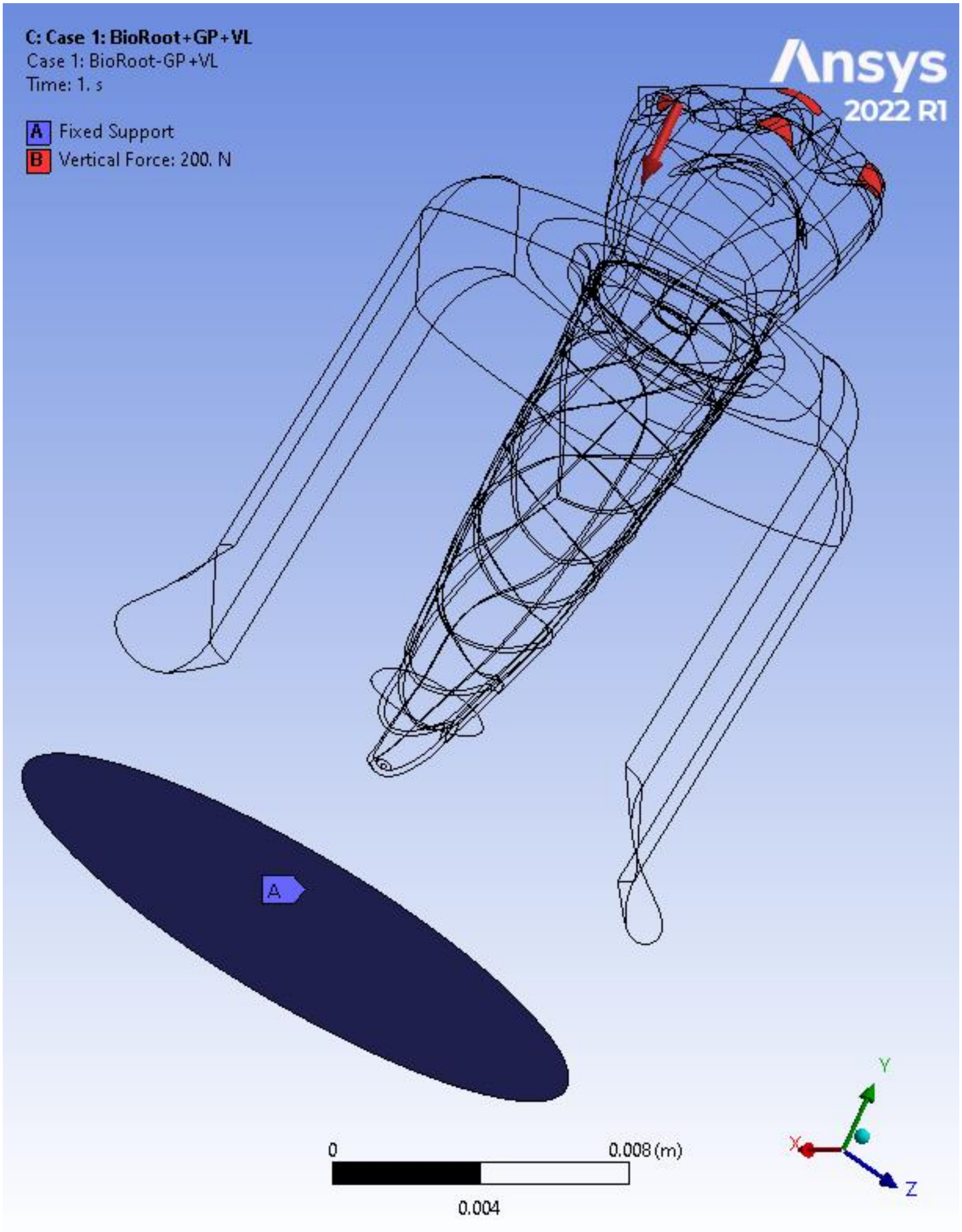
C: Case 1: BioRoot+GP+VL
Case 1: BioRoot-GP+VL
Time: 1. s
A Fixed Support
B Vertical Force: 200. N
Ansys
2022 R1
A
0
0.008 (m)
0.004
Y
X
Z

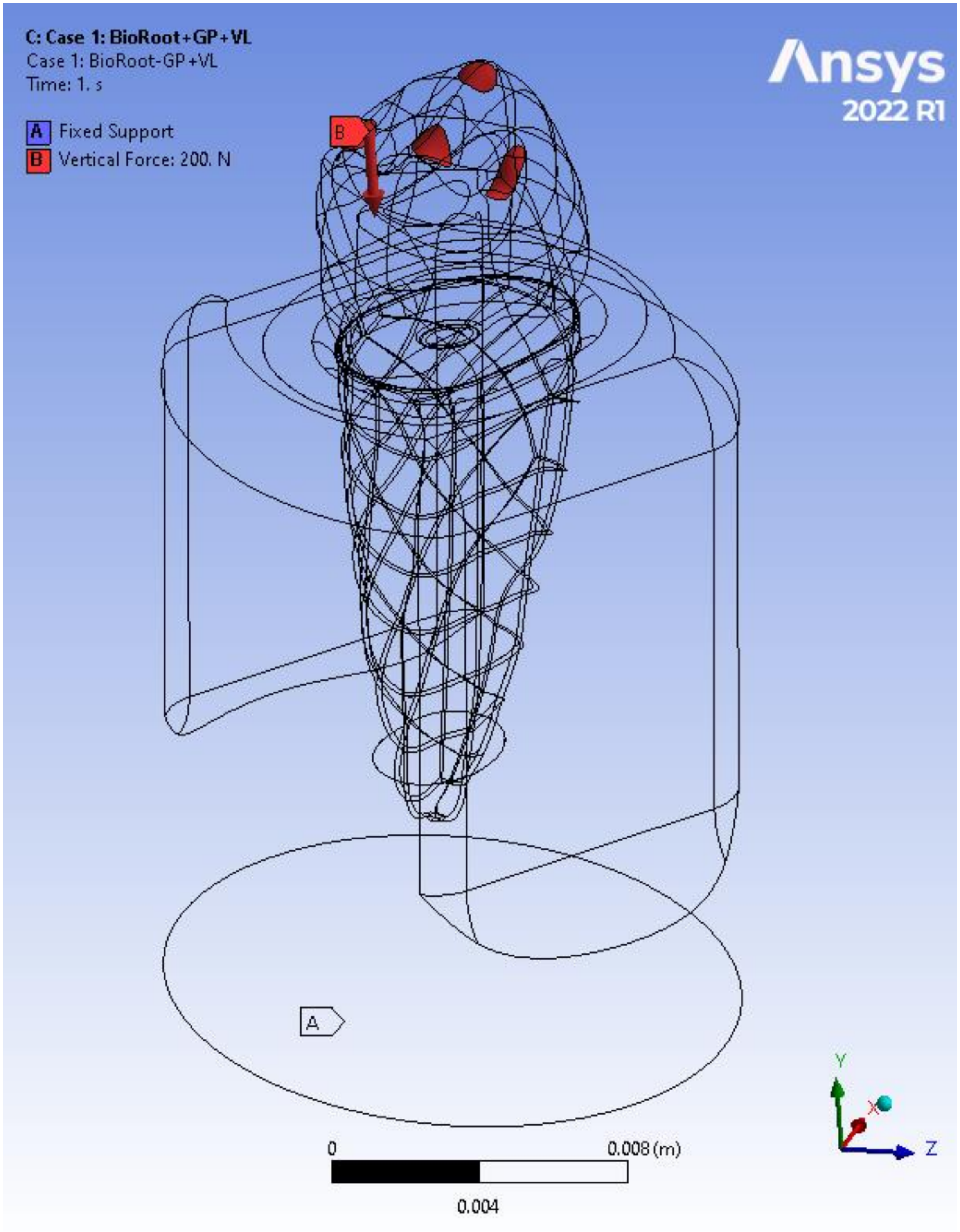
C: Case 1: BioRoot+GP+VL
Case 1: BioRoot-GP+VL
Time: 1. s
A Fixed Support
B Vertical Force: 200. N
Ansys
2022 R1
B
A
Y
X
Z
0
0.008 (m)
0.004

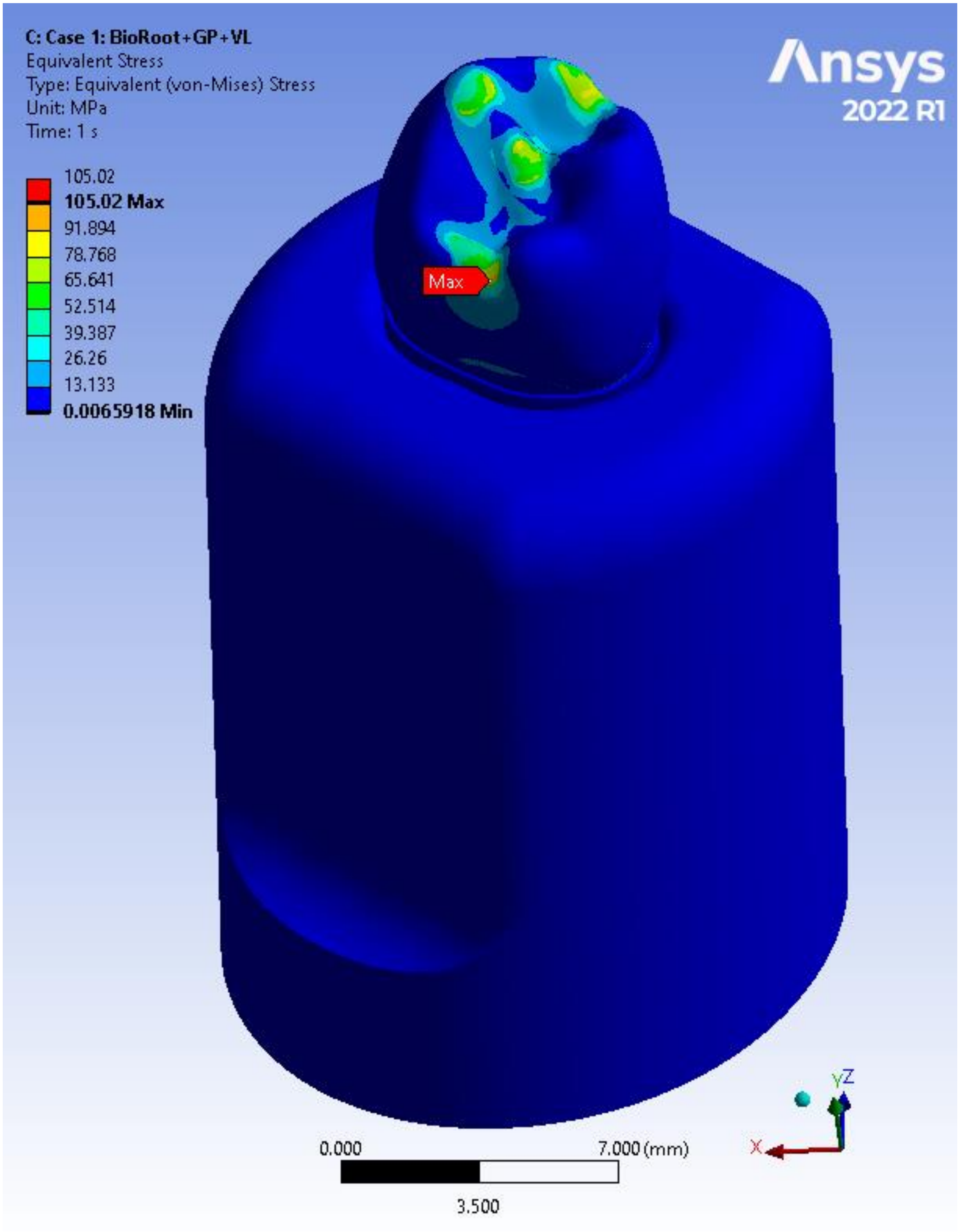

C: Case 1: BioRoot+GP+VL
Equivalent Stress
Type: Equivalent (von-Mises) Stress
Unit: MPa
Time: 1 s
105.02
105.02 Max
91.894
78.768
65.641
52.514
39.387
26.26
13.133
0.0065918 Min
Ansys
2022 R1
Max
0.000
7.000 (mm)
3.500
Y
Z
X

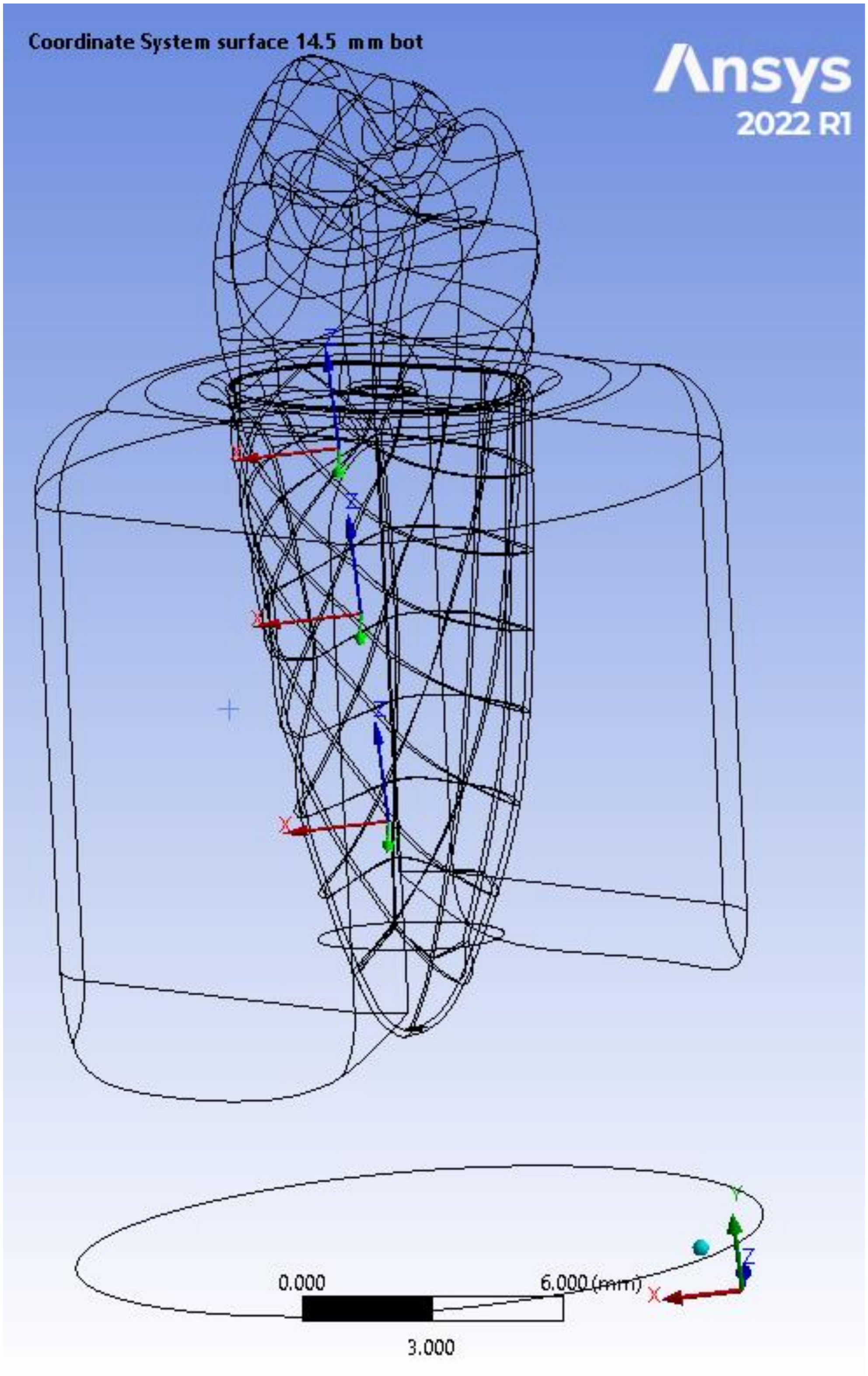

Coordinate System surface 14.5 mm bot
Ansys
2022 R1
0.000
6.000 (mm)
3.000

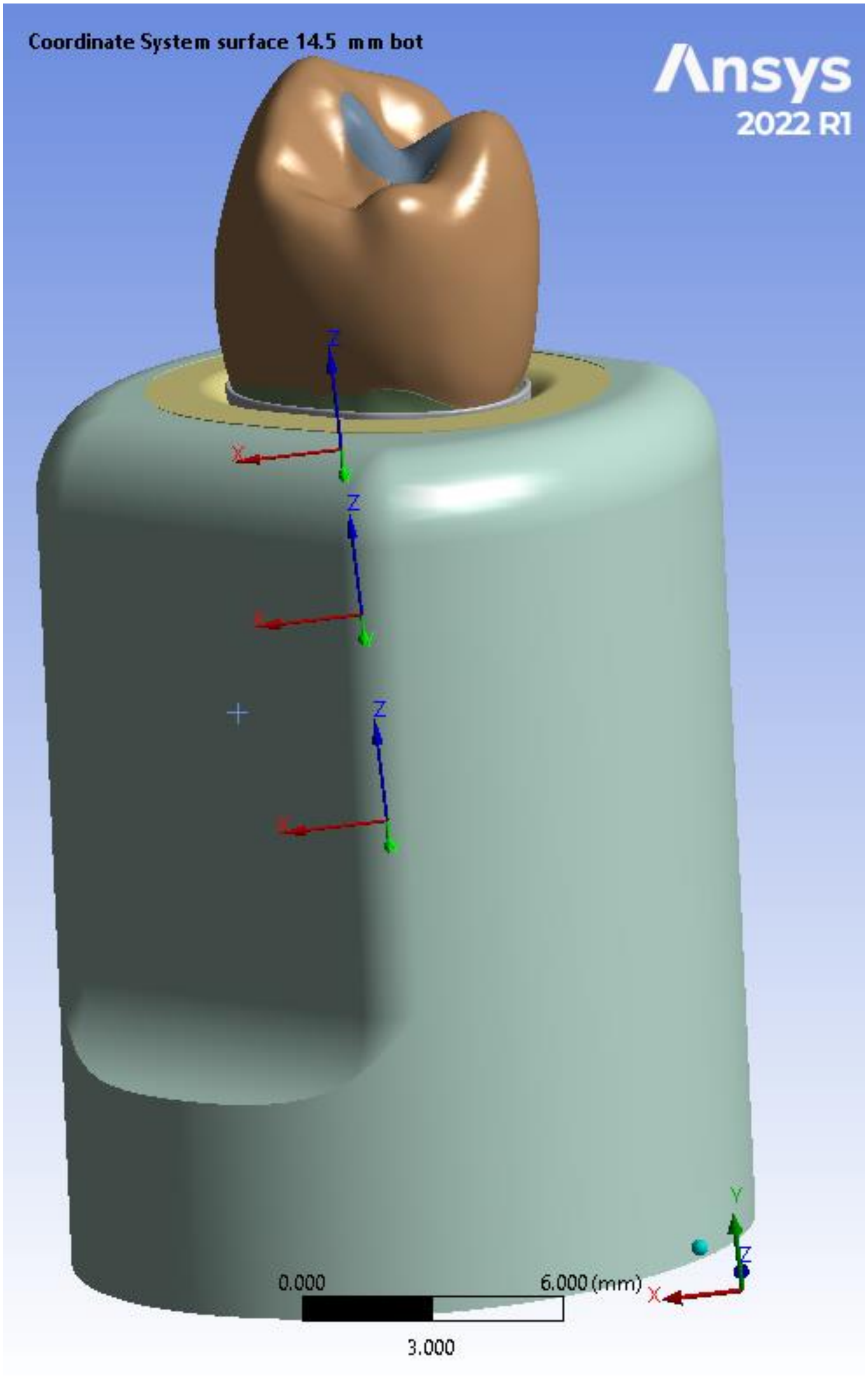

Coordinate System surface 14.5 mm bot
Ansys
2022 R1
0.000
6.000 (mm)
3.000

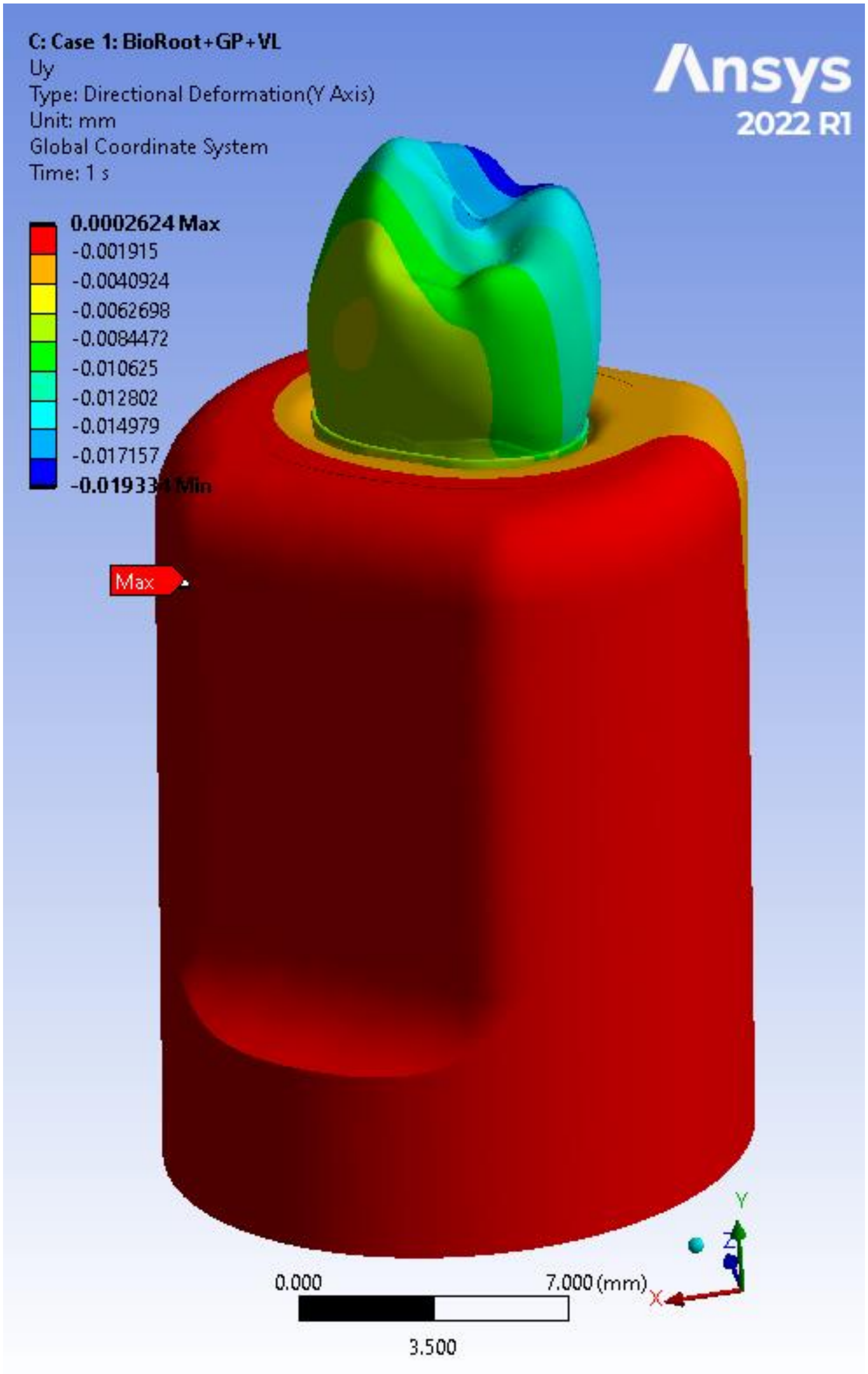
C: Case 1: BioRoot+GP+VL
Uy
Type: Directional Deformation(Y Axis)
Unit: mm
Global Coordinate System
Time: 1 s
0.0002624 Max
-0.001915
-0.0040924
-0.0062698
-0.0084472
-0.010625
-0.012802
-0.014979
-0.017157
Min
Max
Ansys
2022 R1
0.000
7.000 (mm)
3.500
Y
Z
X

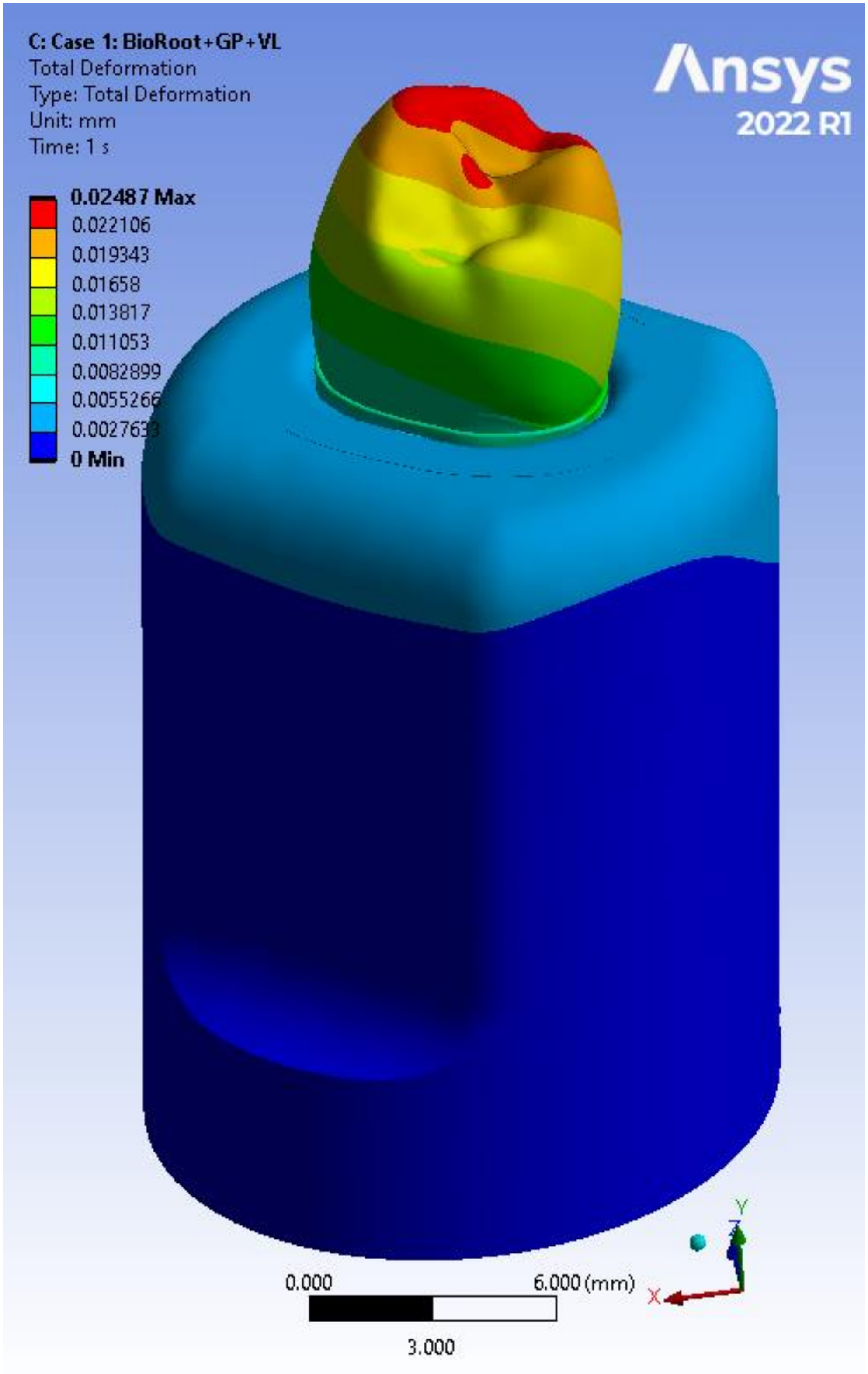

C: Case 1: BioRoot+GP+VL
Total Deformation
Type: Total Deformation
Unit: mm
Time: 1 s
0.02487 Max
0.022106
0.019343
0.01658
0.013817
0.011053
0.0082899
0.0055266
0.0027633
0 Min
Ansys
2022 R1
0.000
6.000 (mm)
3.000
Y
Z
X

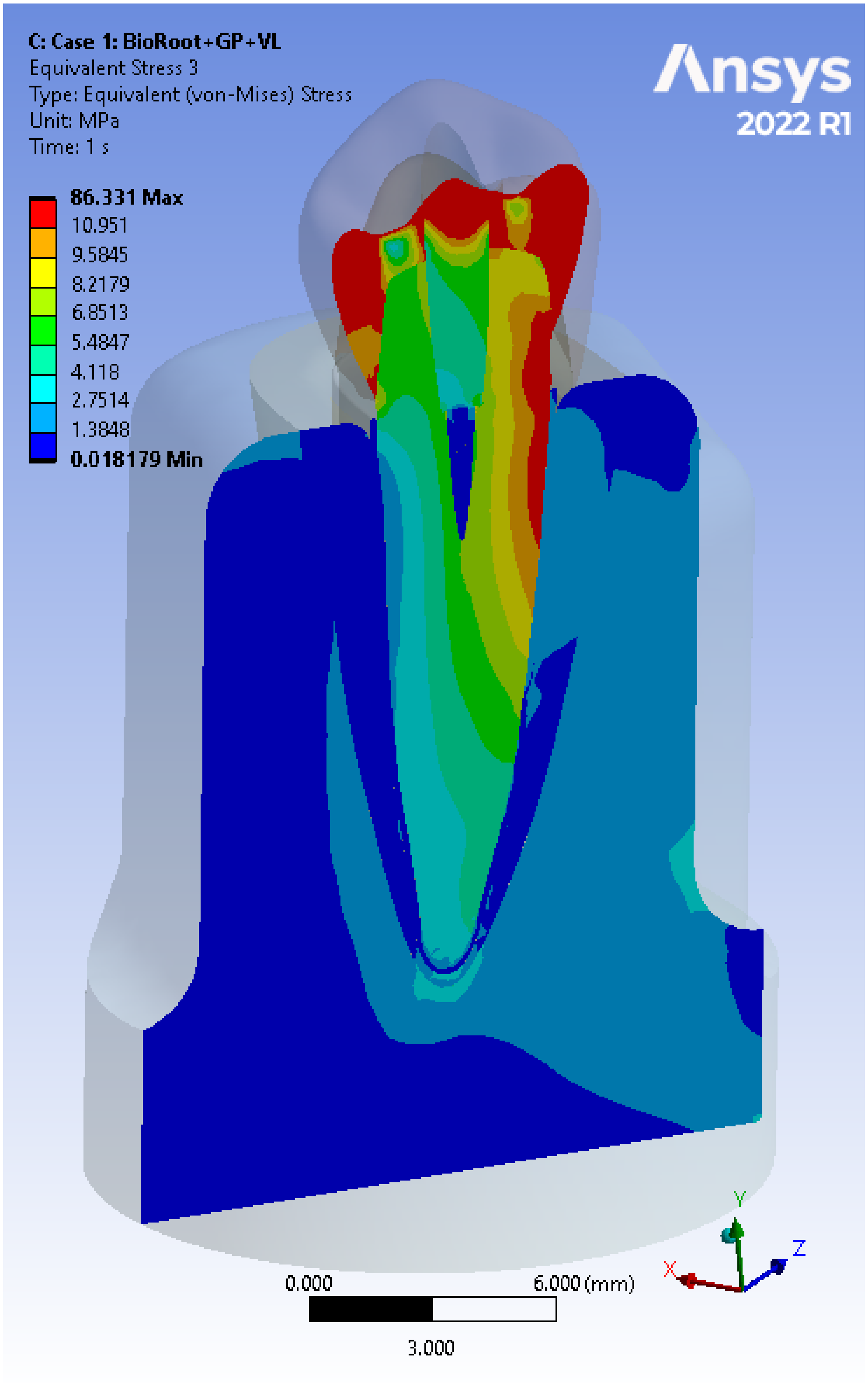
C: Case 1: BioRoot+GP+VL
Equivalent Stress 3
Type: Equivalent (von-Mises) Stress
Unit: MPa
Time: 1 s
86.331 Max
10.951
9.5845
8.2179
6.8513
5.4847
4.118
2.7514
1.3848
0.018179 Min
Ansys
2022 R1
0.000
6.000 (mm)
3.000
Y
Z
X

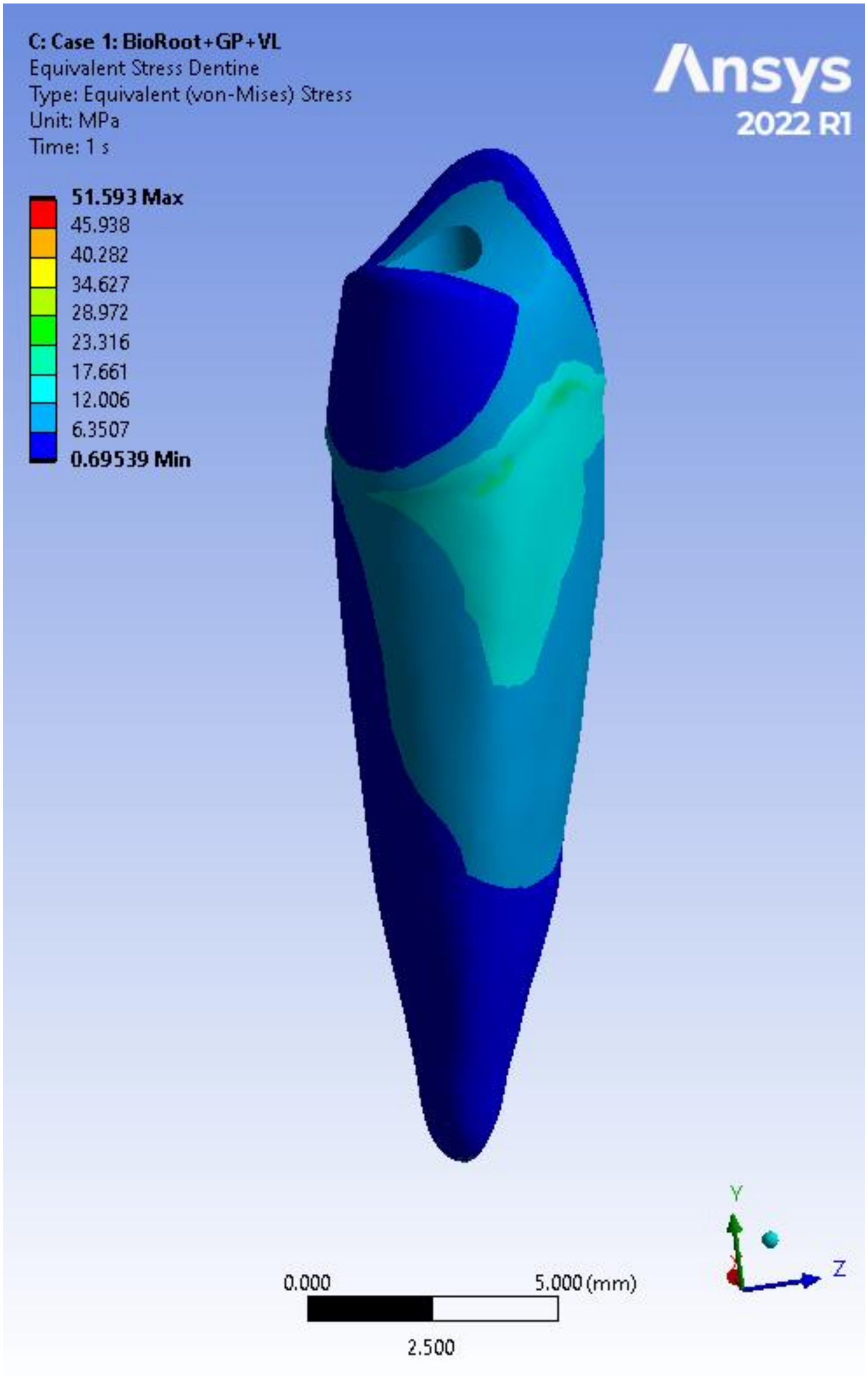
C: Case 1: BioRoot+GP+VL
Equivalent Stress Dentine
Type: Equivalent (von-Mises) Stress
Unit: MPa
Time: 1 s
51.593 Max
45.938
40.282
34.627
28.972
23.316
17.661
12.006
6.3507
0.69539 Min
Ansys
2022 R1
0.000
5.000 (mm)
2.500
Y
Z

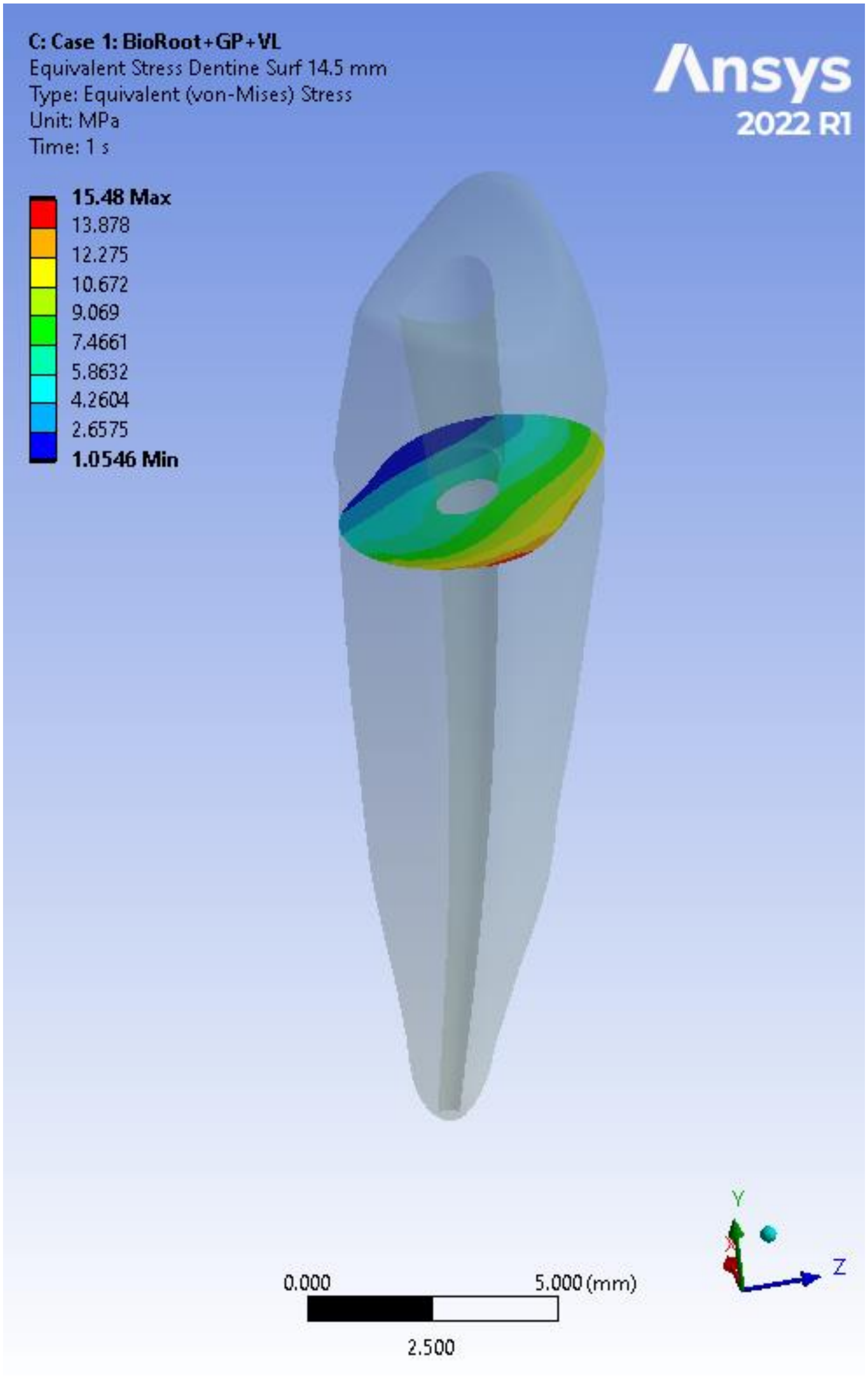
C: Case 1: BioRoot+GP+VL
Equivalent Stress Dentine Surf 14.5 mm
Type: Equivalent (von-Mises) Stress
Unit: MPa
Time: 1 s
15.48 Max
13.878
12.275
10.672
9.069
7.4661
5.8632
4.2604
2.6575
1.0546 Min
Ansys
2022 R1
0.000
5.000 (mm)
2.500
Y
X
Z

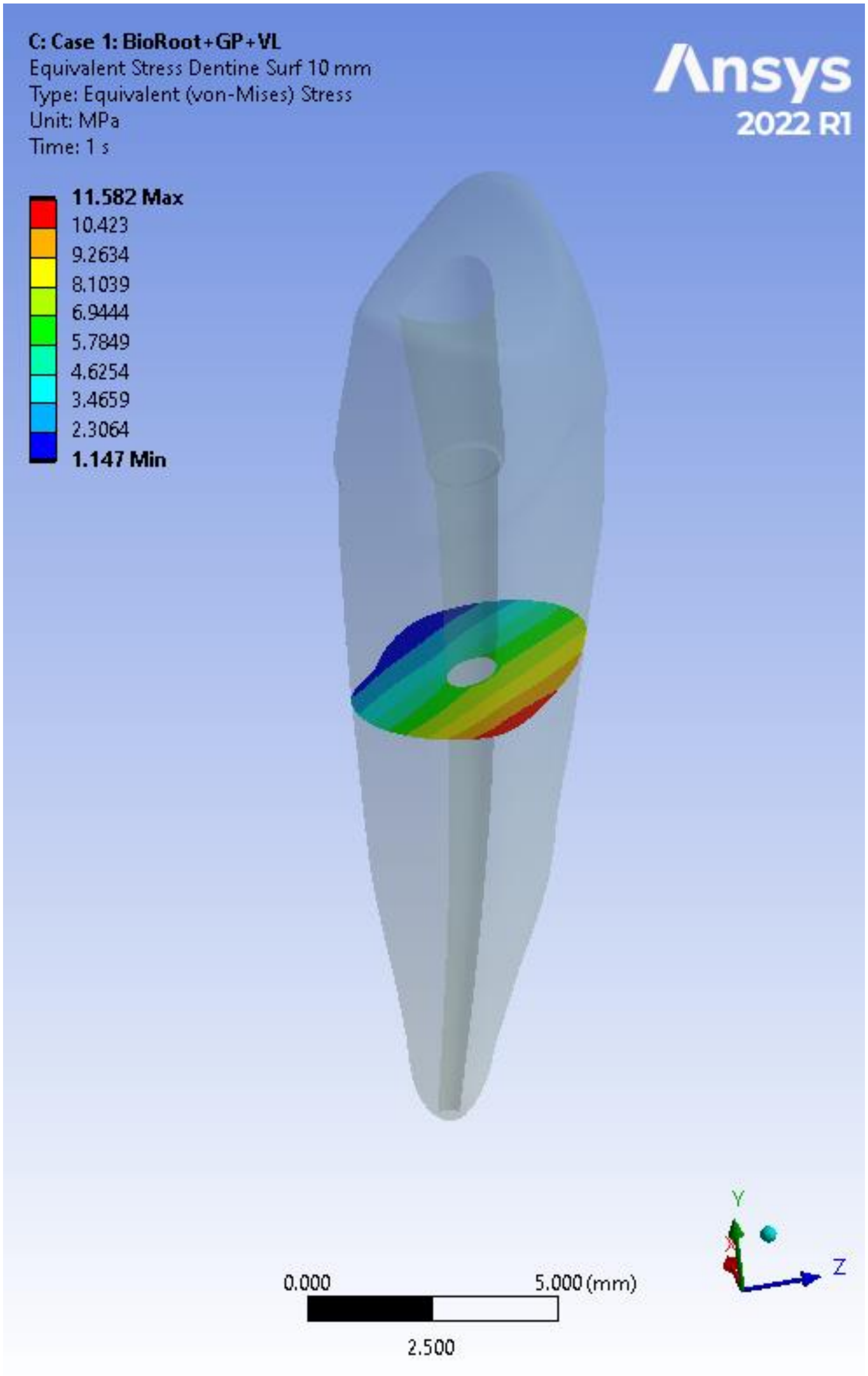
C: Case 1: BioRoot+GP+VL
Equivalent Stress Dentine Surf 10 mm
Type: Equivalent (von-Mises) Stress
Unit: MPa
Time: 1 s
Ansys
2022 R1
11.582 Max
10.423
9.2634
8.1039
6.9444
5.7849
4.6254
3.4659
2.3064
1.147 Min
0.000
5.000 (mm)
2.500
Y
X
Z

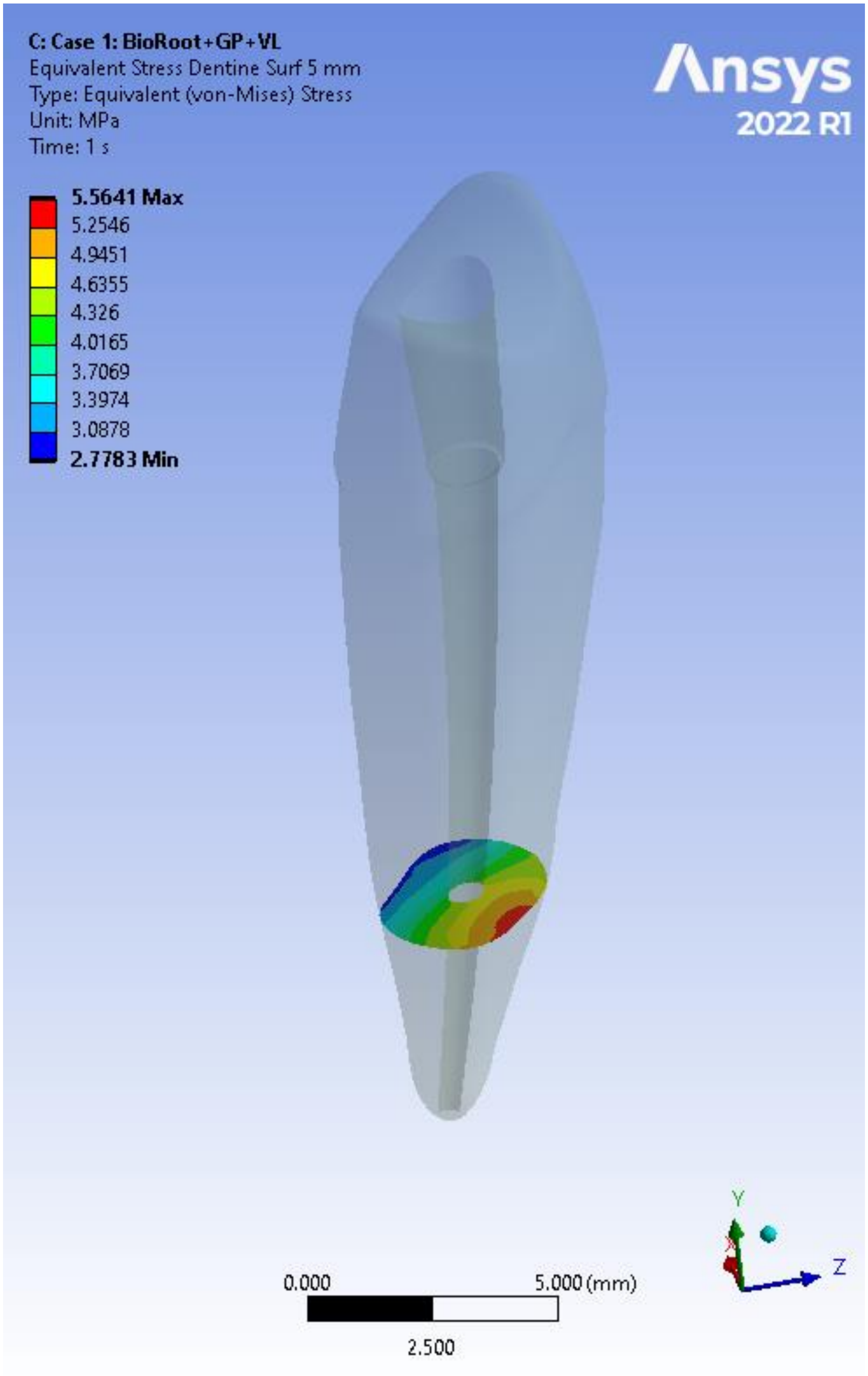
C: Case 1: BioRoot+GP+VL
Equivalent Stress Dentine Surf 5 mm
Type: Equivalent (von-Mises) Stress
Unit: MPa
Time: 1 s
5.5641 Max
5.2546
4.9451
4.6355
4.326
4.0165
3.7069
3.3974
3.0878
2.7783 Min
Ansys
2022 R1
0.000
5.000 (mm)
2.500
Y
X
Z

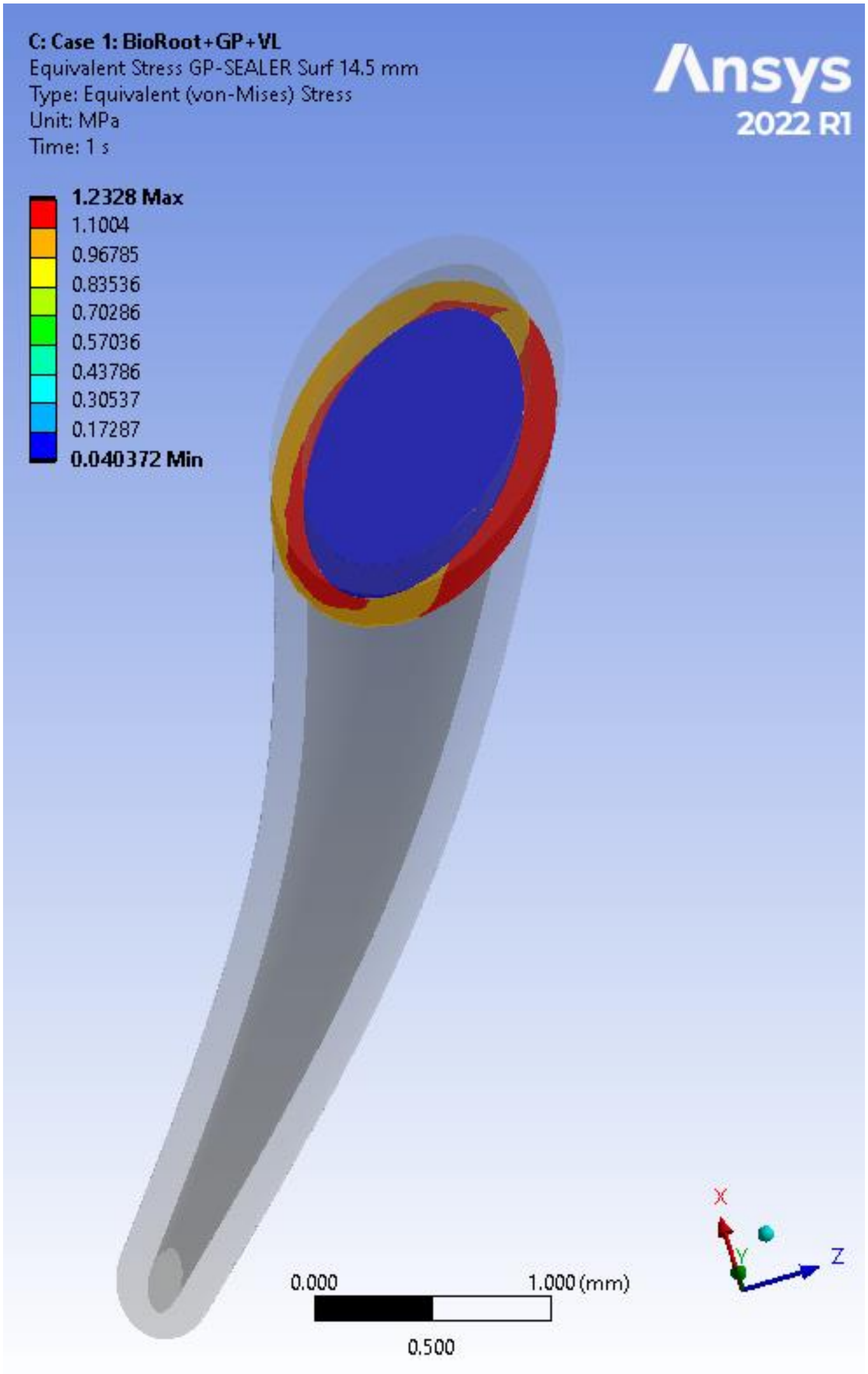
C: Case 1: BioRoot+GP+VL
Equivalent Stress GP-SEALER Surf 14.5 mm
Type: Equivalent (von-Mises) Stress
Unit: MPa
Time: 1 s
1.2328 Max
1.1004
0.96785
0.83536
0.70286
0.57036
0.43786
0.30537
0.17287
0.040372 Min
Ansys
2022 R1
0.000
1.000 (mm)
0.500
X
Y
Z

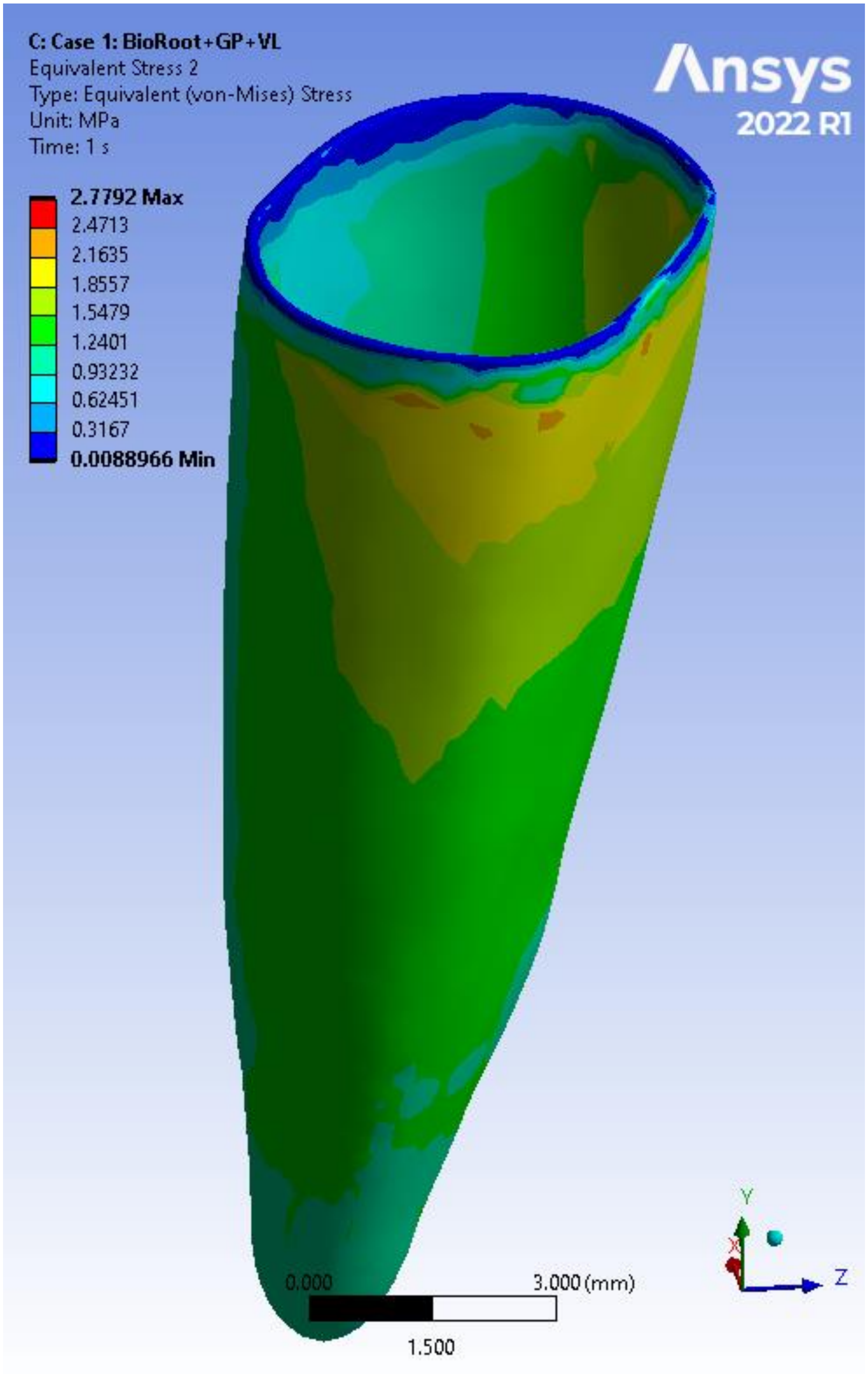
C: Case 1: BioRoot+GP+VL
Equivalent Stress 2
Type: Equivalent (von-Mises) Stress
Unit: MPa
Time: 1 s
2.7792 Max
2.4713
2.1635
1.8557
1.5479
1.2401
0.93232
0.62451
0.3167
0.0088966 Min
Ansys
2022 R1
0.000
3.000 (mm)
1.500
Y
X
Z

Case 2

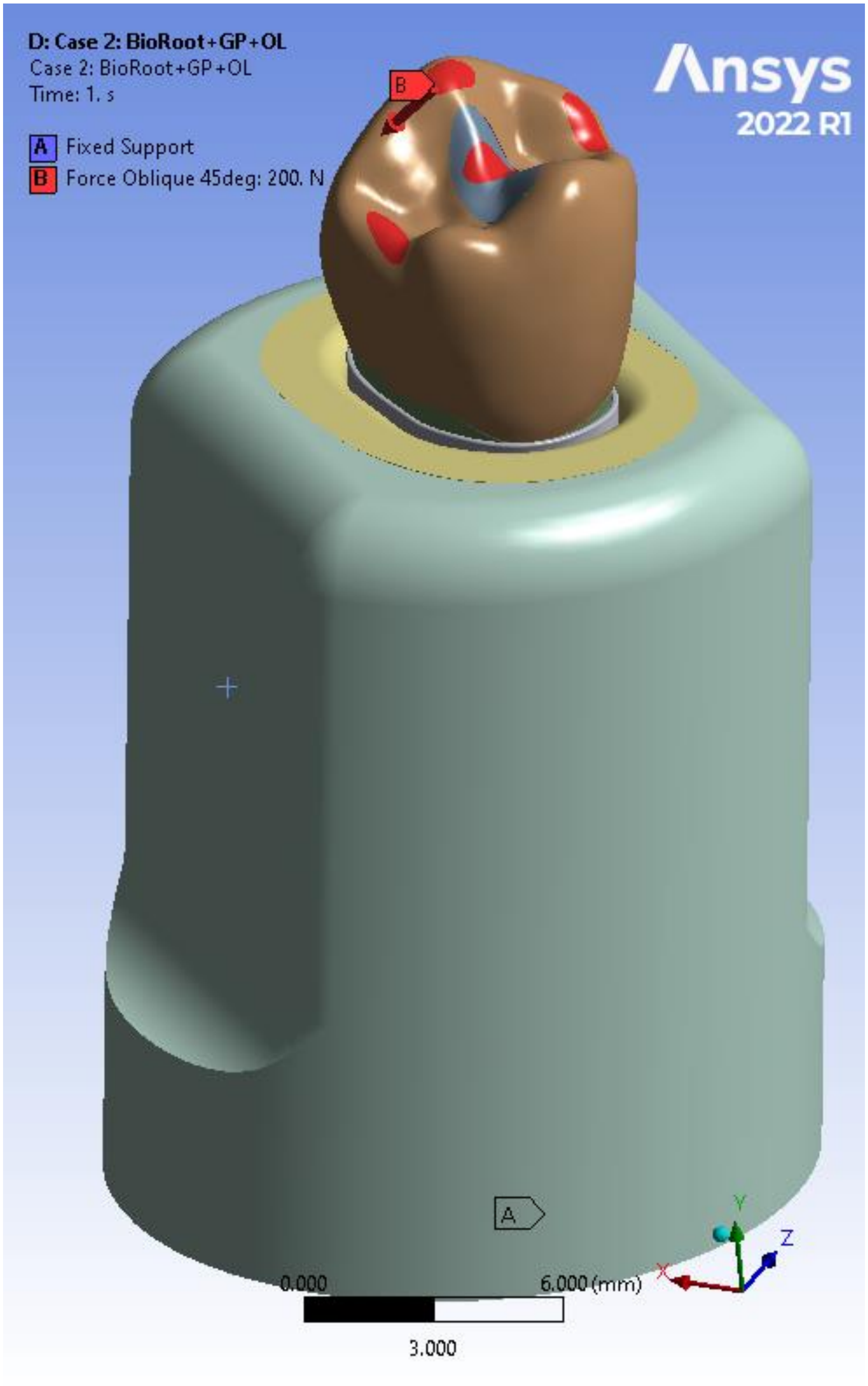
D: Case 2: BioRoot+GP+OL
Case 2: BioRoot+GP+OL
Time: 1. s
A Fixed Support
B Force Oblique 45deg: 200. N
Ansys
2022 R1
B
A
0.000
6.000 (mm)
3.000
Y
Z
X

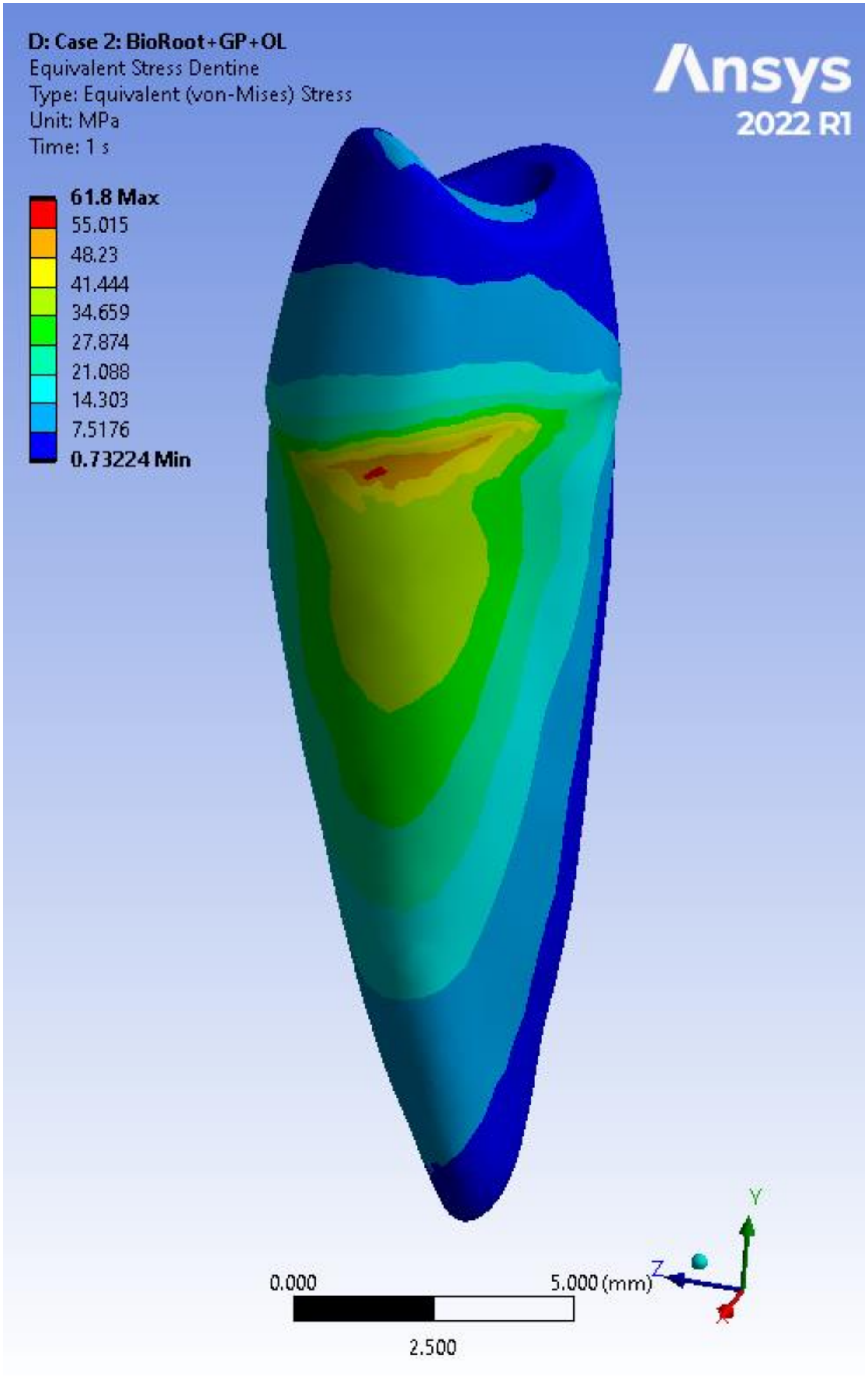

D: Case 2: BioRoot+GP+OL
Equivalent Stress Dentine
Type: Equivalent (von-Mises) Stress
Unit: MPa
Time: 1 s
61.8 Max
55.015
48.23
41.444
34.659
27.874
21.088
14.303
7.5176
0.73224 Min
Ansys
2022 R1
0.000
5.000 (mm)
2.500
Y
Z
X

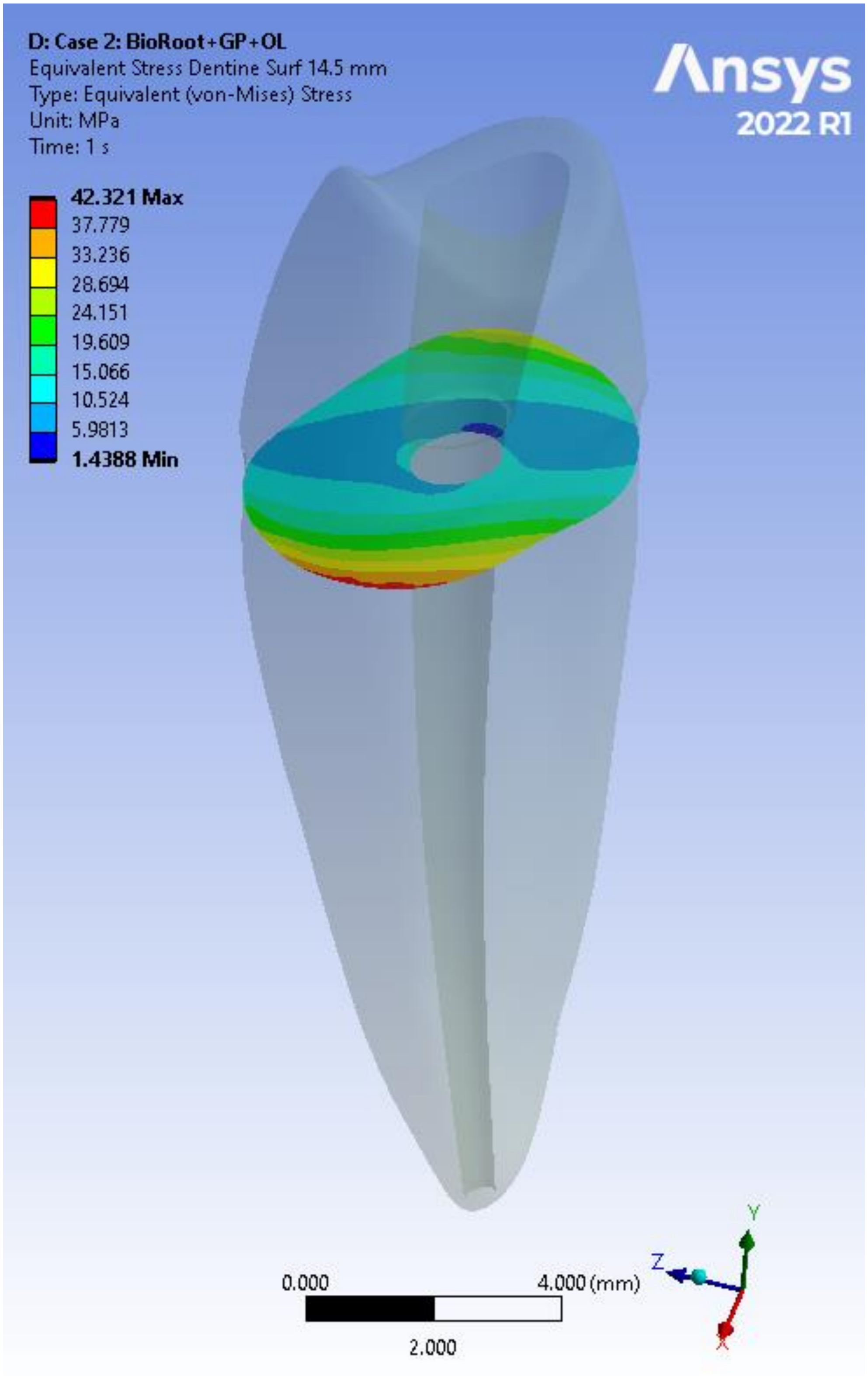

D: Case 2: BioRoot+GP+OL
Equivalent Stress Dentine Surf 14.5 mm
Type: Equivalent (von-Mises) Stress
Unit: MPa
Time: 1 s
42.321 Max
37.779
33.236
28.694
24.151
19.609
15.066
10.524
5.9813
1.4388 Min
Ansys
2022 R1
0.000
4.000 (mm)
2.000
Y
Z
X

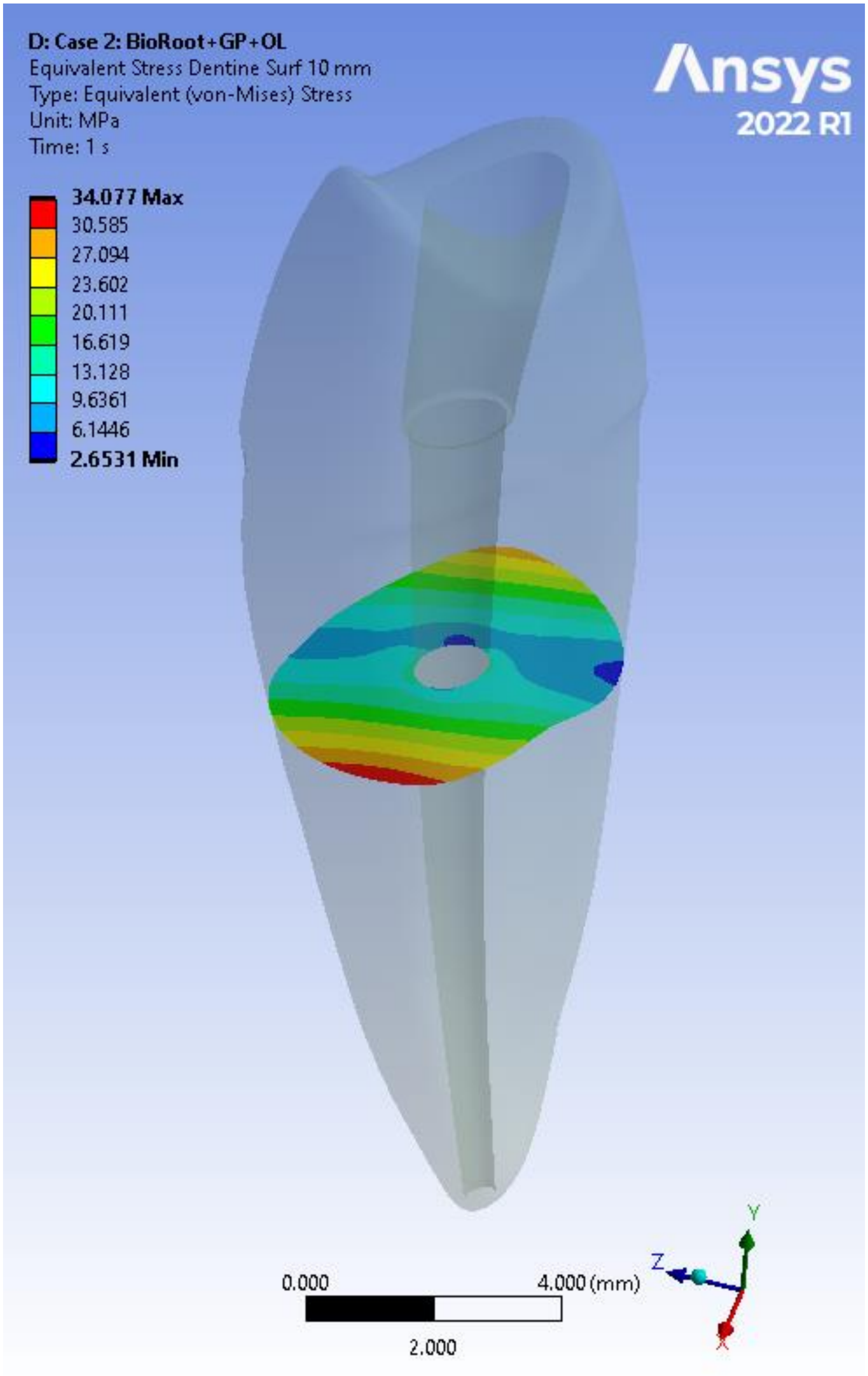

D: Case 2: BioRoot+GP+OL
Equivalent Stress Dentine Surf 10 mm
Type: Equivalent (von-Mises) Stress
Unit: MPa
Time: 1 s
34.077 Max
30.585
27.094
23.602
20.111
16.619
13.128
9.6361
6.1446
2.6531 Min
Ansys
2022 R1
0.000
4.000 (mm)
2.000
Y
Z
X

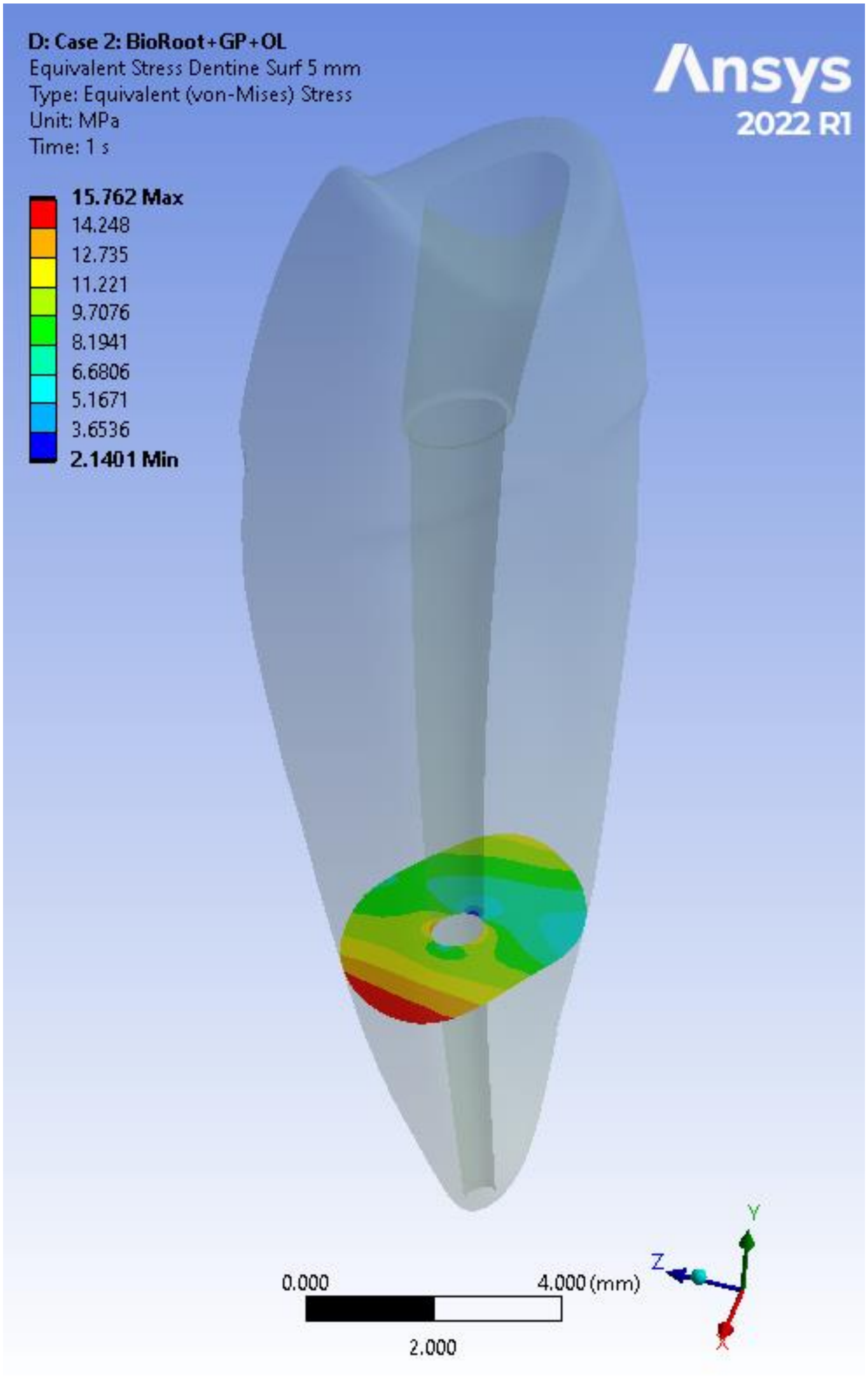
D: Case 2: BioRoot+GP+OL
Equivalent Stress Dentine Surf 5 mm
Type: Equivalent (von-Mises) Stress
Unit: MPa
Time: 1 s
15.762 Max
14.248
12.735
11.221
9.7076
8.1941
6.6806
5.1671
3.6536
2.1401 Min
Ansys
2022 R1
0.000
4.000 (mm)
2.000
Y
Z
X

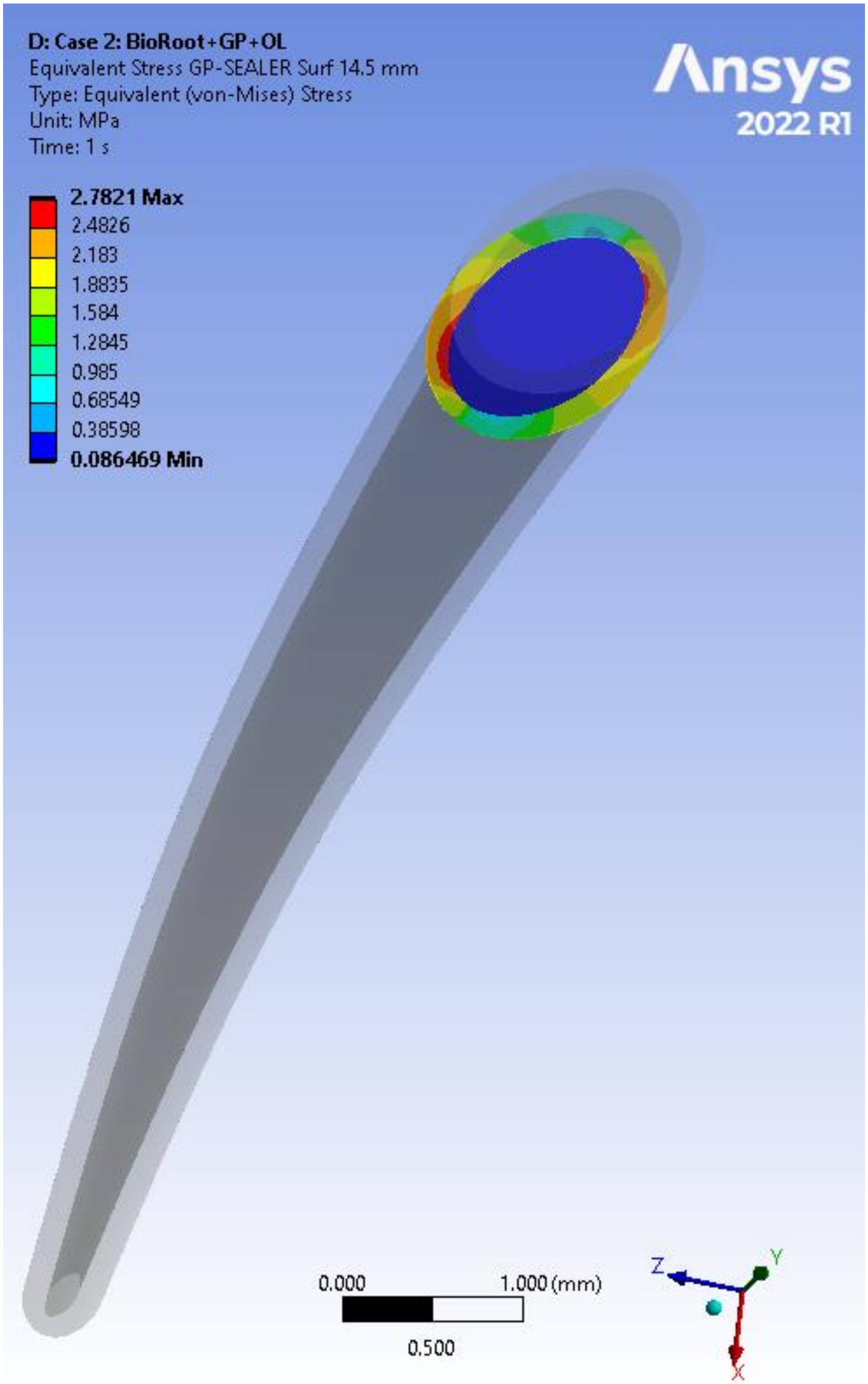
D: Case 2: BioRoot+GP+OL
Equivalent Stress GP-SEALER Surf 14.5 mm
Type: Equivalent (von-Mises) Stress
Unit: MPa
Time: 1 s
Ansys
2022 R1
2.7821 Max
2.4826
2.183
1.8835
1.584
1.2845
0.985
0.68549
0.38598
0.086469 Min
0.000
1.000 (mm)
0.500
Z
Y
X

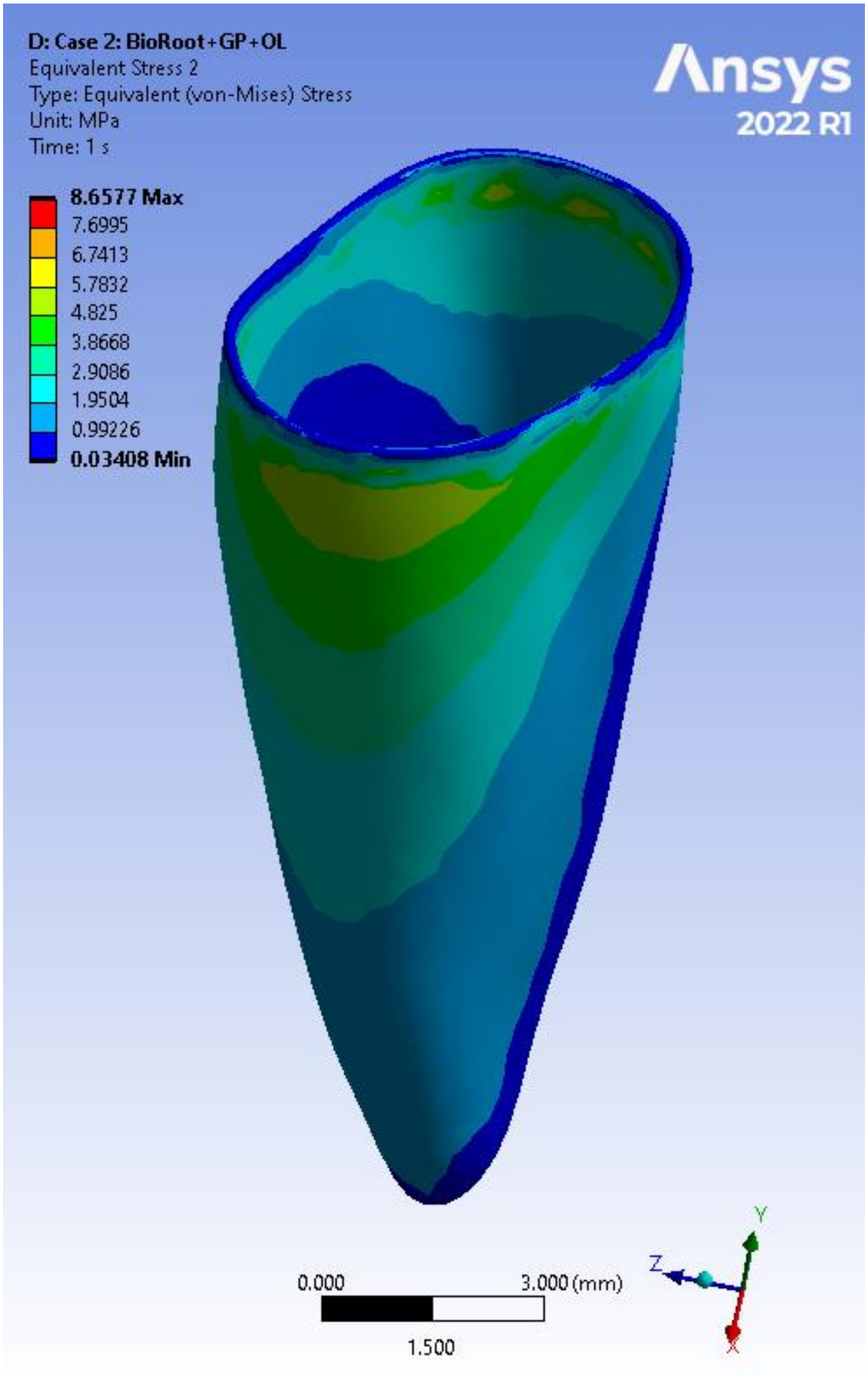

D: Case 2: BioRoot+GP+OL
Equivalent Stress 2
Type: Equivalent (von-Mises) Stress
Unit: MPa
Time: 1 s
Ansys
2022 R1
8.6577 Max
7.6995
6.7413
5.7832
4.825
3.8668
2.9086
1.9504
0.99226
0.03408 Min
0.000
3.000 (mm)
1.500
Y
Z
X

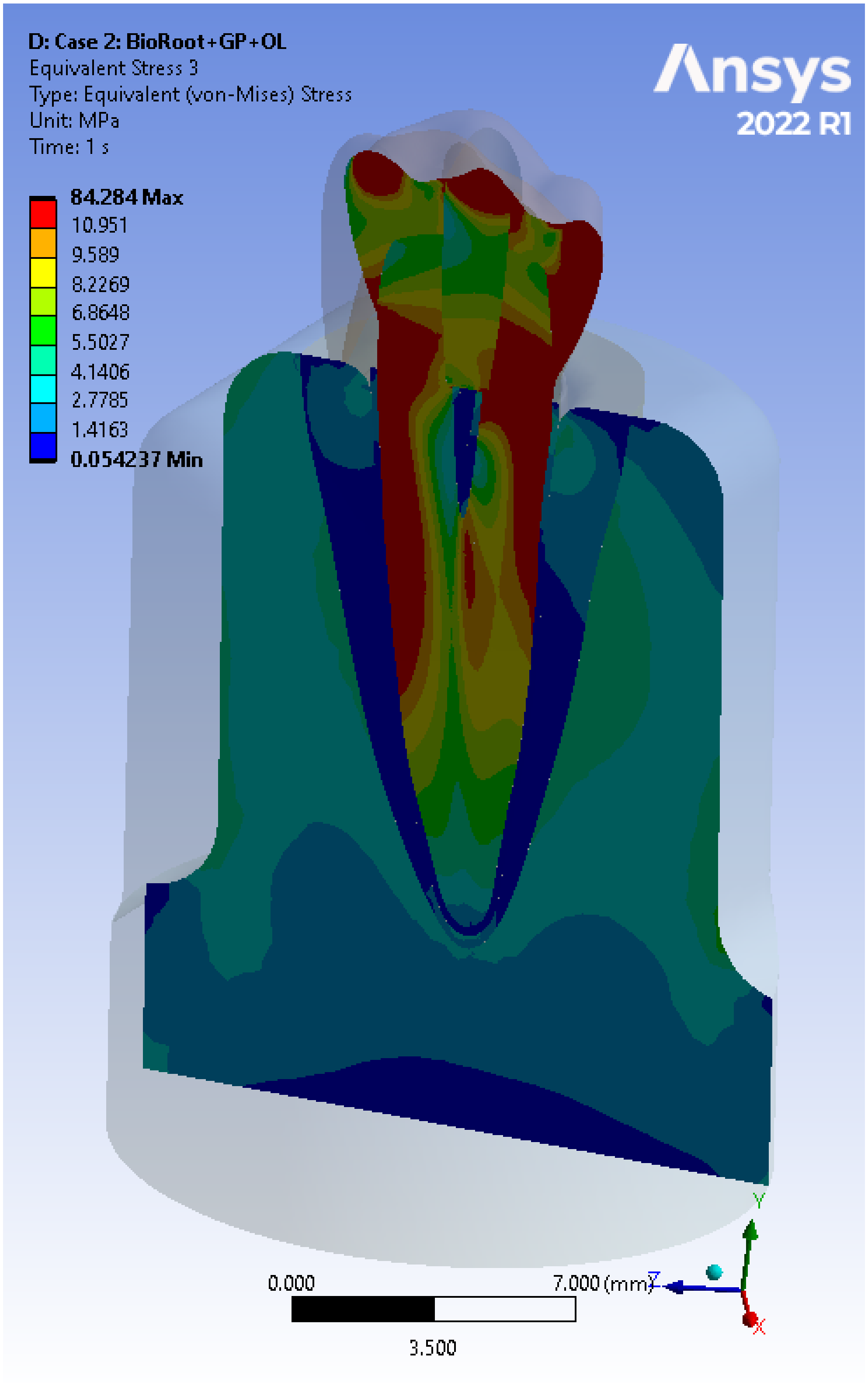
D: Case 2: BioRoot+GP+OL
Equivalent Stress 3
Type: Equivalent (von-Mises) Stress
Unit: MPa
Time: 1 s
84.284 Max
10.951
9.589
8.2269
6.8648
5.5027
4.1406
2.7785
1.4163
0.054237 Min
Ansys
2022 R1
0.000
7.000 (mm)
3.500
Y
Z
X

Case 3

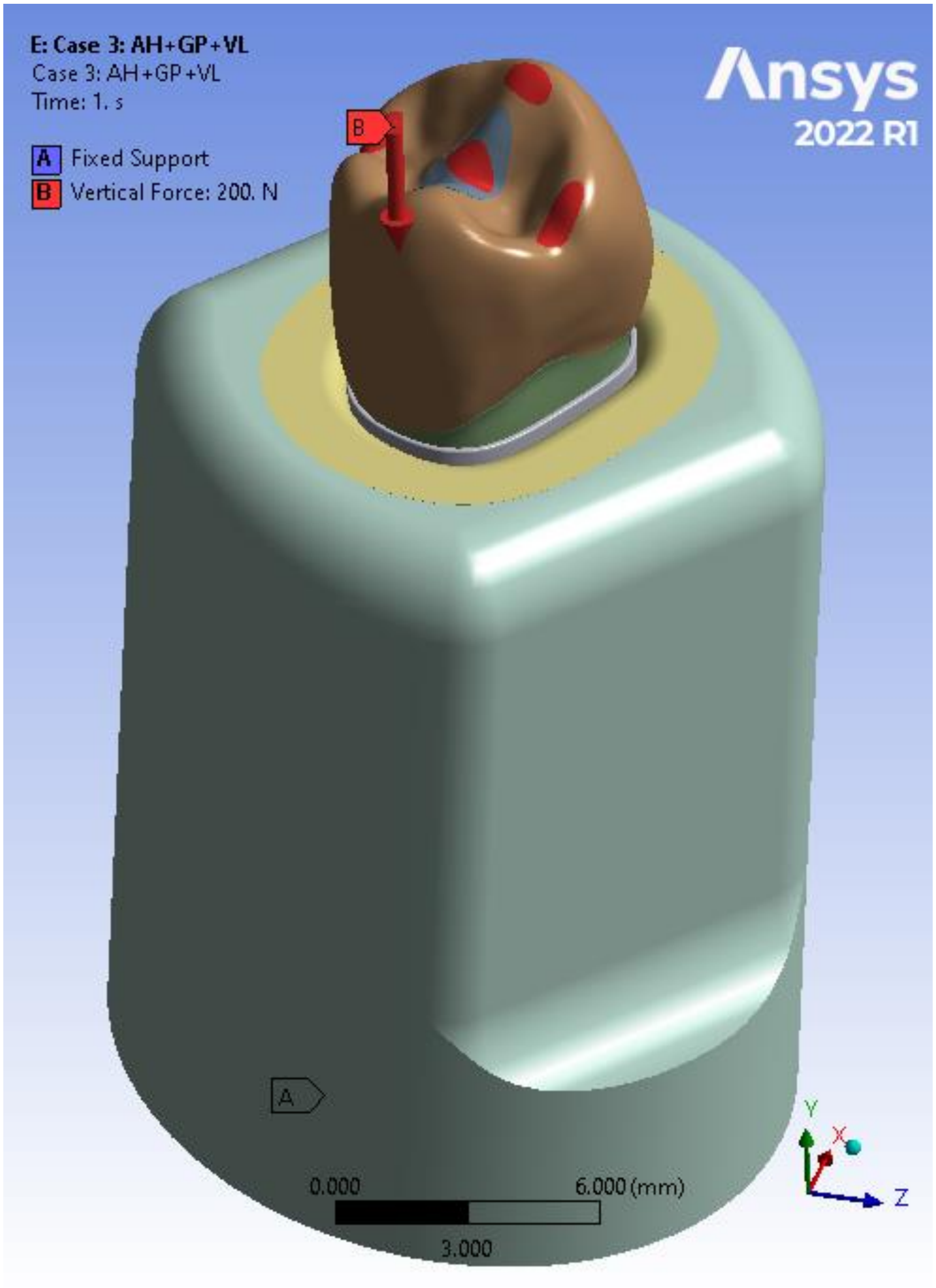

E: Case 3: AH+GP+VL
Case 3: AH+GP+VL
Time: 1. s
A Fixed Support
B Vertical Force: 200. N
Ansys
2022 R1
B
A
0.000
6.000 (mm)
3.000
Y
X
Z

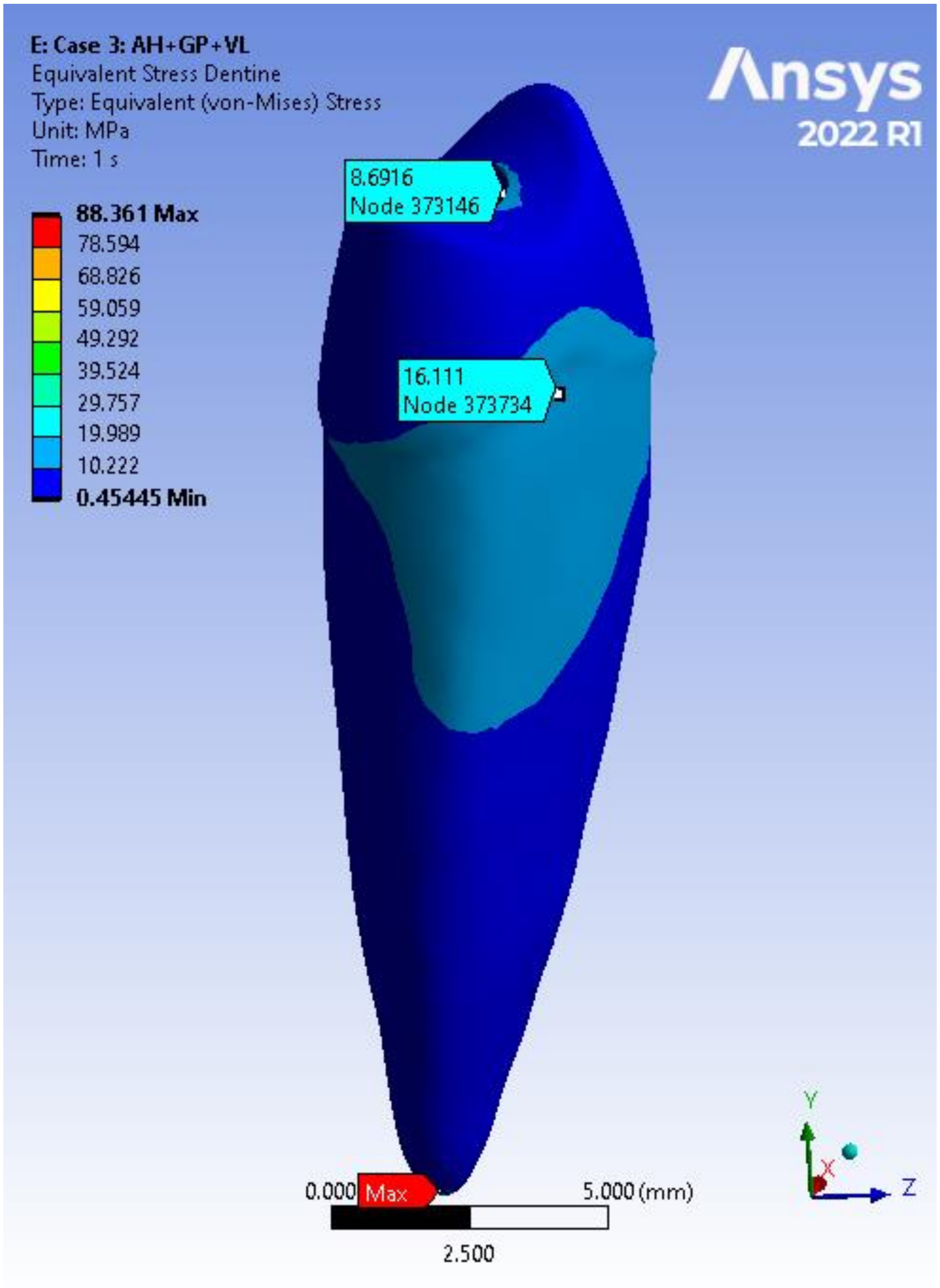
E: Case 3: AH+GP+VL
Equivalent Stress Dentine
Type: Equivalent (von-Mises) Stress
Unit: MPa
Time: 1 s
88.361 Max
78.594
68.826
59.059
49.292
39.524
29.757
19.989
10.222
0.45445 Min
Ansys
2022 R1
8.6916
Node 373146
16.111
Node 373734
Max
0.000
5.000 (mm)
2.500
Y
X
Z

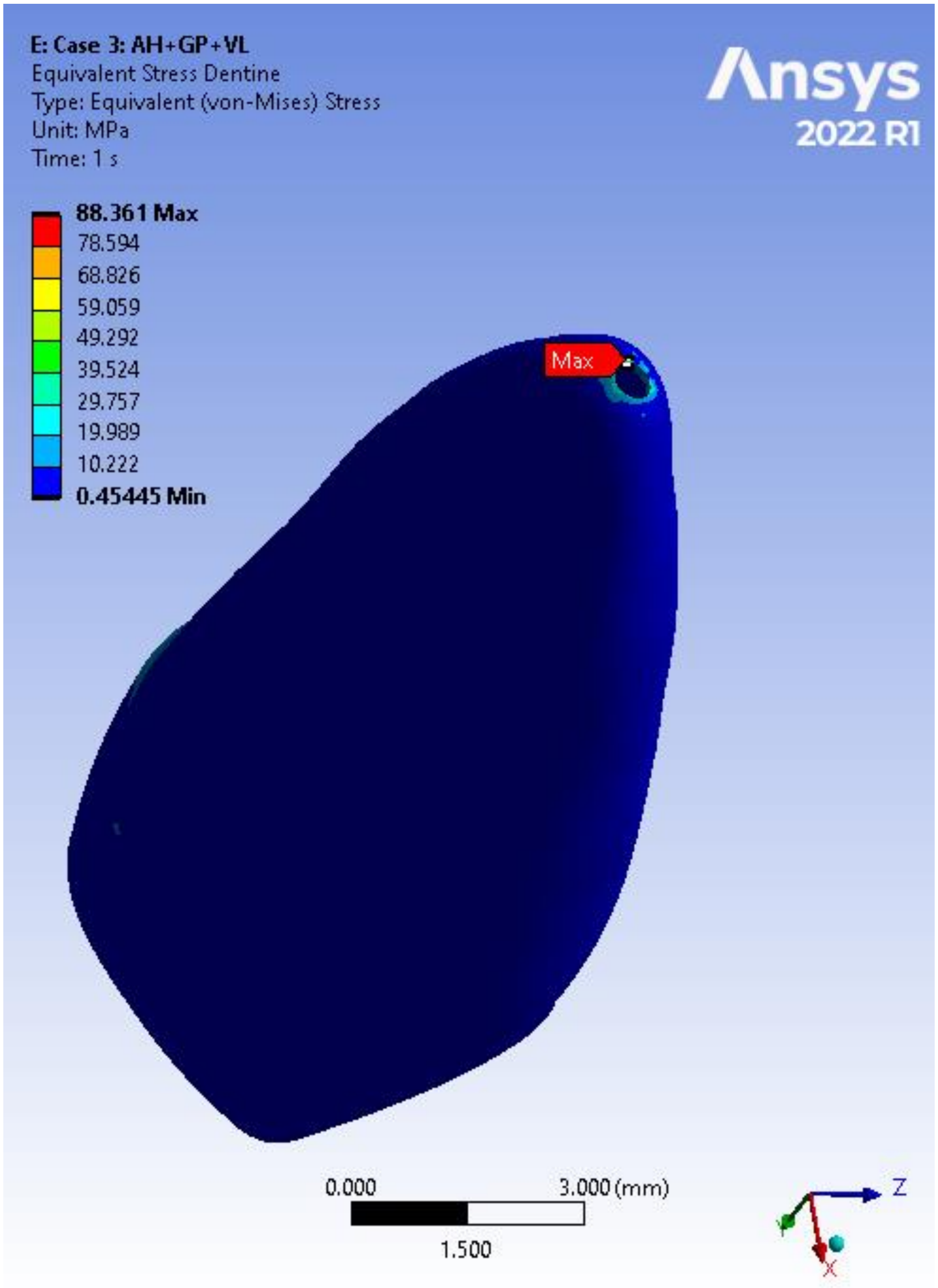
E: Case 3: AH+GP+VL
Equivalent Stress Dentine
Type: Equivalent (von-Mises) Stress
Unit: MPa
Time: 1 s
Ansys
2022 R1
88.361 Max
78.594
68.826
59.059
49.292
39.524
29.757
19.989
10.222
0.45445 Min
Max
0.000
3.000 (mm)
1.500
Z
Y
X

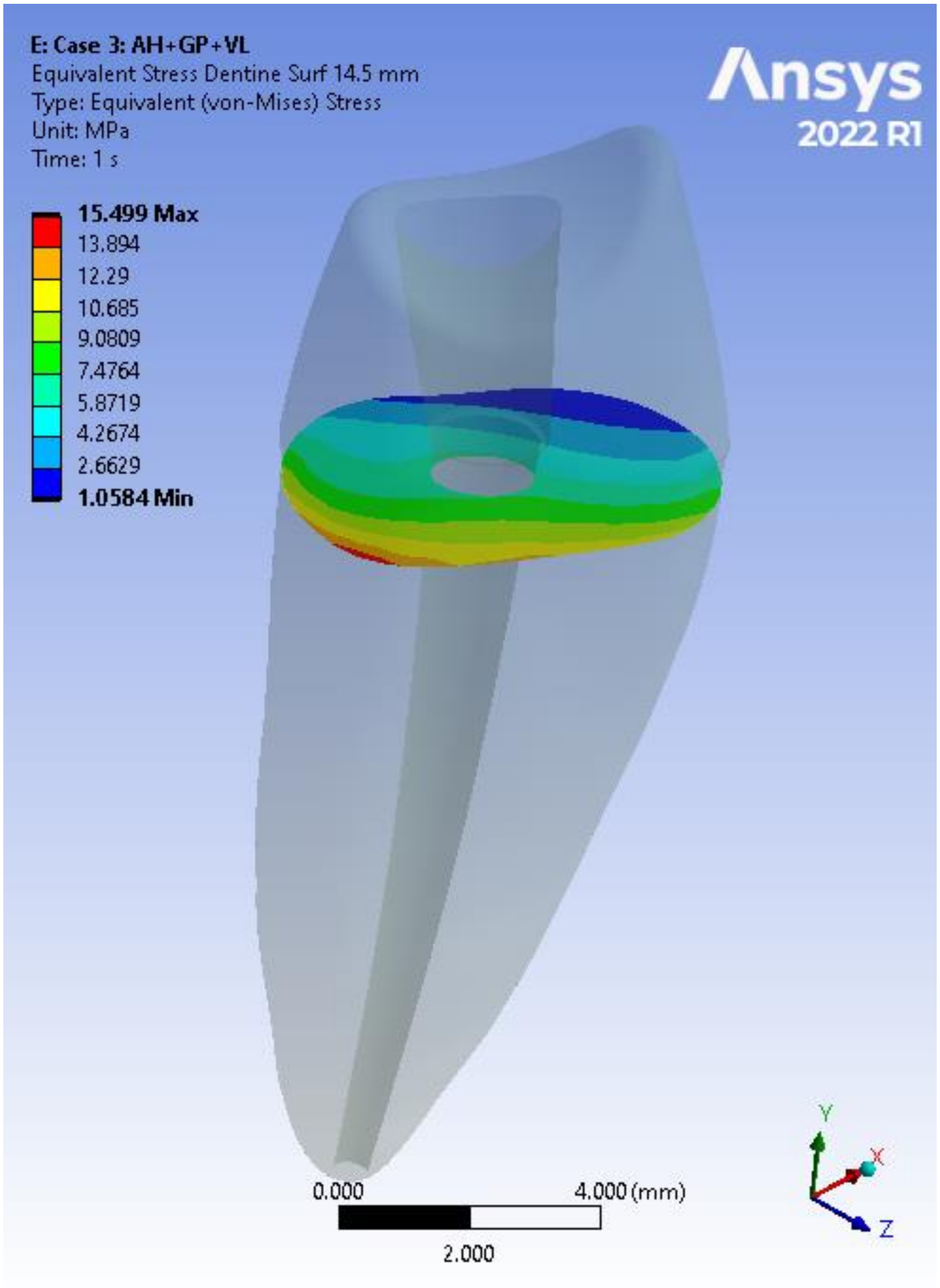
E: Case 3: AH+GP+VL
Equivalent Stress Dentine Surf 14.5 mm
Type: Equivalent (von-Mises) Stress
Unit: MPa
Time: 1 s
15.499 Max
13.894
12.29
10.685
9.0809
7.4764
5.8719
4.2674
2.6629
1.0584 Min
Ansys
2022 R1
0.000
4.000 (mm)
2.000
Y
X
Z

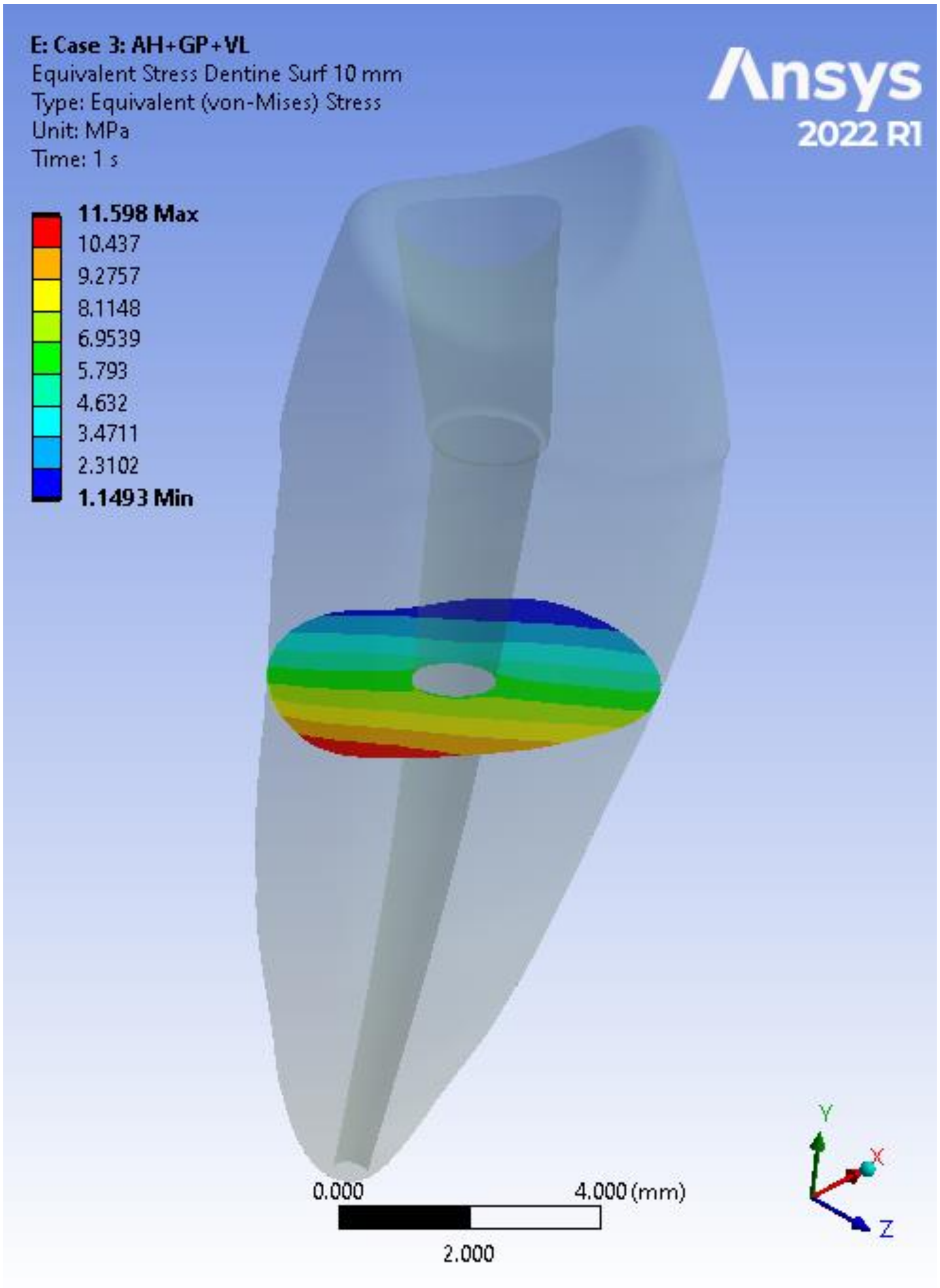
E: Case 3: AH+GP+VL
Equivalent Stress Dentine Surf 10 mm
Type: Equivalent (von-Mises) Stress
Unit: MPa
Time: 1 s
11.598 Max
10.437
9.2757
8.1148
6.9539
5.793
4.632
3.4711
2.3102
1.1493 Min
Ansys
2022 R1
0.000
4.000 (mm)
2.000
Y
X
Z

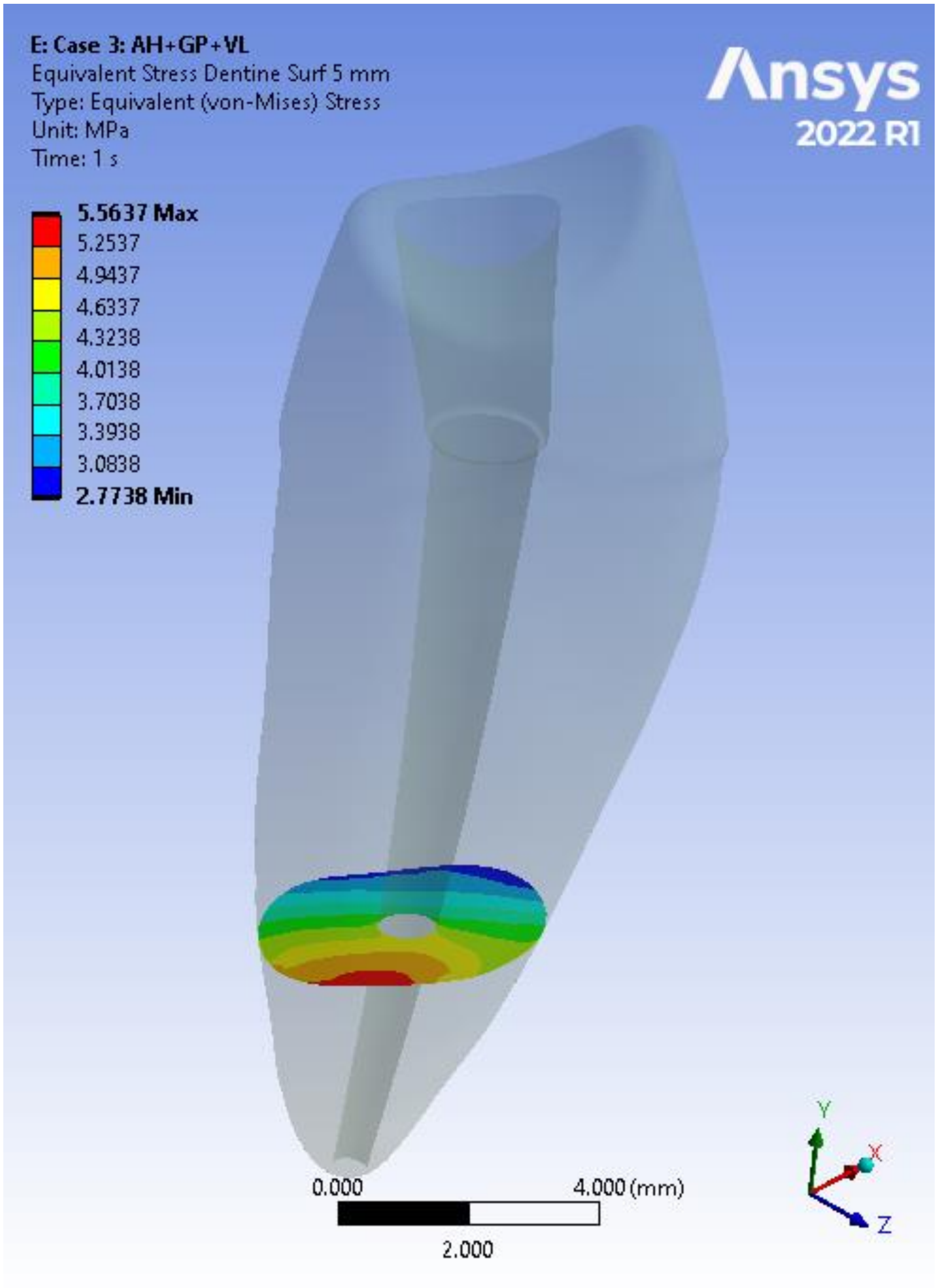
E: Case 3: AH+GP+VL
Equivalent Stress Dentine Surf 5 mm
Type: Equivalent (von-Mises) Stress
Unit: MPa
Time: 1 s
Ansys
2022 R1
5.5637 Max
5.2537
4.9437
4.6337
4.3238
4.0138
3.7038
3.3938
3.0838
2.7738 Min
0.000
4.000 (mm)
2.000
Y
X
Z

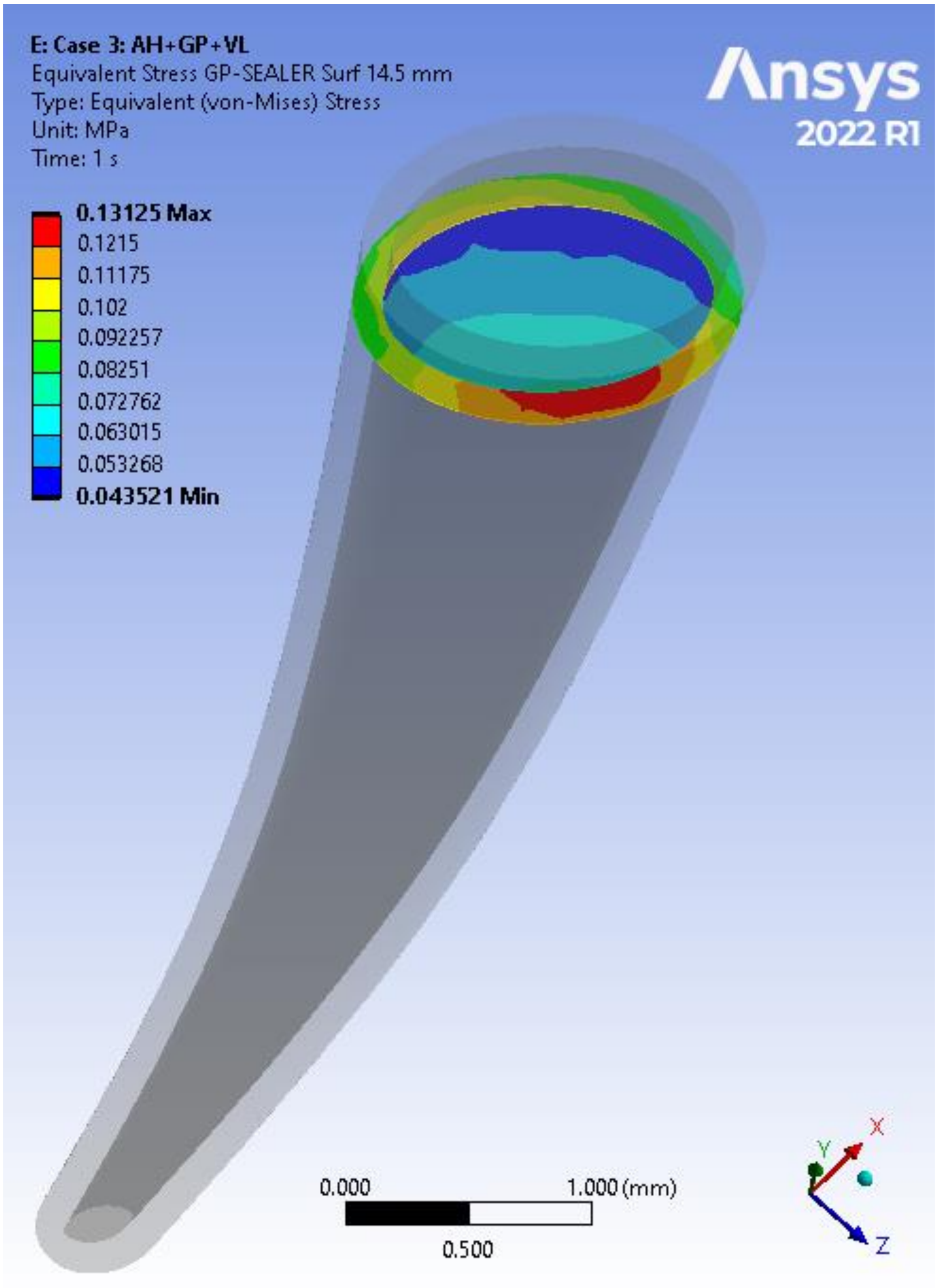
E: Case 3: AH+GP+VL
Equivalent Stress GP-SEALER Surf 14.5 mm
Type: Equivalent (von-Mises) Stress
Unit: MPa
Time: 1 s
0.13125 Max
0.1215
0.11175
0.102
0.092257
0.08251
0.072762
0.063015
0.053268
0.043521 Min
Ansys
2022 R1
0.000
1.000 (mm)
0.500
Y
X
Z

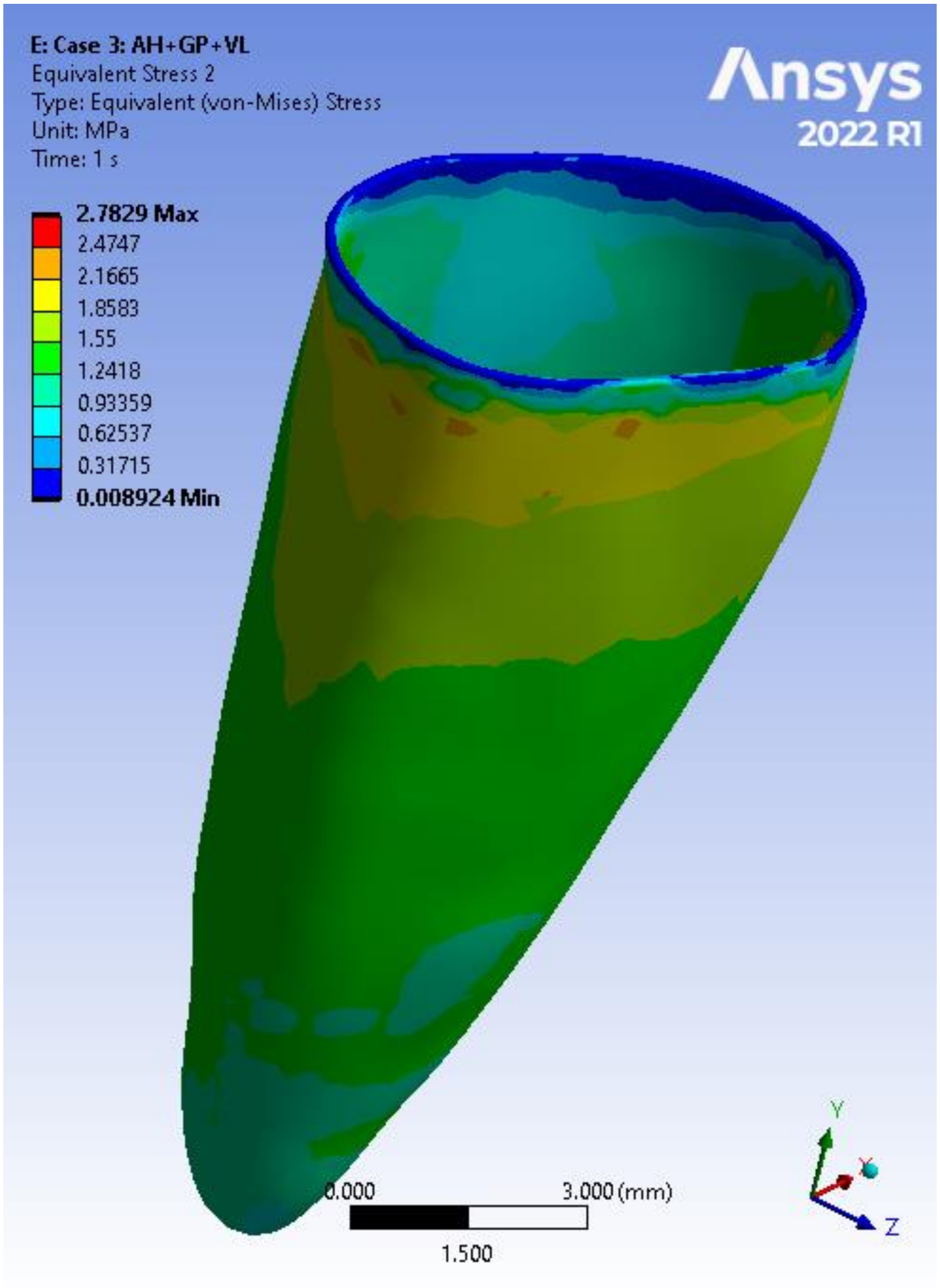
E: Case 3: AH+GP+VL
Equivalent Stress 2
Type: Equivalent (von-Mises) Stress
Unit: MPa
Time: 1 s
2.7829 Max
2.4747
2.1665
1.8583
1.55
1.2418
0.93359
0.62537
0.31715
0.008924 Min
Ansys
2022 R1
0.000
3.000 (mm)
1.500
Y
X
Z

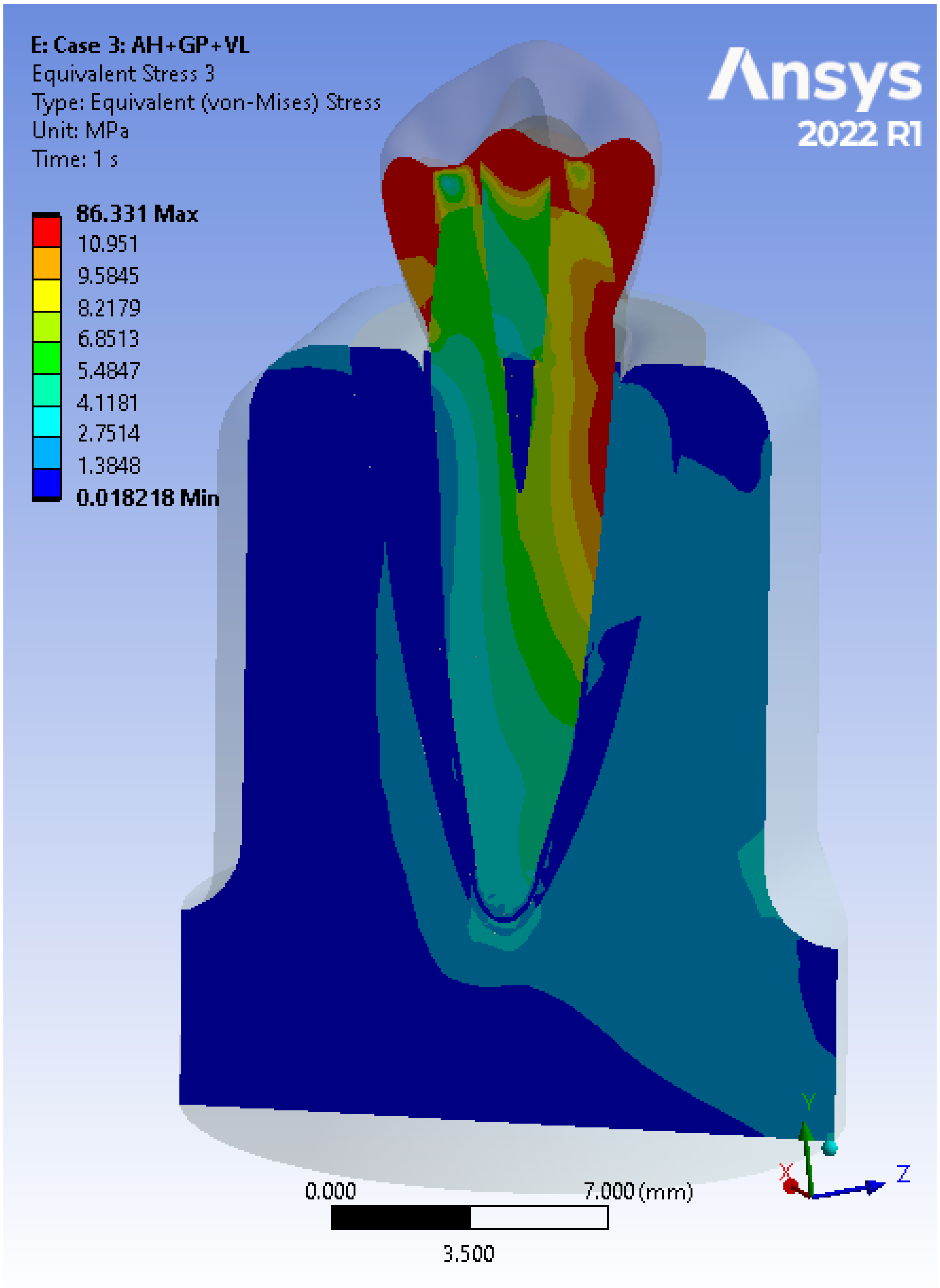
E: Case 3: AH+GP+VL
Equivalent Stress 3
Type: Equivalent (von-Mises) Stress
Unit: MPa
Time: 1 s
86.331 Max
10.951
9.5845
8.2179
6.8513
5.4847
4.1181
2.7514
1.3848
0.018218 Min
Ansys
2022 R1
0.000
7.000 (mm)
3.500
Y
X
Z

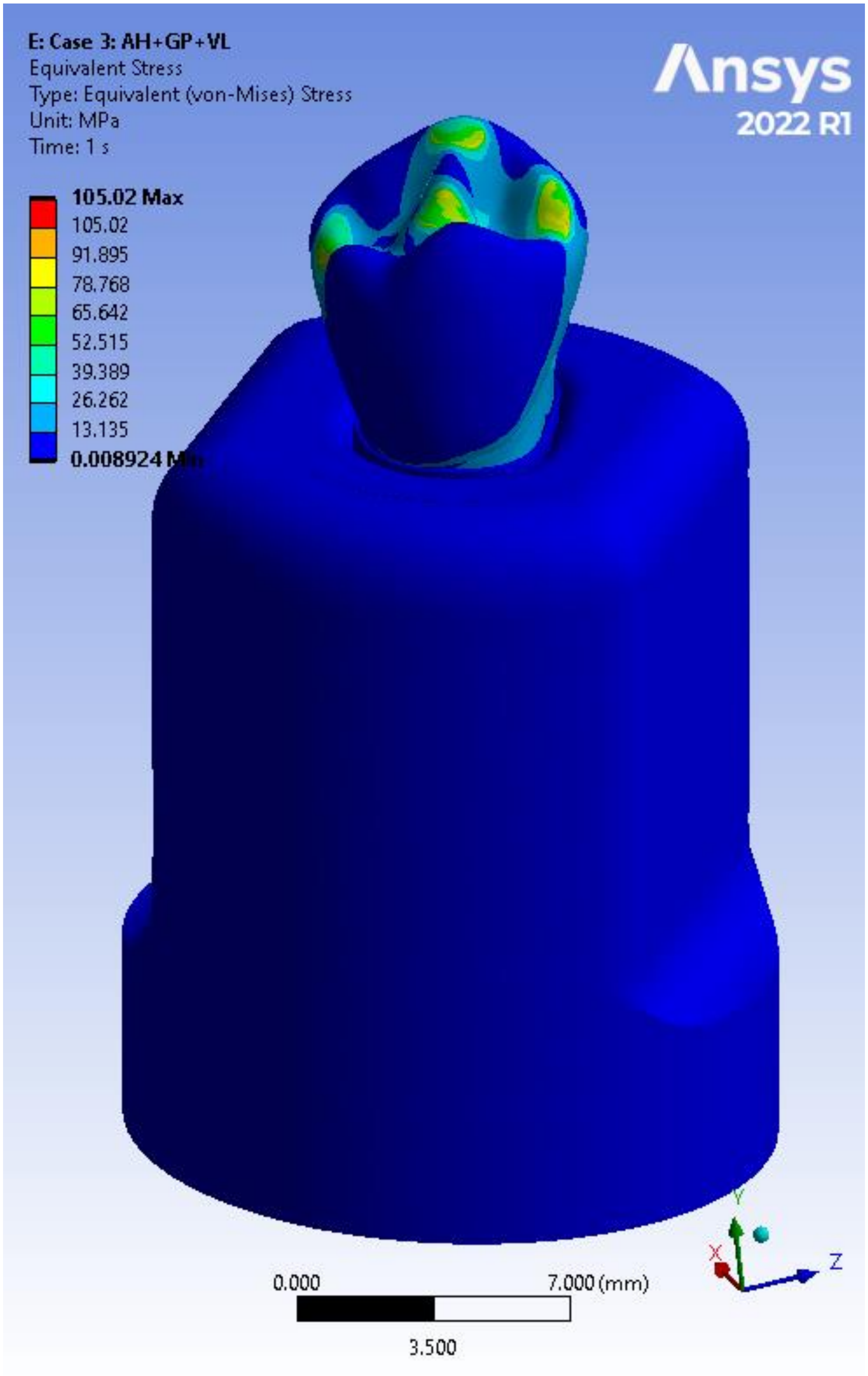
E: Case 3: AH+GP+VL
Equivalent Stress
Type: Equivalent (von-Mises) Stress
Unit: MPa
Time: 1 s
105.02 Max
105.02
91.895
78.768
65.642
52.515
39.389
26.262
13.135
0.008924 Min
Ansys
2022 R1
0.000
7.000 (mm)
3.500
Y
X
Z

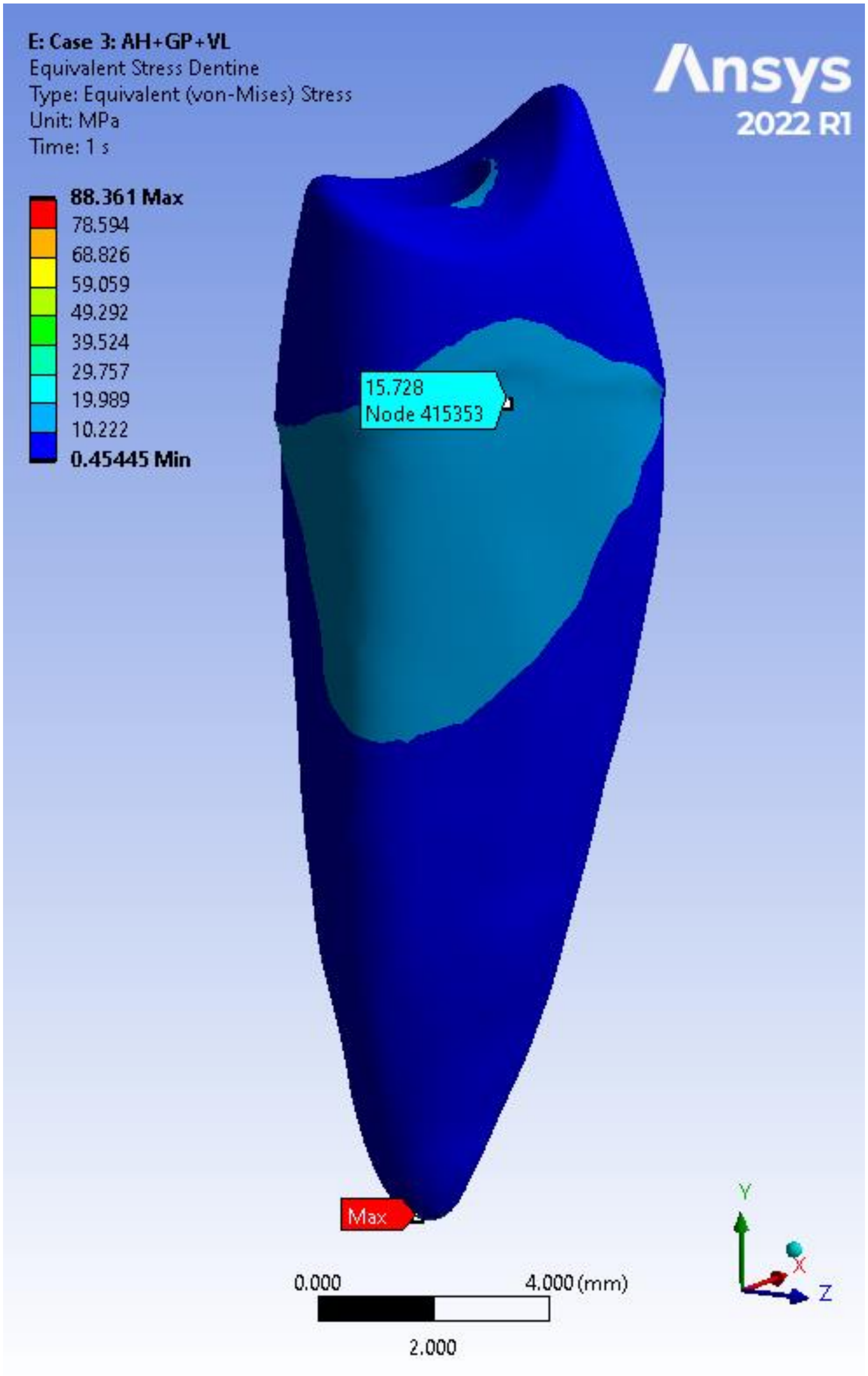

E: Case 3: AH+GP+VL
Equivalent Stress Dentine
Type: Equivalent (von-Mises) Stress
Unit: MPa
Time: 1 s
88.361 Max
78.594
68.826
59.059
49.292
39.524
29.757
19.989
10.222
0.45445 Min
Ansys
2022 R1
15.728
Node 415353
Max
0.000
4.000 (mm)
2.000
Y
X
Z

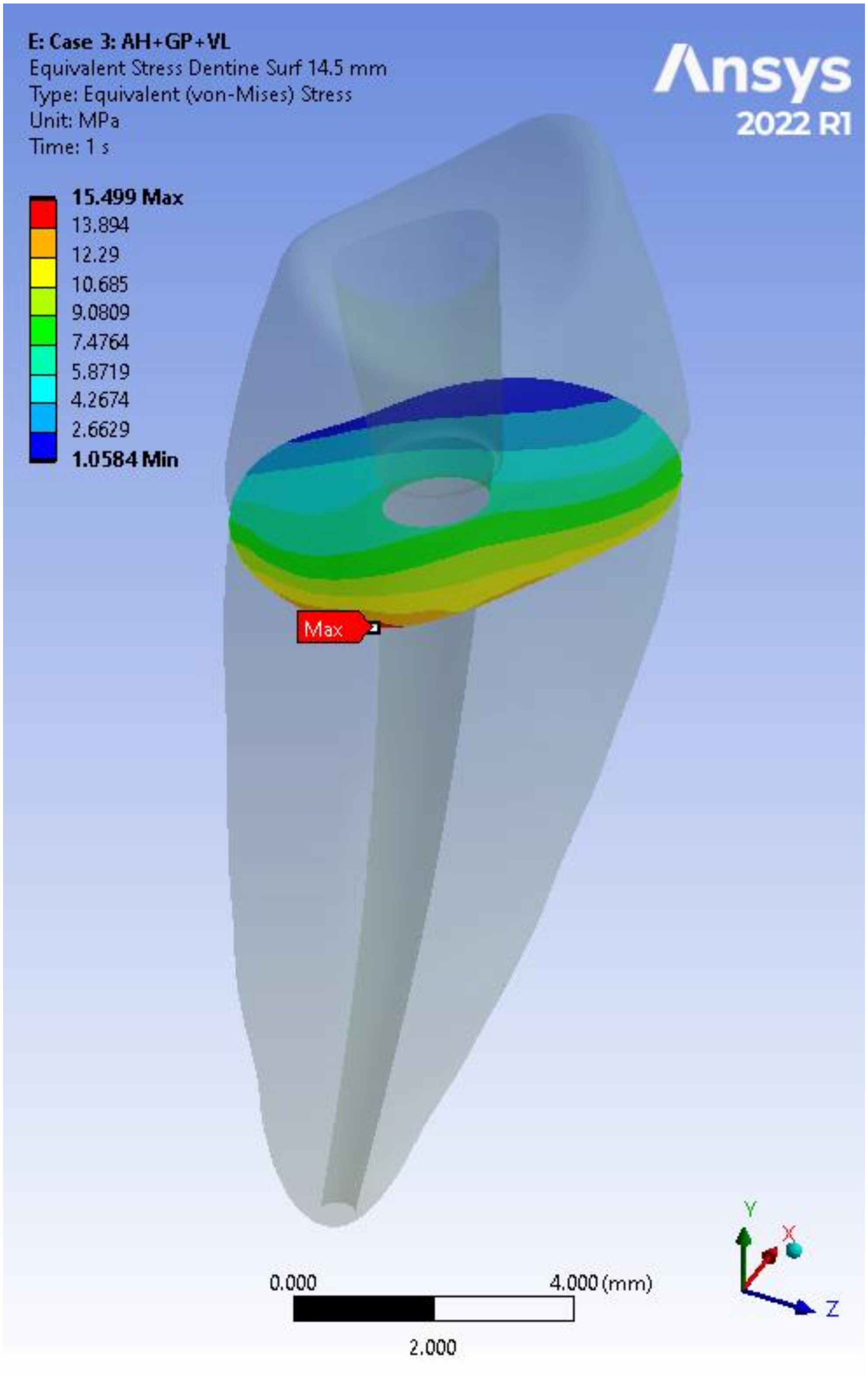
E: Case 3: AH+GP+VL
Equivalent Stress Dentine Surf 14.5 mm
Type: Equivalent (von-Mises) Stress
Unit: MPa
Time: 1 s
15.499 Max
13.894
12.29
10.685
9.0809
7.4764
5.8719
4.2674
2.6629
1.0584 Min
Ansys
2022 R1
Max
0.000
4.000 (mm)
2.000
Y
X
Z

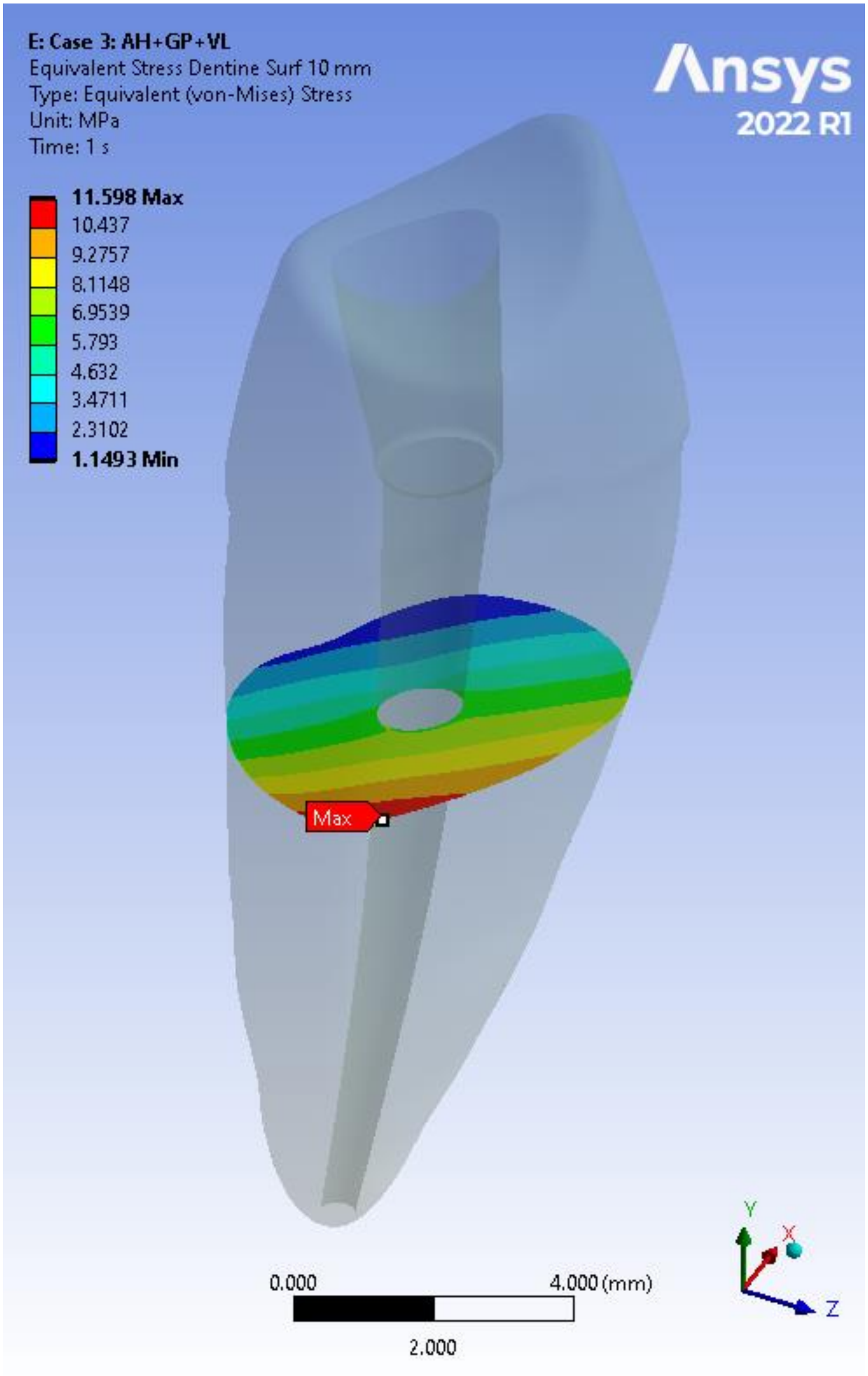

E: Case 3: AH+GP+VL
Equivalent Stress Dentine Surf 10 mm
Type: Equivalent (von-Mises) Stress
Unit: MPa
Time: 1 s
11.598 Max
10.437
9.2757
8.1148
6.9539
5.793
4.632
3.4711
2.3102
1.1493 Min
Ansys
2022 R1
Max
0.000
4.000 (mm)
2.000
Y
X
Z

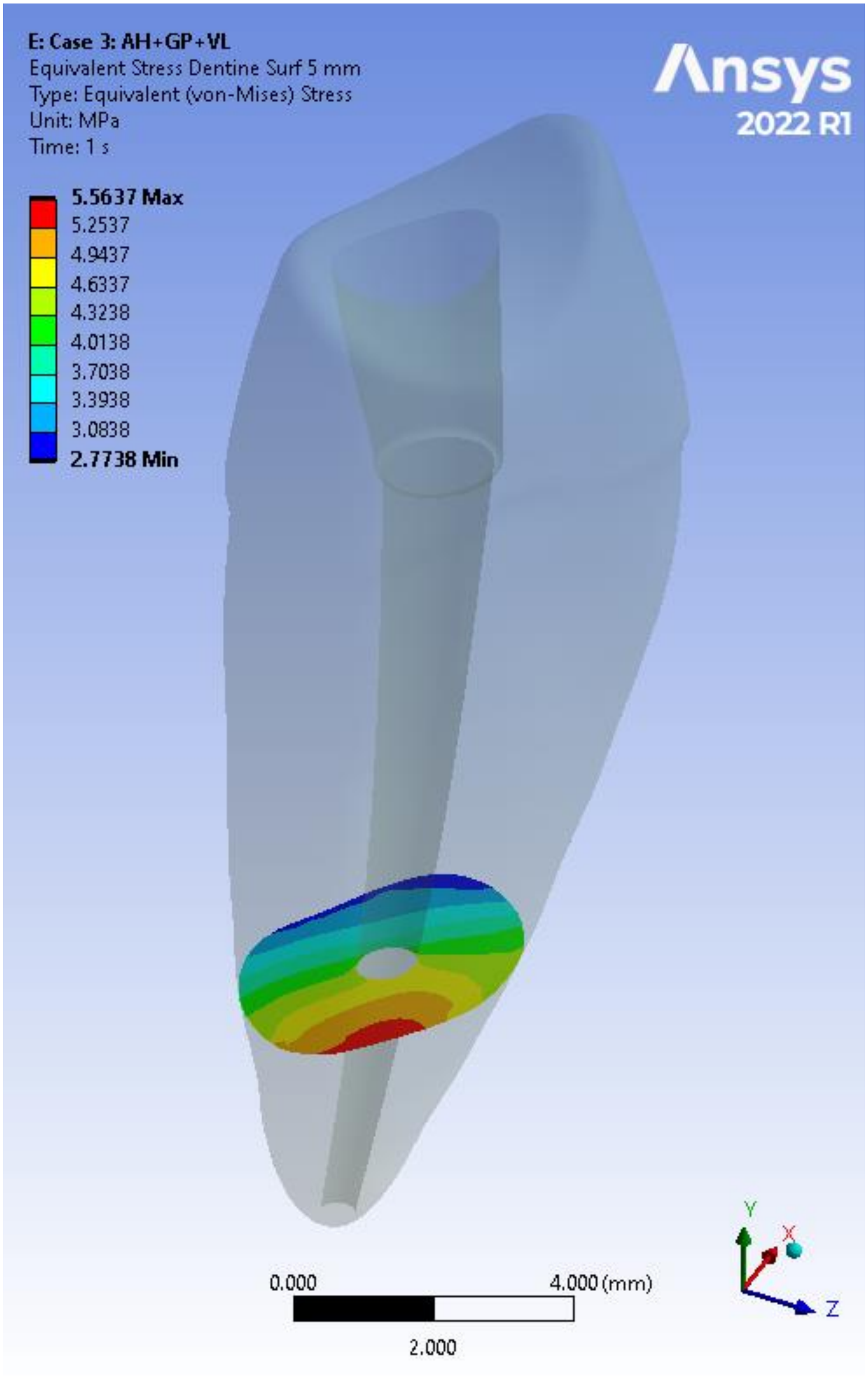

E: Case 3: AH+GP+VL
Equivalent Stress Dentine Surf 5 mm
Type: Equivalent (von-Mises) Stress
Unit: MPa
Time: 1 s
5.5637 Max
5.2537
4.9437
4.6337
4.3238
4.0138
3.7038
3.3938
3.0838
2.7738 Min
Ansys
2022 R1
0.000
4.000 (mm)
2.000
Y
X
Z

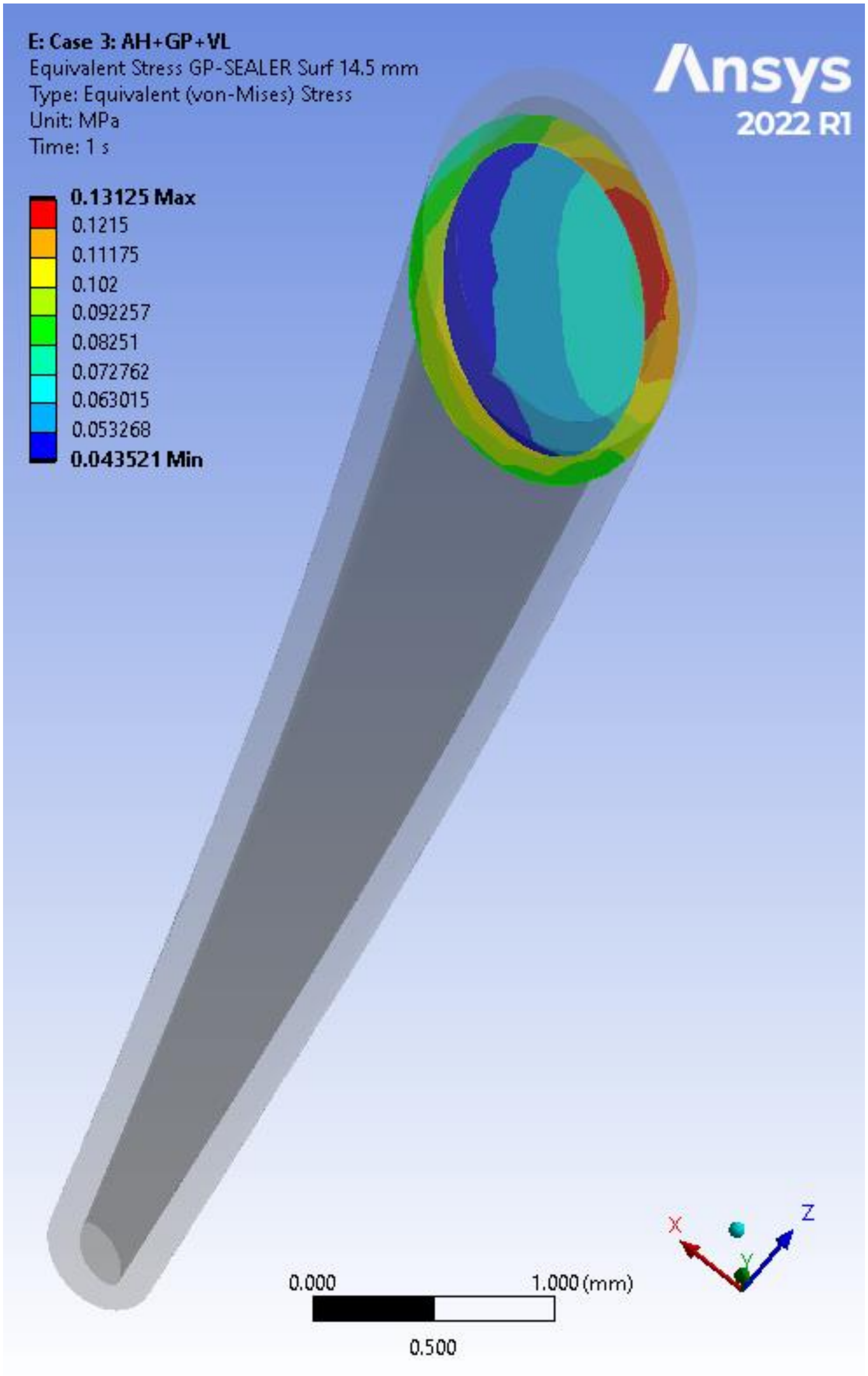

E: Case 3: AH+GP+VL
Equivalent Stress GP-SEALER Surf 14.5 mm
Type: Equivalent (von-Mises) Stress
Unit: MPa
Time: 1 s
0.13125 Max
0.1215
0.11175
0.102
0.092257
0.08251
0.072762
0.063015
0.053268
0.043521 Min
Ansys
2022 R1
0.000
1.000 (mm)
0.500
X
Y
Z

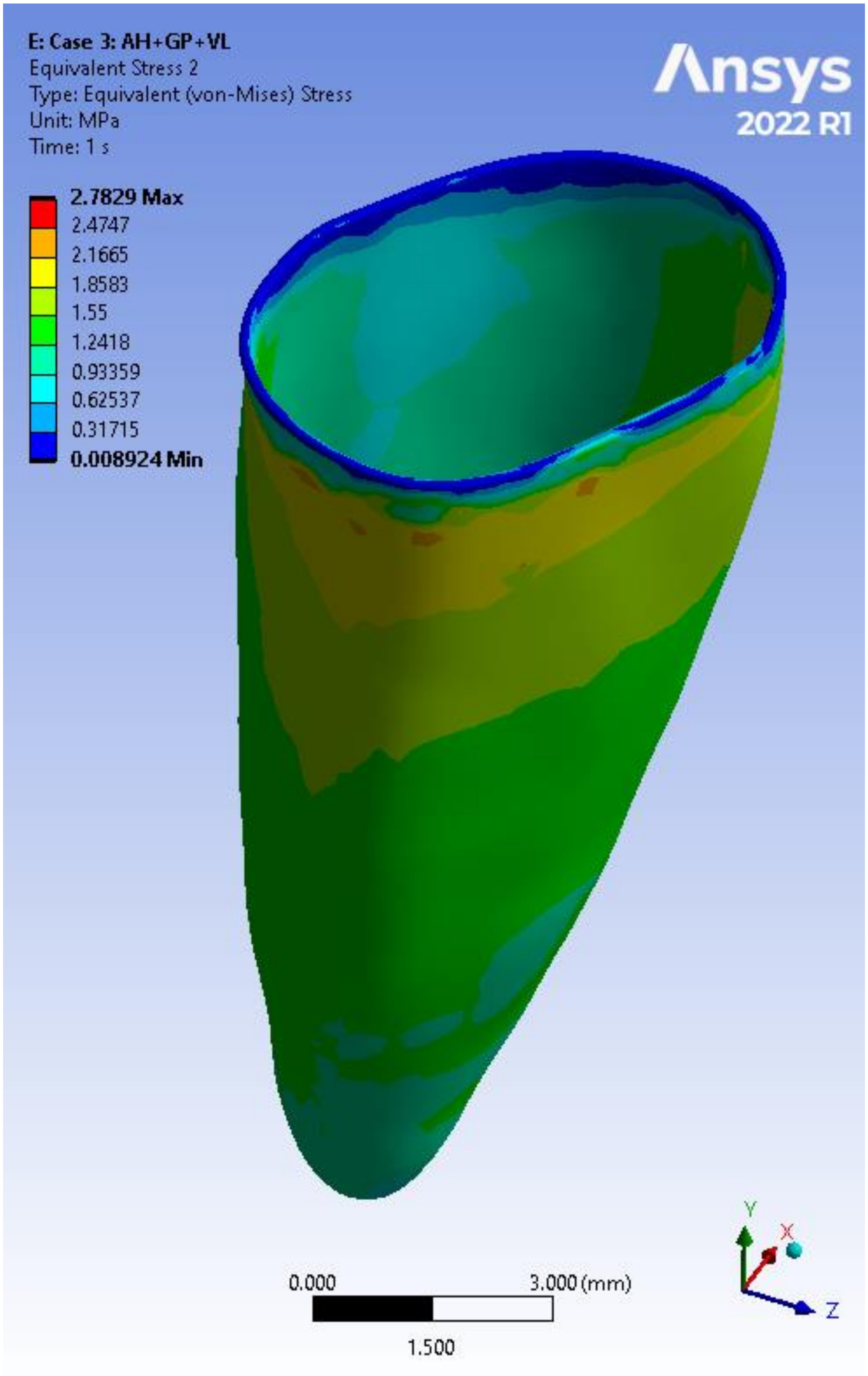
E: Case 3: AH+GP+VL
Equivalent Stress 2
Type: Equivalent (von-Mises) Stress
Unit: MPa
Time: 1 s
2.7829 Max
2.4747
2.1665
1.8583
1.55
1.2418
0.93359
0.62537
0.31715
0.008924 Min
Ansys
2022 R1
0.000
3.000 (mm)
1.500
Y
X
Z

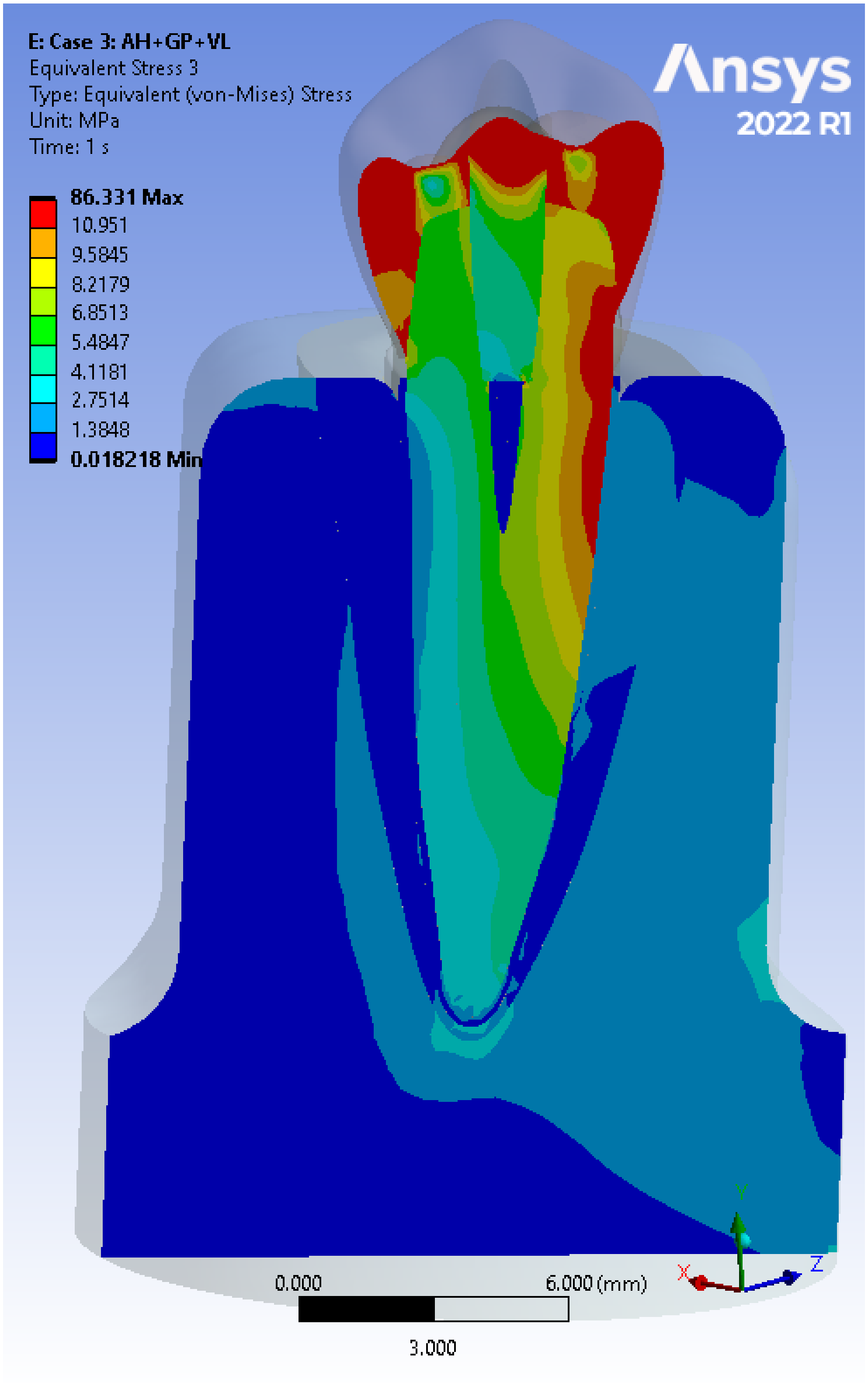

E: Case 3: AH+GP+VL
Equivalent Stress 3
Type: Equivalent (von-Mises) Stress
Unit: MPa
Time: 1 s
86.331 Max
10.951
9.5845
8.2179
6.8513
5.4847
4.1181
2.7514
1.3848
0.018218 Min
Ansys
2022 R1
0.000
6.000 (mm)
3.000
Y
X
Z

Case 4

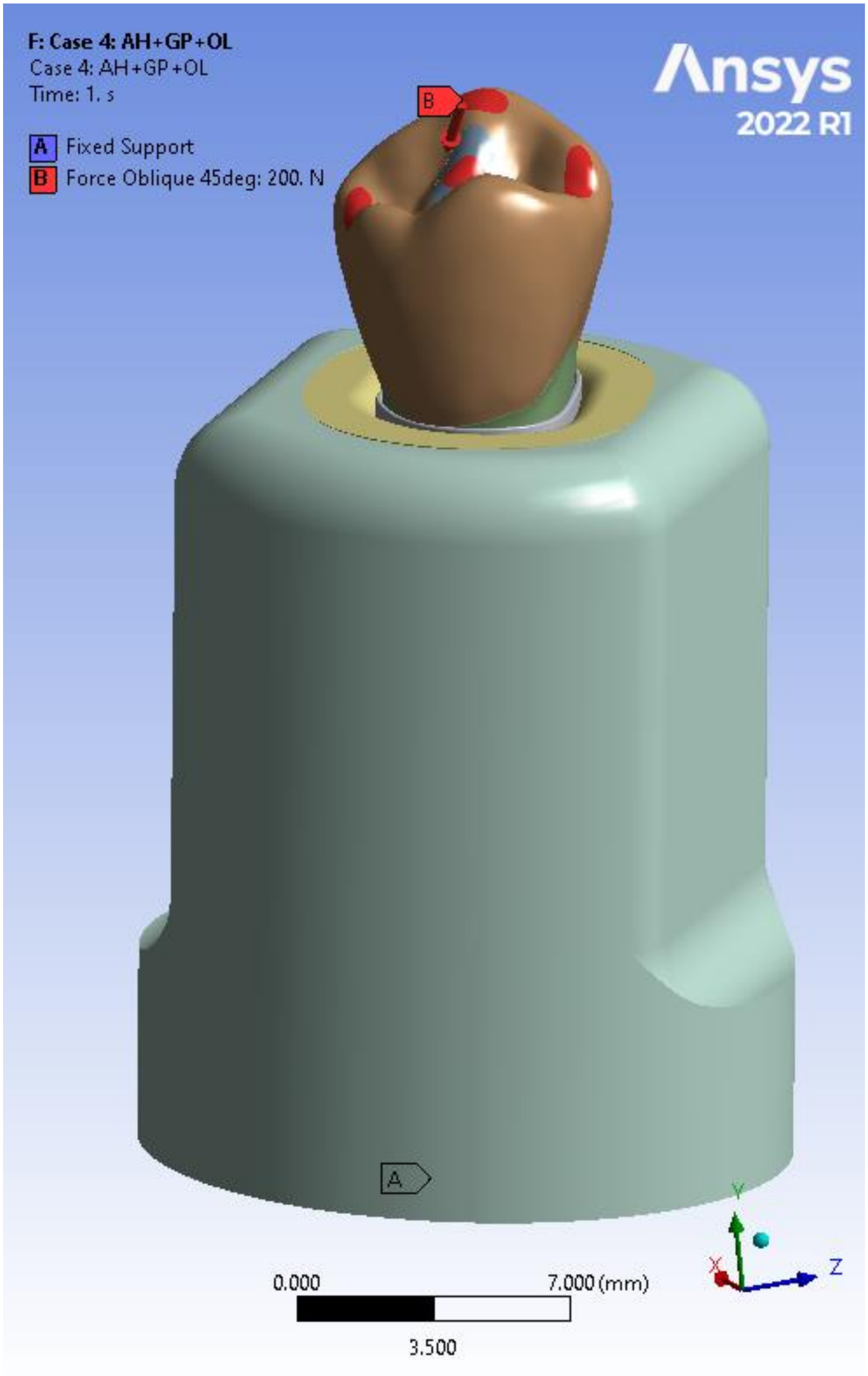

F: Case 4: AH+GP+OL
Case 4: AH+GP+OL
Time: 1. s
A Fixed Support
B Force Oblique 45deg: 200. N
Ansys
2022 R1
B
A
0.000
7.000 (mm)
3.500
Y
X
Z

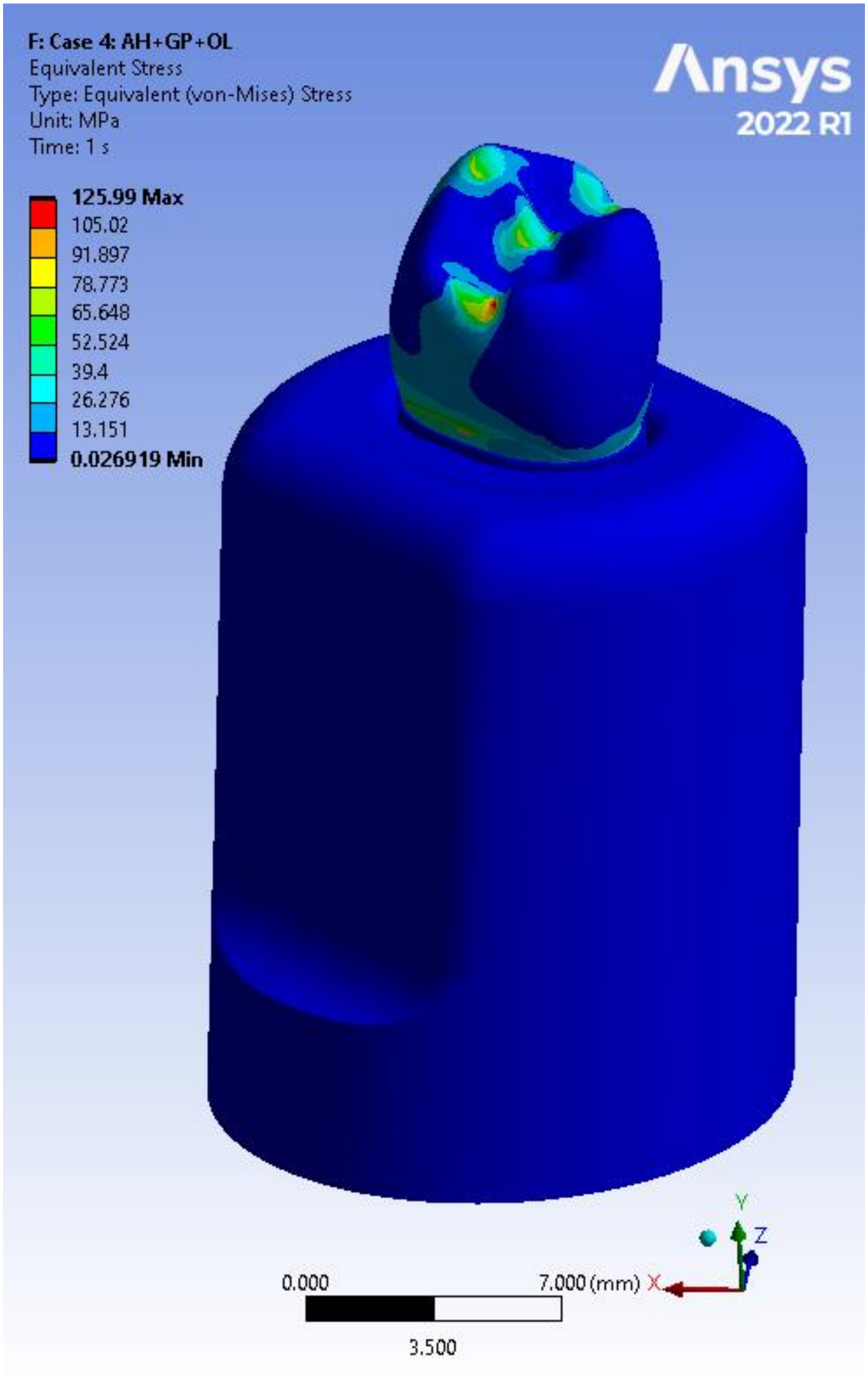
F: Case 4: AH+GP+OL
Equivalent Stress
Type: Equivalent (von-Mises) Stress
Unit: MPa
Time: 1 s
125.99 Max
105.02
91.897
78.773
65.648
52.524
39.4
26.276
13.151
0.026919 Min
Ansys
2022 R1
0.000
7.000 (mm)
3.500
Y
Z
X

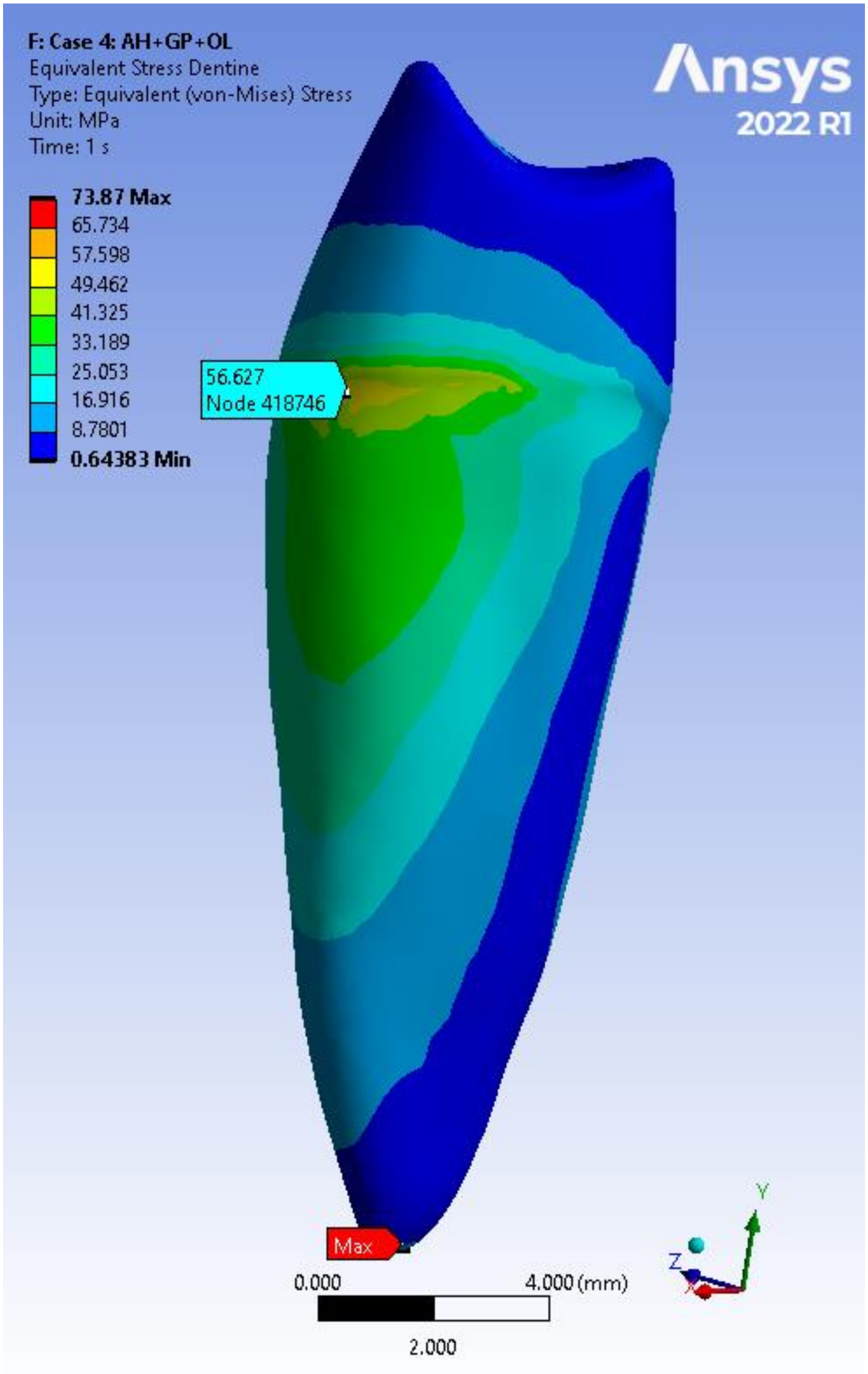
F: Case 4: AH+GP+OL
Equivalent Stress Dentine
Type: Equivalent (von-Mises) Stress
Unit: MPa
Time: 1 s
73.87 Max
65.734
57.598
49.462
41.325
33.189
25.053
16.916
8.7801
0.64383 Min
56.627
Node 418746
Ansys
2022 R1
Max
0.000
4.000 (mm)
2.000
Y
Z
X

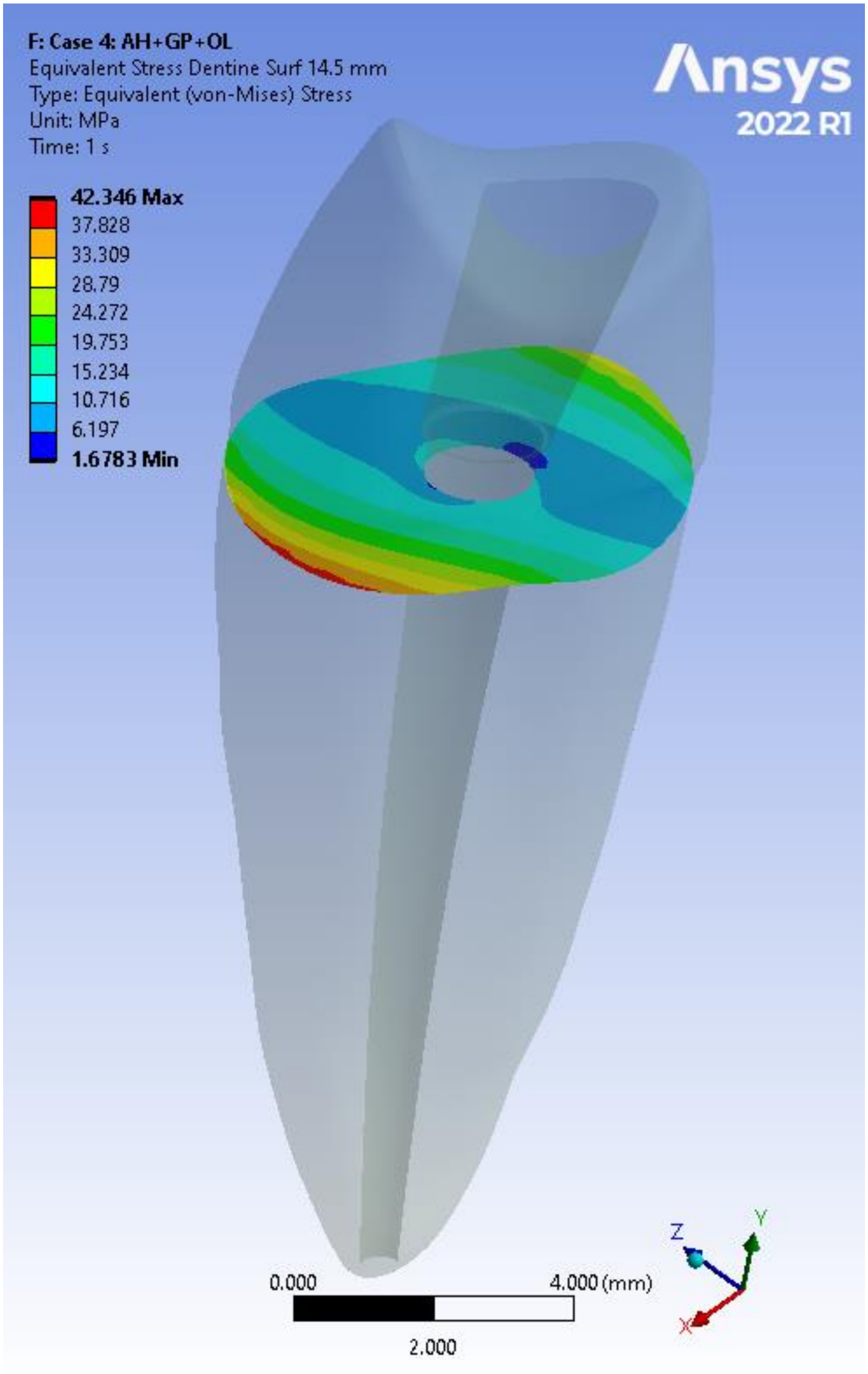

F: Case 4: AH+GP+OL
Equivalent Stress Dentine Surf 14.5 mm
Type: Equivalent (von-Mises) Stress
Unit: MPa
Time: 1 s
42.346 Max
37.828
33.309
28.79
24.272
19.753
15.234
10.716
6.197
1.6783 Min
Ansys
2022 R1
0.000
4.000 (mm)
2.000
Z
Y
X

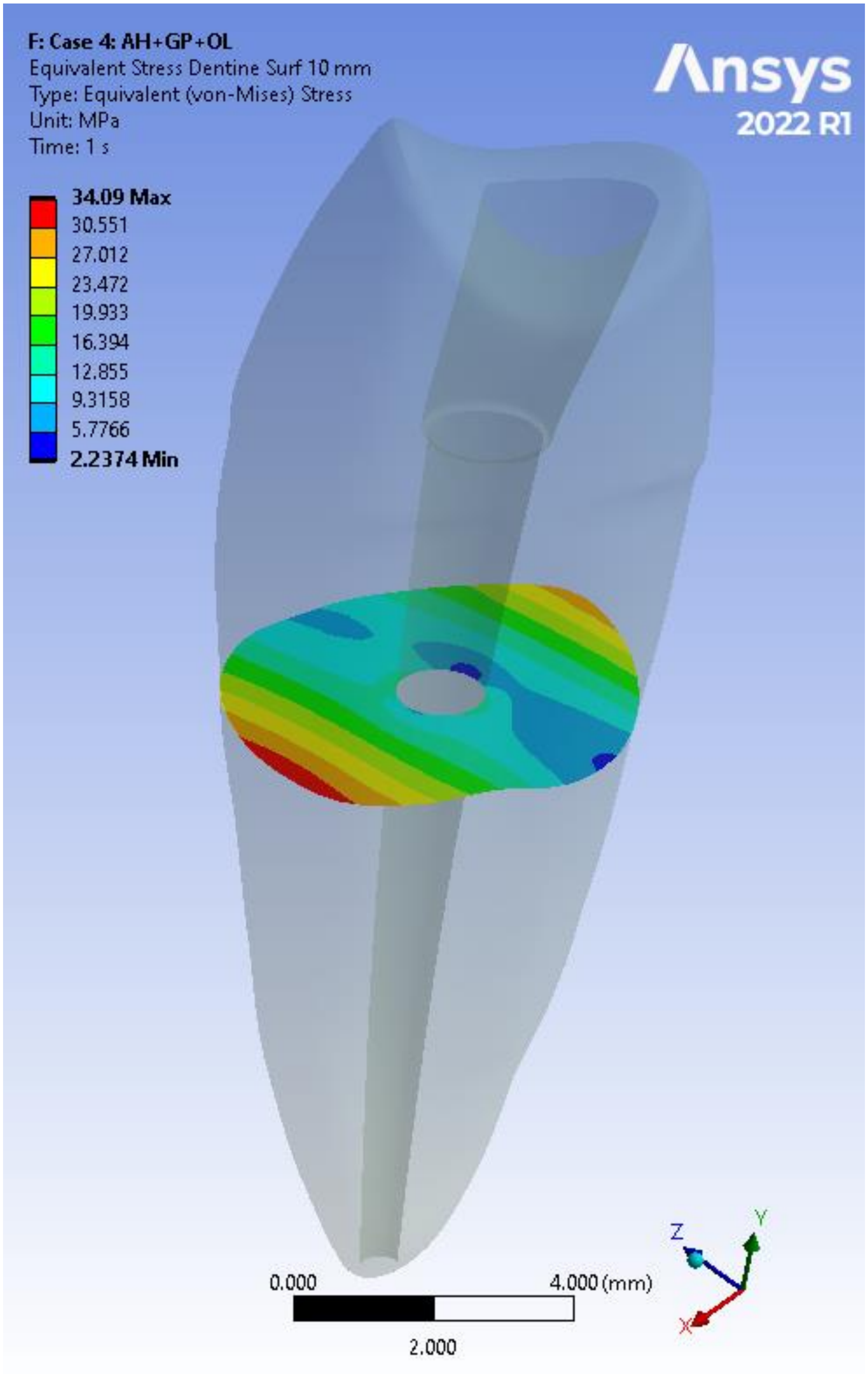
F: Case 4: AH+GP+OL
Equivalent Stress Dentine Surf 10 mm
Type: Equivalent (von-Mises) Stress
Unit: MPa
Time: 1 s
34.09 Max
30.551
27.012
23.472
19.933
16.394
12.855
9.3158
5.7766
2.2374 Min
Ansys
2022 R1
0.000
4.000 (mm)
2.000
Z
Y
X

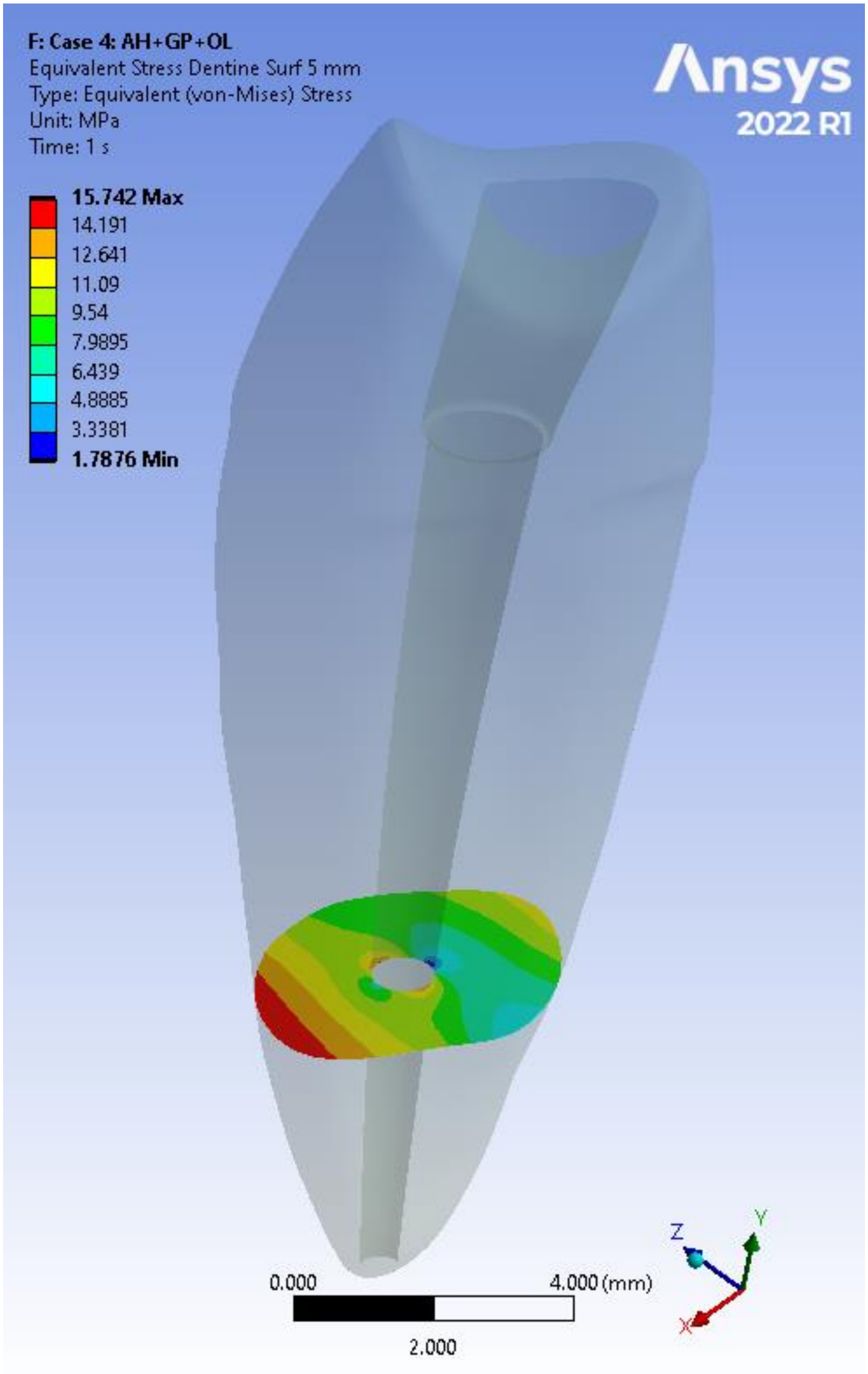
F: Case 4: AH+GP+OL
Equivalent Stress Dentine Surf 5 mm
Type: Equivalent (von-Mises) Stress
Unit: MPa
Time: 1 s
15.742 Max
14.191
12.641
11.09
9.54
7.9895
6.439
4.8885
3.3381
1.7876 Min
Ansys
2022 R1
0.000
4.000 (mm)
2.000
Z
Y
X

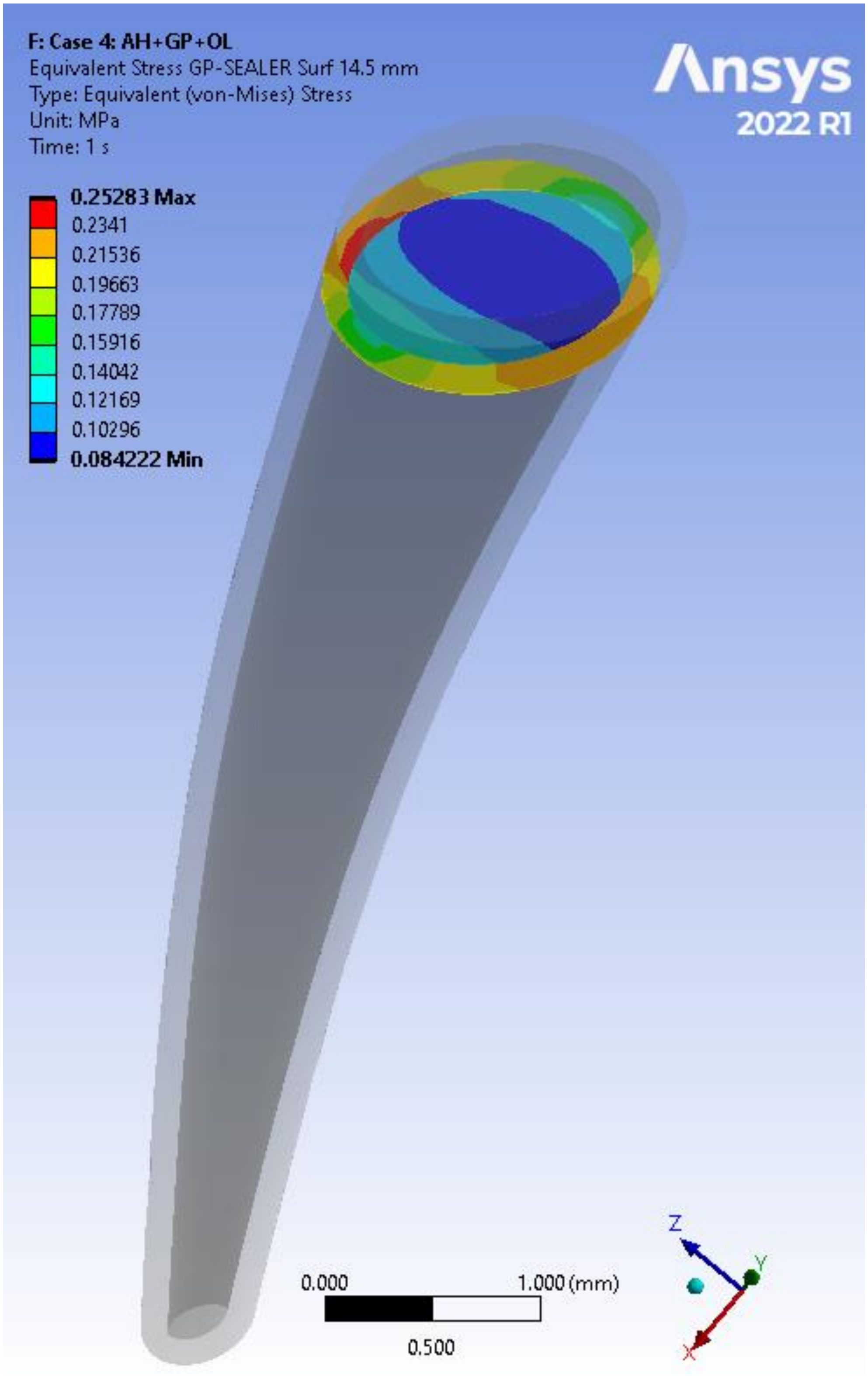

F: Case 4: AH+GP+OL
Equivalent Stress GP-SEALER Surf 14.5 mm
Type: Equivalent (von-Mises) Stress
Unit: MPa
Time: 1 s
Ansys
2022 R1
0.25283 Max
0.2341
0.21536
0.19663
0.17789
0.15916
0.14042
0.12169
0.10296
0.084222 Min
0.000
1.000 (mm)
0.500
Z
Y
X

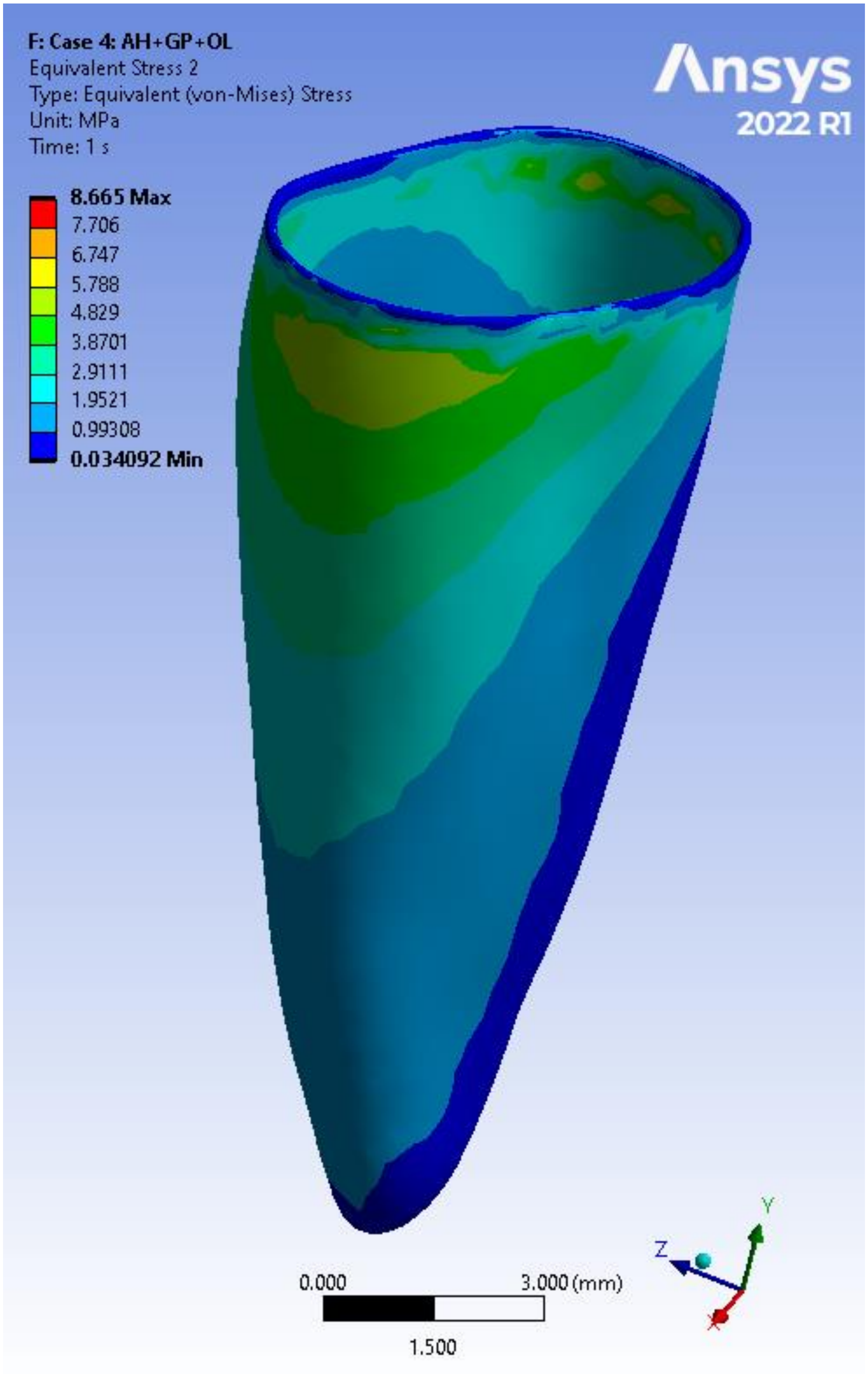
F: Case 4: AH+GP+OL
Equivalent Stress 2
Type: Equivalent (von-Mises) Stress
Unit: MPa
Time: 1 s
8.665 Max
7.706
6.747
5.788
4.829
3.8701
2.9111
1.9521
0.99308
0.034092 Min
Ansys
2022 R1
0.000
3.000 (mm)
1.500
Z
Y
X

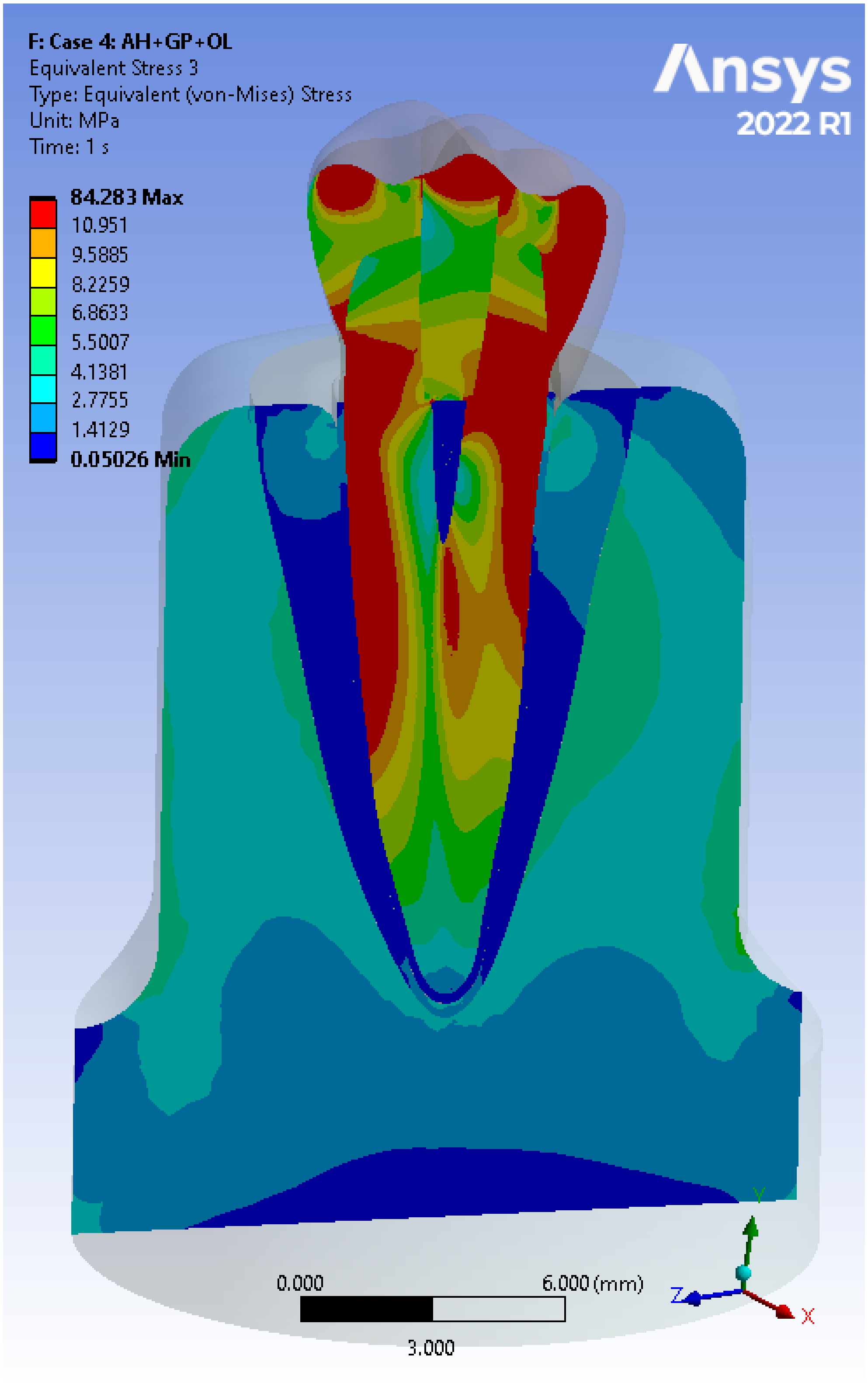

F: Case 4: AH+GP+OL
Equivalent Stress 3
Type: Equivalent (von-Mises) Stress
Unit: MPa
Time: 1 s
Ansys
2022 R1
84.283 Max
10.951
9.5885
8.2259
6.8633
5.5007
4.1381
2.7755
1.4129
0.05026 Min
0.000
6.000 (mm)
3.000
Y
Z
X

Case 5

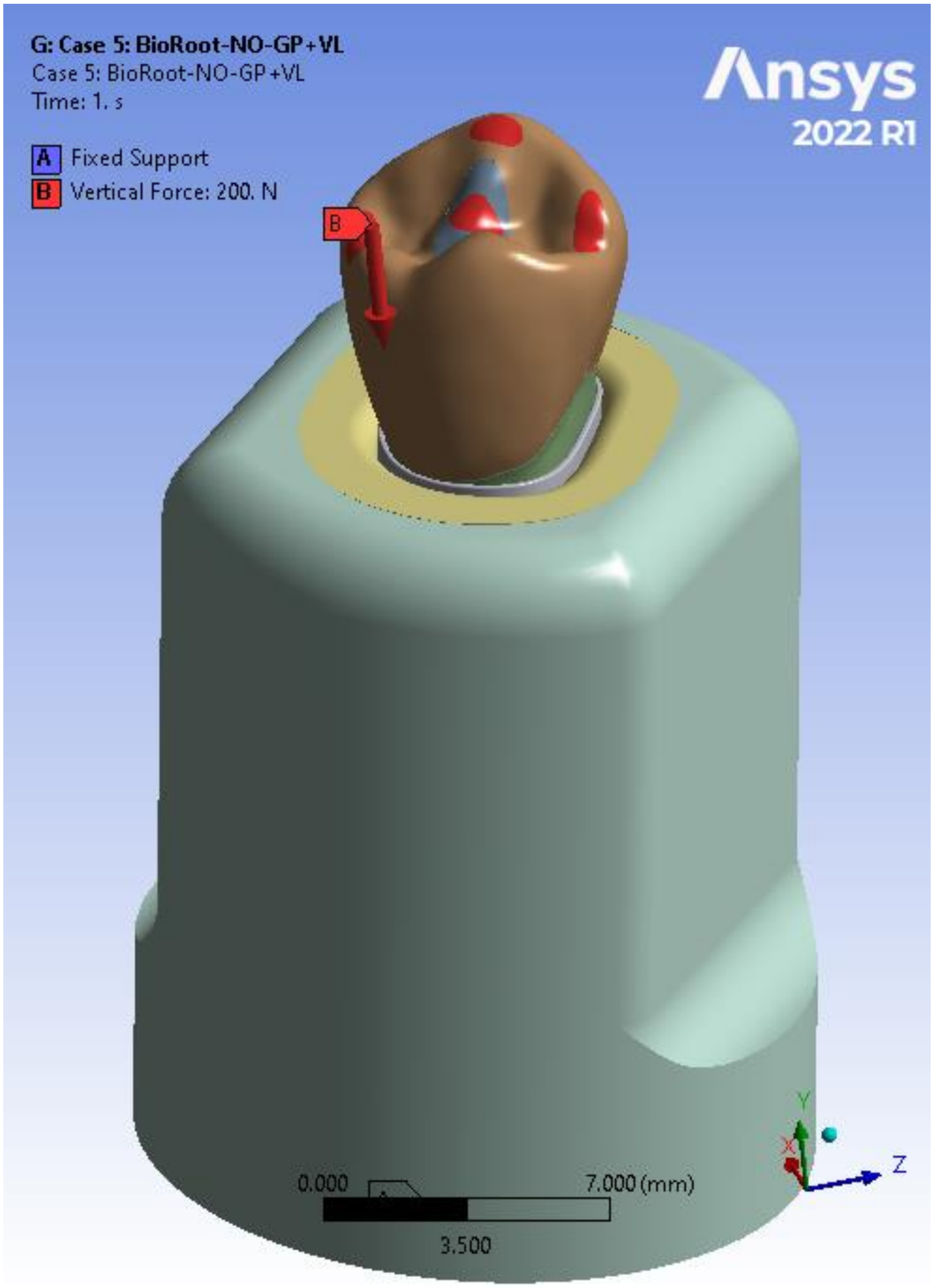

G: Case 5: BioRoot-NO-GP+VL
Case 5: BioRoot-NO-GP+VL
Time: 1. s
A Fixed Support
B Vertical Force: 200. N
Ansys
2022 R1
B
0.000
7.000 (mm)
3.500
Y
X
Z

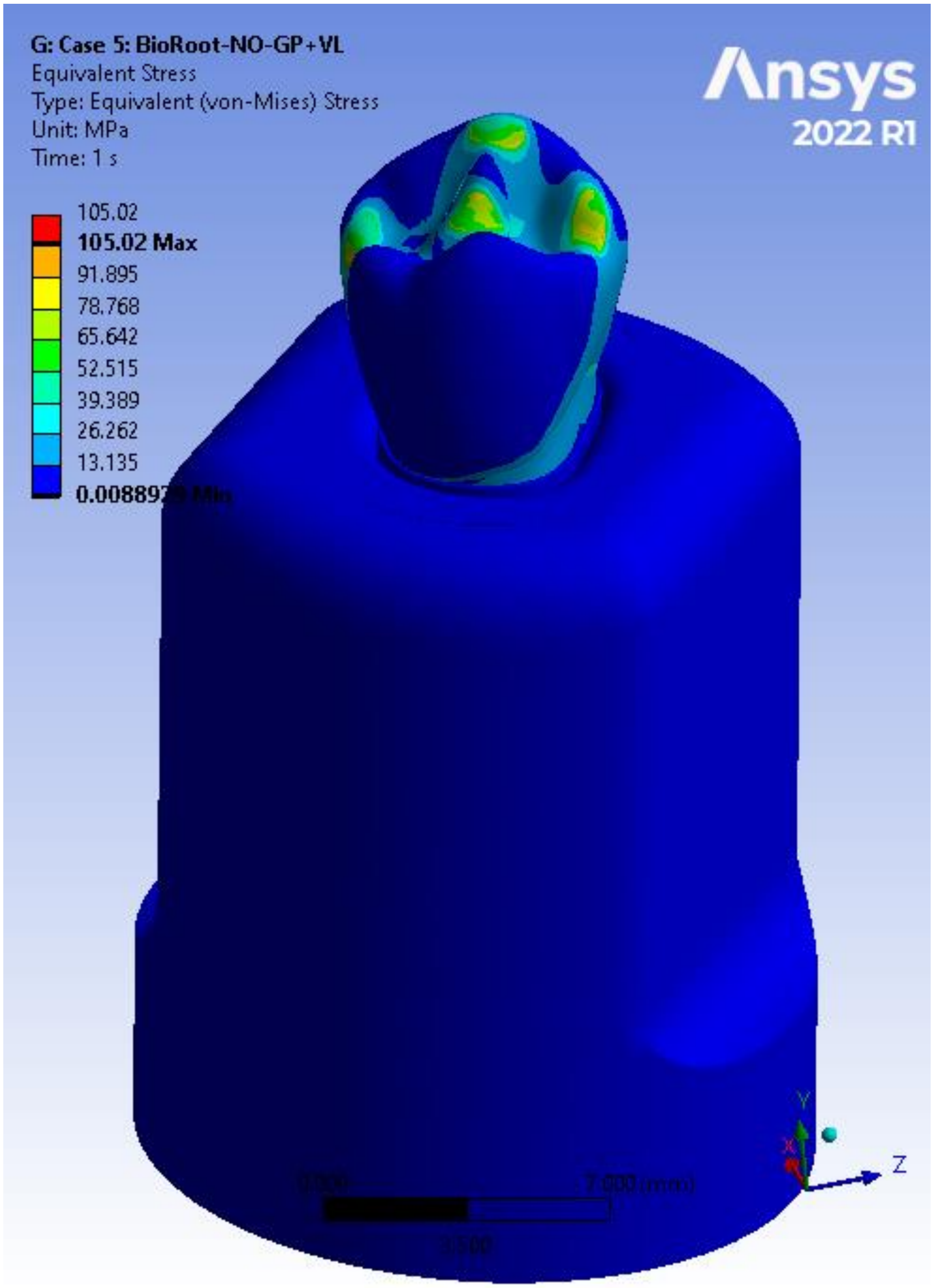
G: Case 5: BioRoot-NO-GP+VL
Equivalent Stress
Type: Equivalent (von-Mises) Stress
Unit: MPa
Time: 1 s
105.02
105.02 Max
91.895
78.768
65.642
52.515
39.389
26.262
13.135
Ansys
2022 R1
Y
X
Z

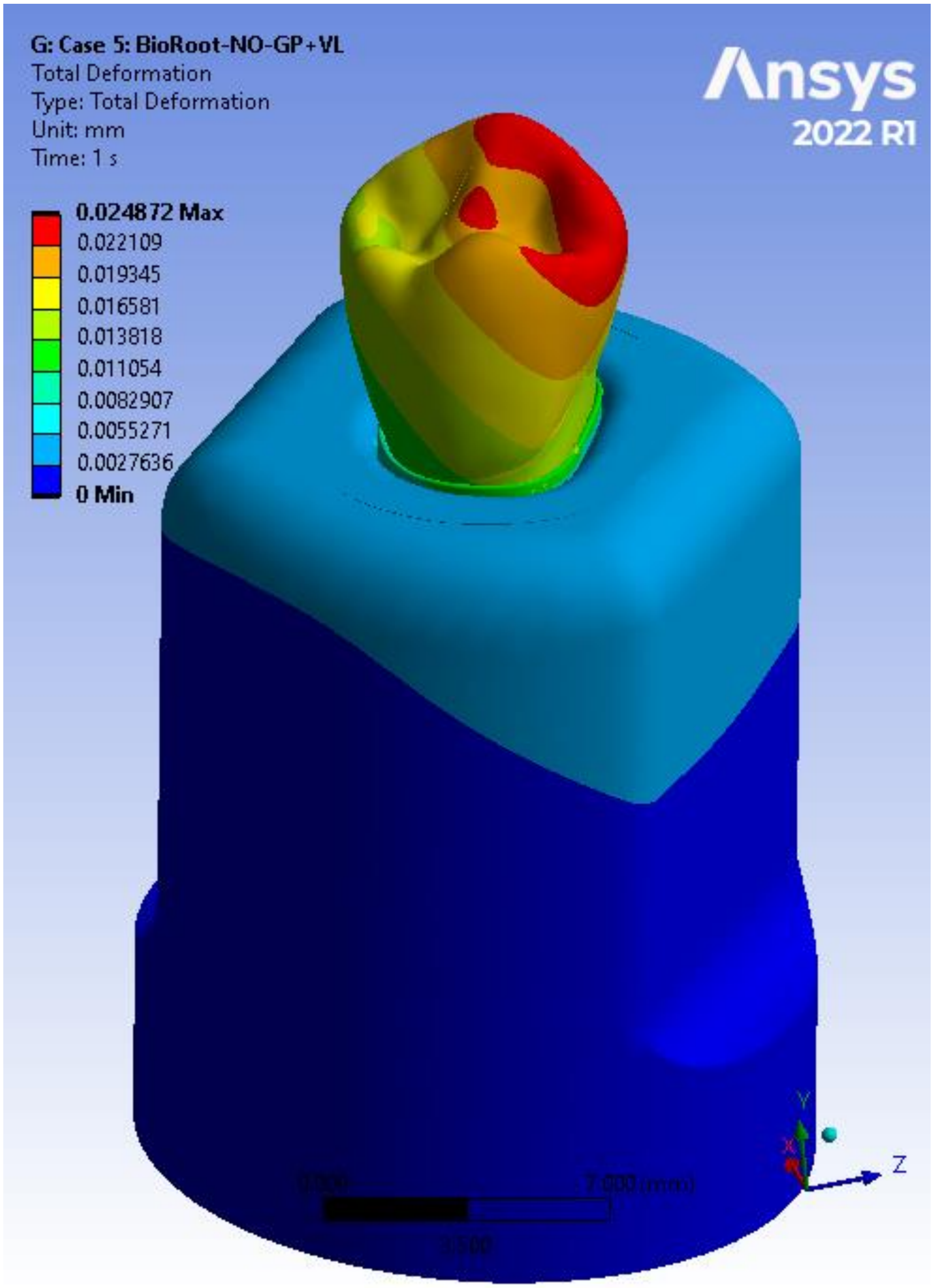
G: Case 5: BioRoot-NO-GP+VL
Total Deformation
Type: Total Deformation
Unit: mm
Time: 1 s
0.024872 Max
0.022109
0.019345
0.016581
0.013818
0.011054
0.0082907
0.0055271
0.0027636
0 Min
Ansys
2022 R1
Y
X
Z

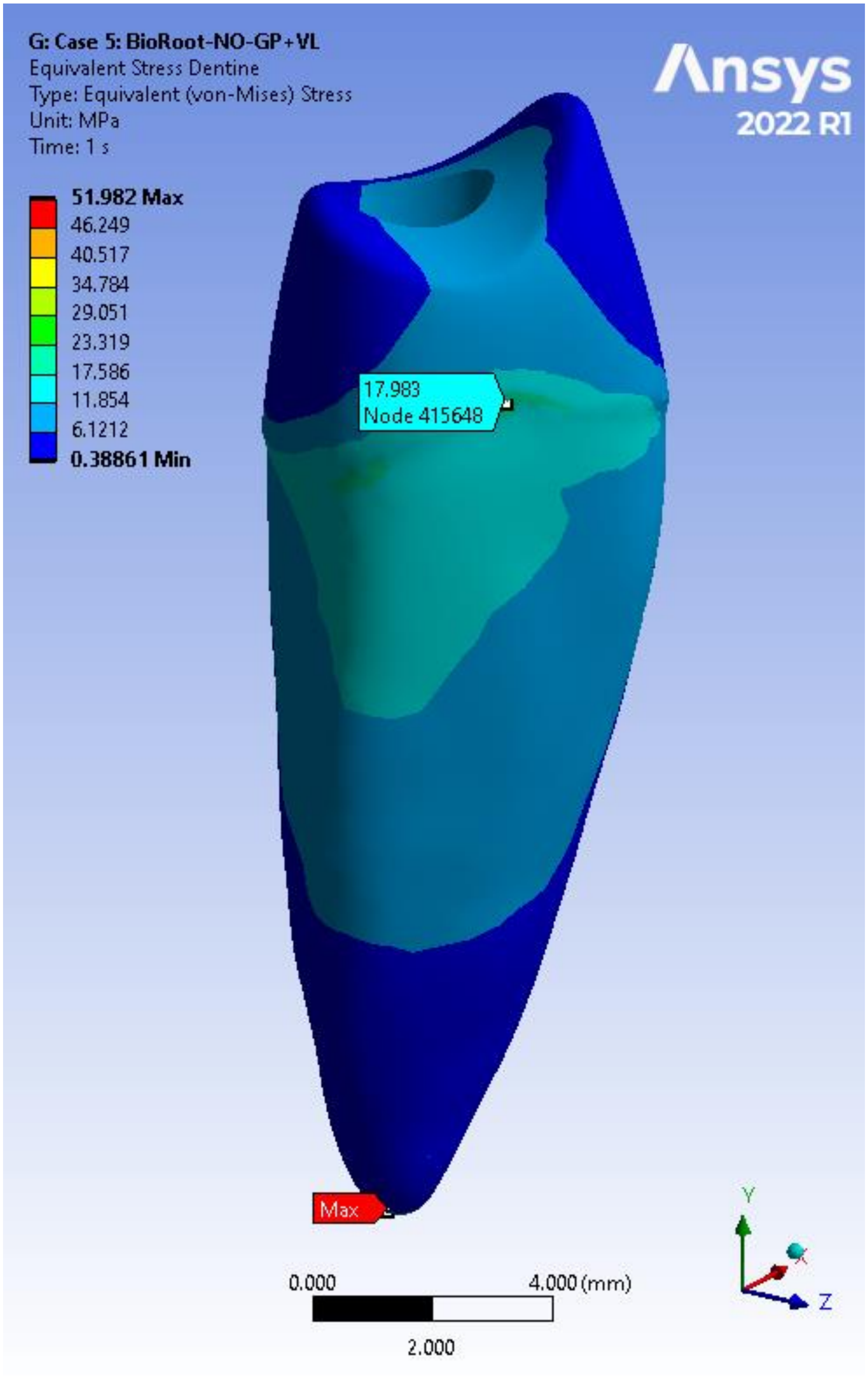
G: Case 5: BioRoot-NO-GP+VL
Equivalent Stress Dentine
Type: Equivalent (von-Mises) Stress
Unit: MPa
Time: 1 s
Ansys
2022 R1
51.982 Max
46.249
40.517
34.784
29.051
23.319
17.586
11.854
6.1212
0.38861 Min
17.983
Node 415648
Max
0.000
4.000 (mm)
2.000
Y
X
Z

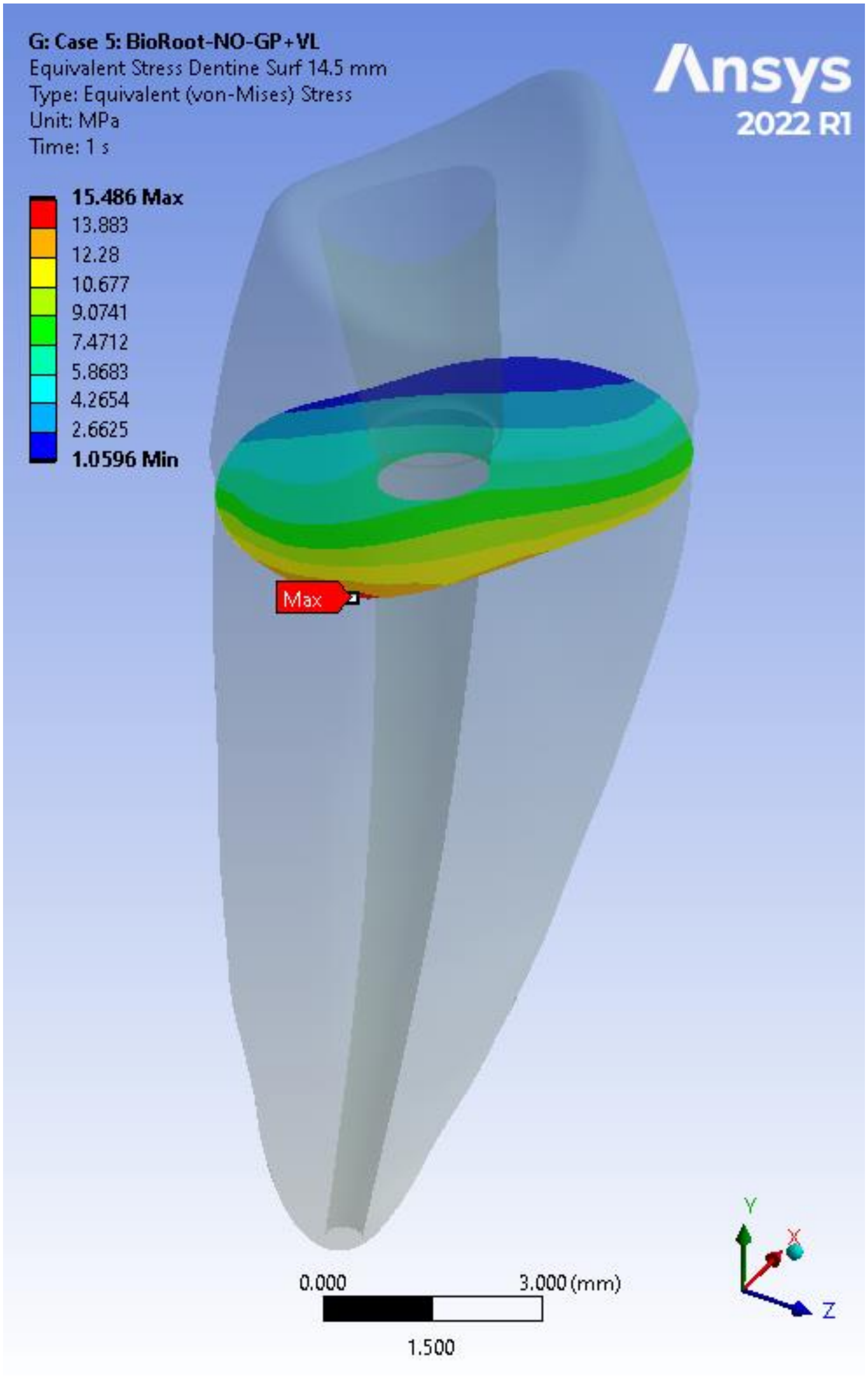
G: Case 5: BioRoot-NO-GP+VL
Equivalent Stress Dentine Surf 14.5 mm
Type: Equivalent (von-Mises) Stress
Unit: MPa
Time: 1 s
Ansys
2022 R1
15.486 Max
13.883
12.28
10.677
9.0741
7.4712
5.8683
4.2654
2.6625
1.0596 Min
Max
0.000
3.000 (mm)
1.500
Y
X
Z

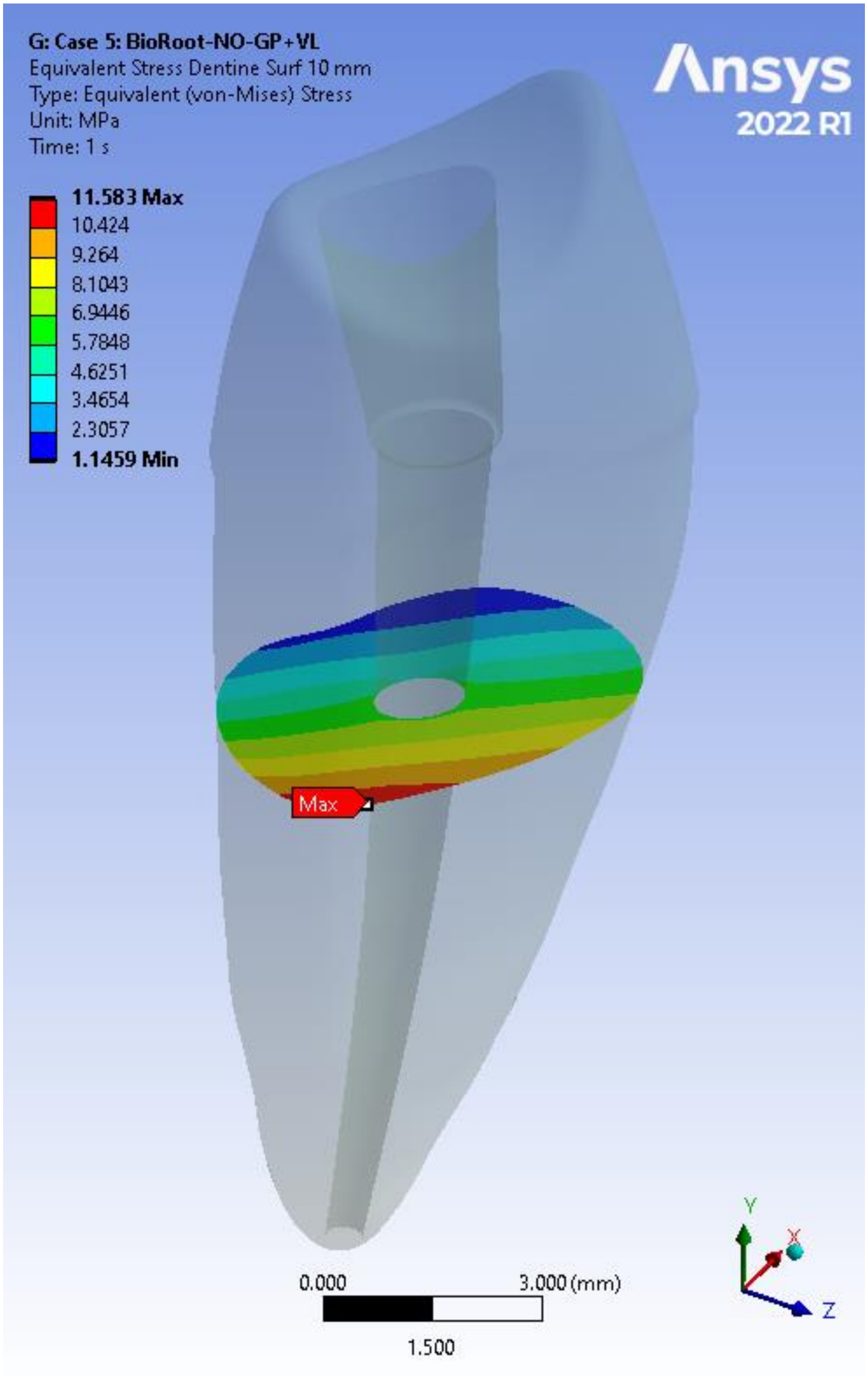
G: Case 5: BioRoot-NO-GP+VL
Equivalent Stress Dentine Surf 10 mm
Type: Equivalent (von-Mises) Stress
Unit: MPa
Time: 1 s
11.583 Max
10.424
9.264
8.1043
6.9446
5.7848
4.6251
3.4654
2.3057
1.1459 Min
Ansys
2022 R1
Max
0.000
3.000 (mm)
1.500
Y
X
Z

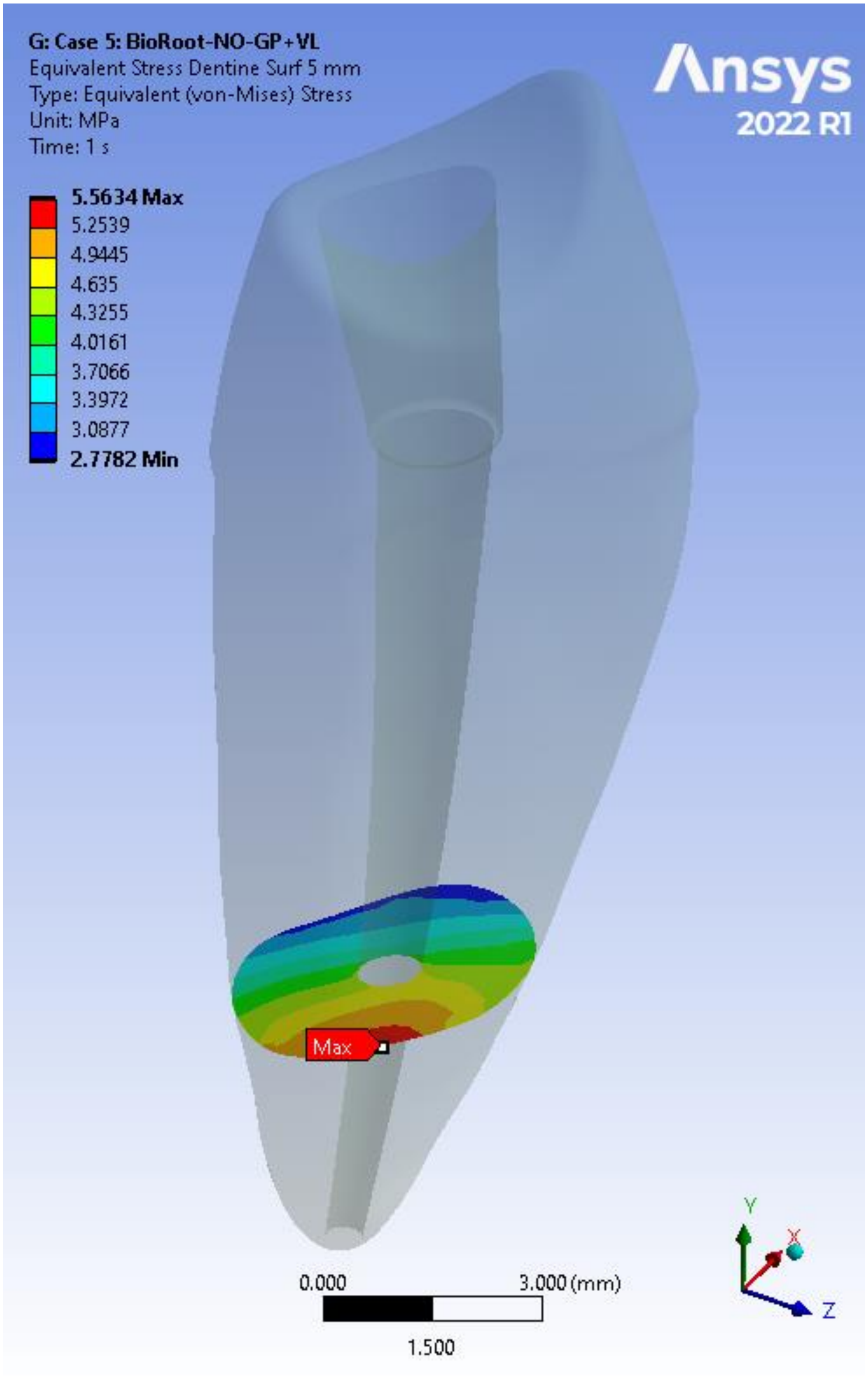
G: Case 5: BioRoot-NO-GP+VL
Equivalent Stress Dentine Surf 5 mm
Type: Equivalent (von-Mises) Stress
Unit: MPa
Time: 1 s
5.5634 Max
5.2539
4.9445
4.635
4.3255
4.0161
3.7066
3.3972
3.0877
2.7782 Min
Ansys
2022 R1
Max
0.000
3.000 (mm)
1.500
Y
X
Z

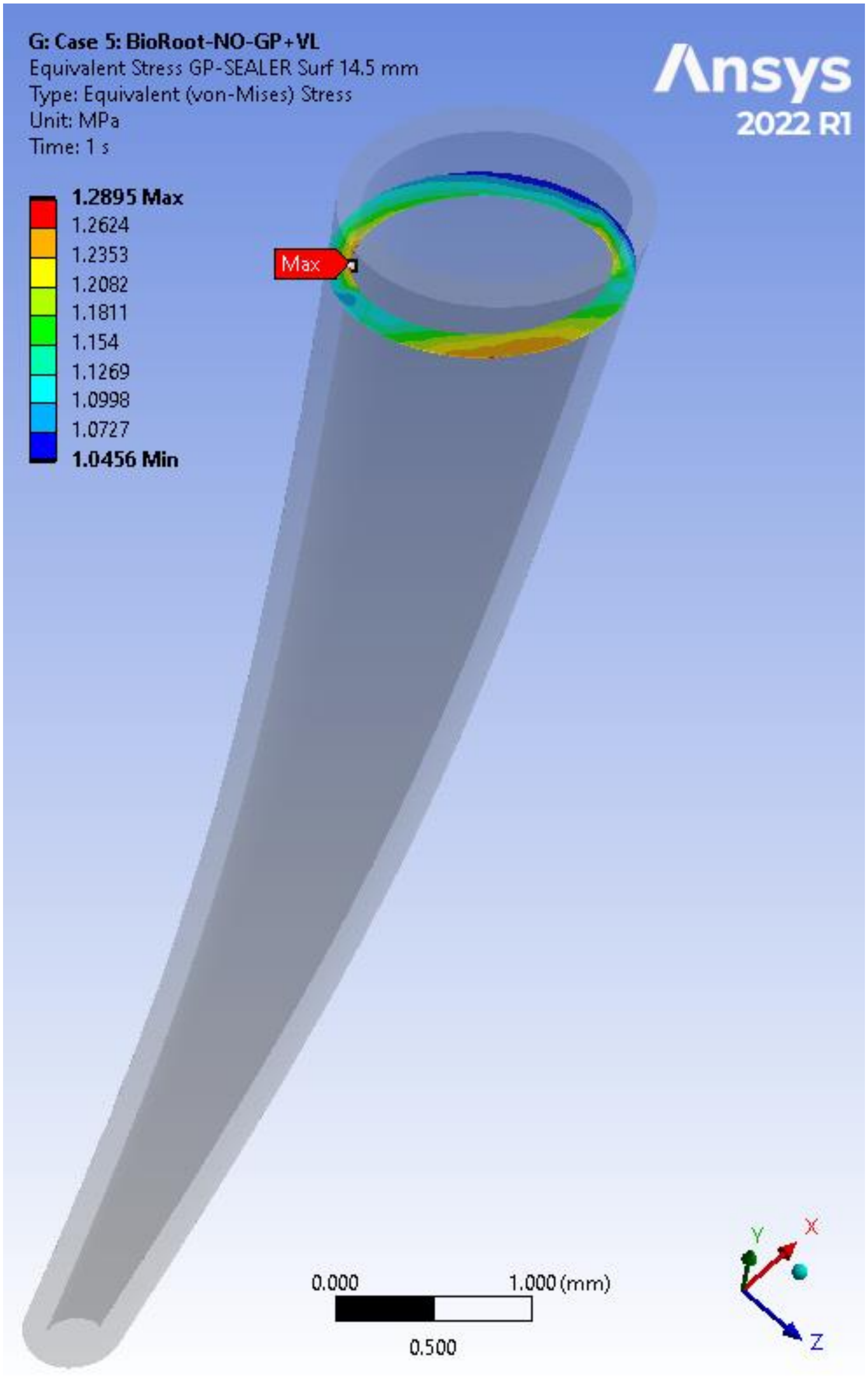

G: Case 5: BioRoot-NO-GP+VL
Equivalent Stress GP-SEALER Surf 14.5 mm
Type: Equivalent (von-Mises) Stress
Unit: MPa
Time: 1 s
1.2895 Max
1.2624
1.2353
1.2082
1.1811
1.154
1.1269
1.0998
1.0727
1.0456 Min
Max
Ansys
2022 R1
0.000
1.000 (mm)
0.500
Y
X
Z

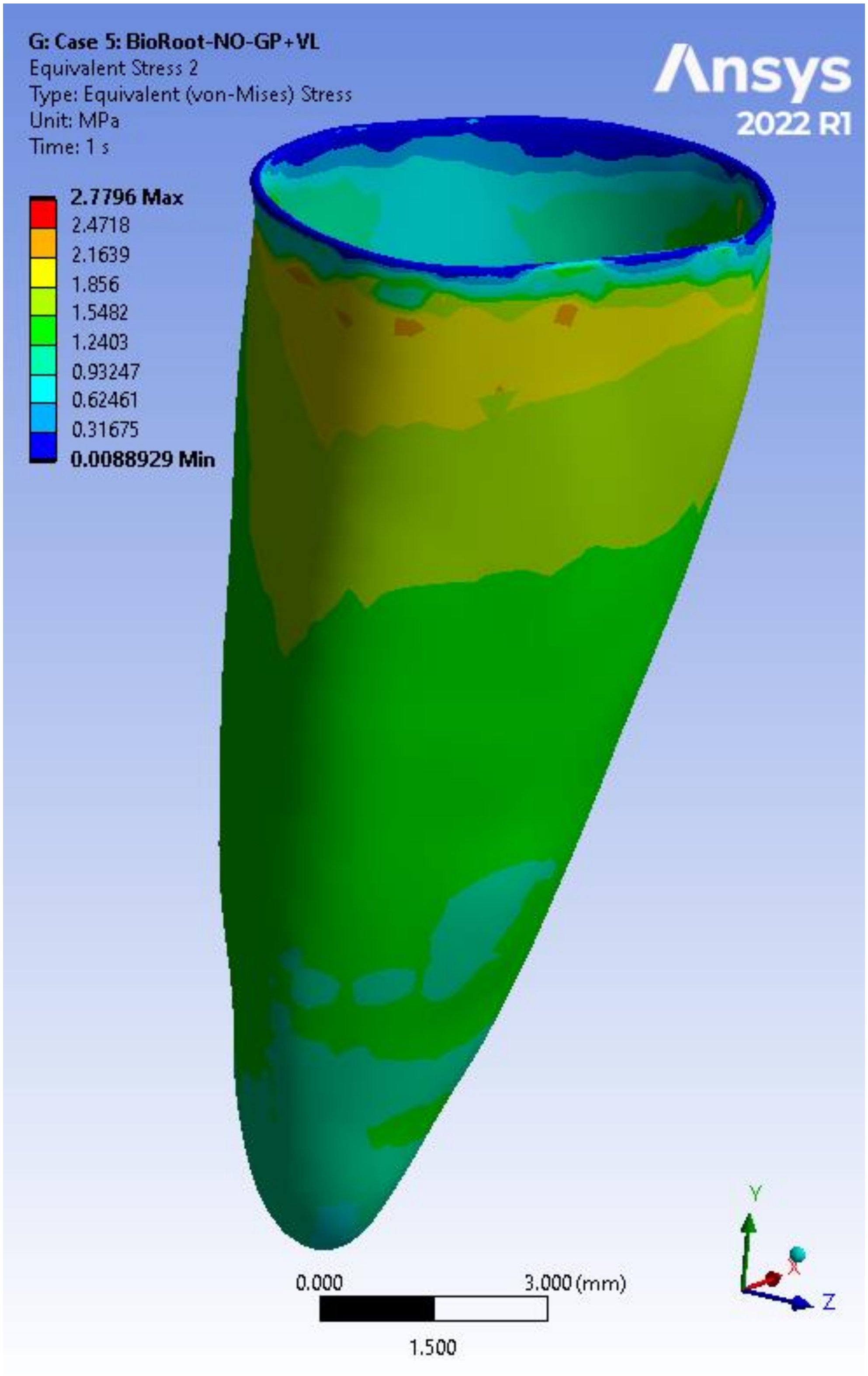
G: Case 5: BioRoot-NO-GP+VL
Equivalent Stress 2
Type: Equivalent (von-Mises) Stress
Unit: MPa
Time: 1 s
2.7796 Max
2.4718
2.1639
1.856
1.5482
1.2403
0.93247
0.62461
0.31675
0.0088929 Min
Ansys
2022 R1
0.000
3.000 (mm)
1.500
Y
X
Z

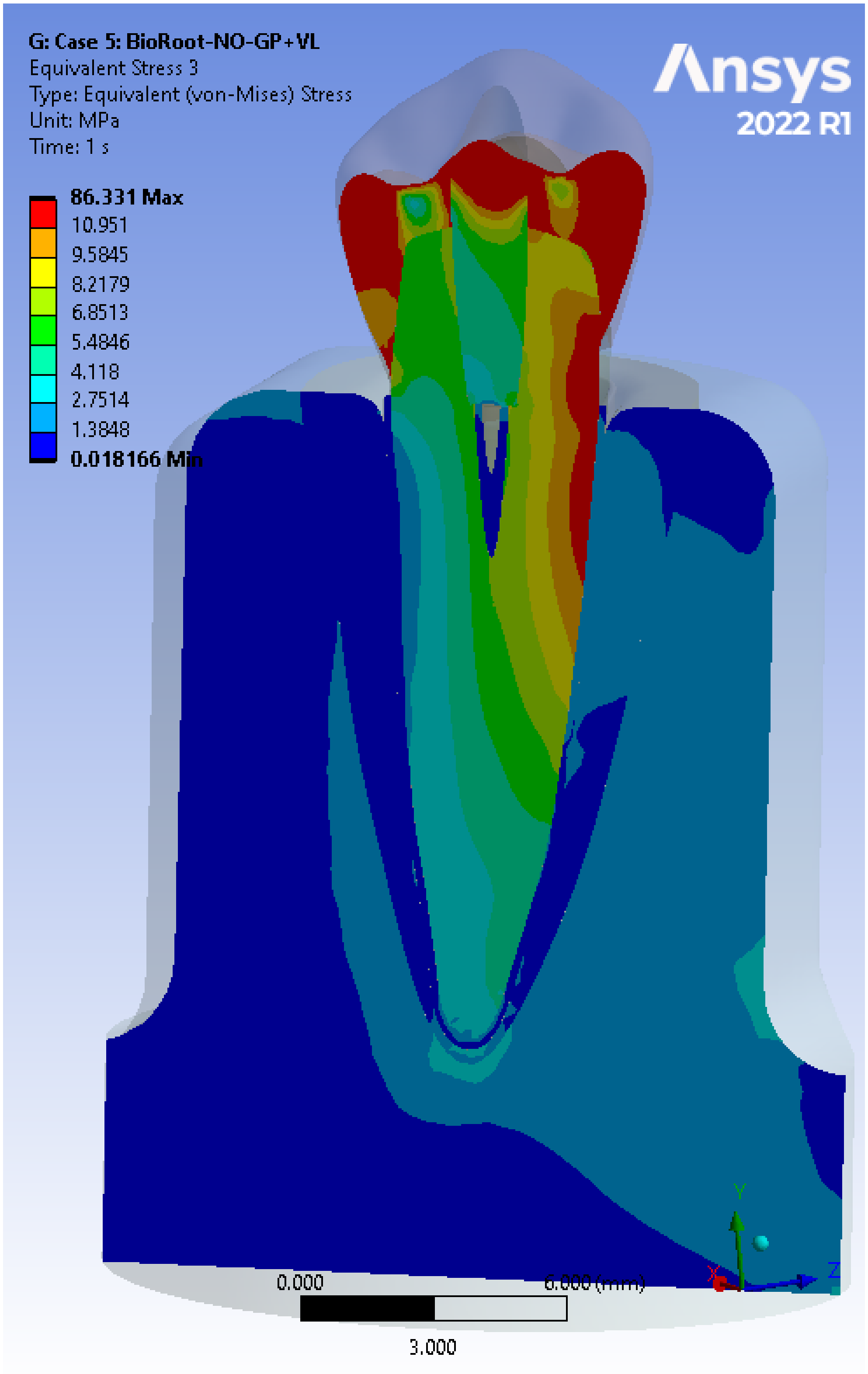
G: Case 5: BioRoot-NO-GP+VL
Equivalent Stress 3
Type: Equivalent (von-Mises) Stress
Unit: MPa
Time: 1 s
86.331 Max
10.951
9.5845
8.2179
6.8513
5.4846
4.118
2.7514
1.3848
0.018166 Min
Ansys
2022 R1
0.000
6.000 (mm)
3.000

Case 6

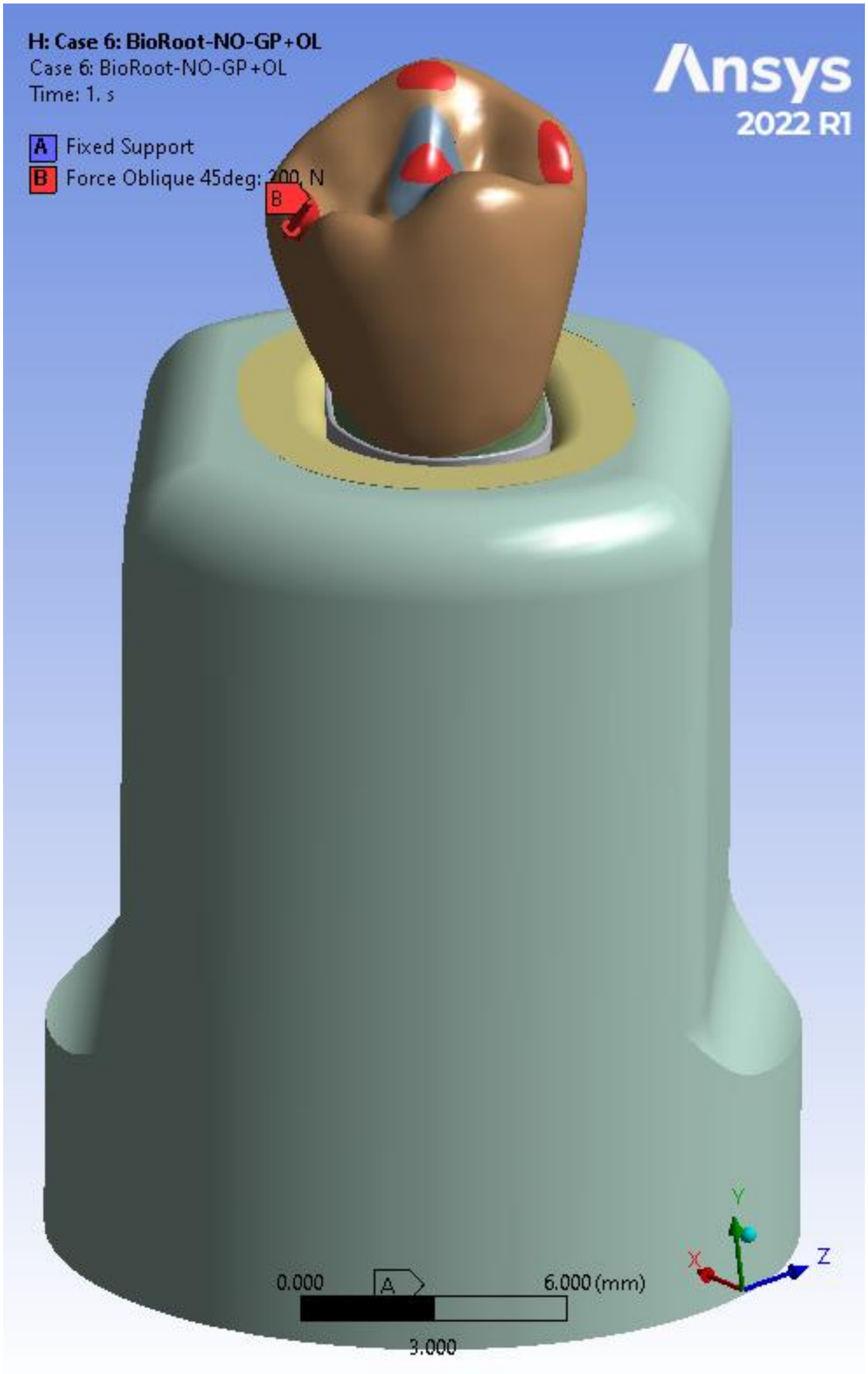
H: Case 6: BioRoot-NO-GP+OL
Case 6: BioRoot-NO-GP+OL
Time: 1. s
A Fixed Support
B Force Oblique 45deg: 200. N
Ansys
2022 R1
B
0.000
A
6.000 (mm)
3.000
Y
X
Z

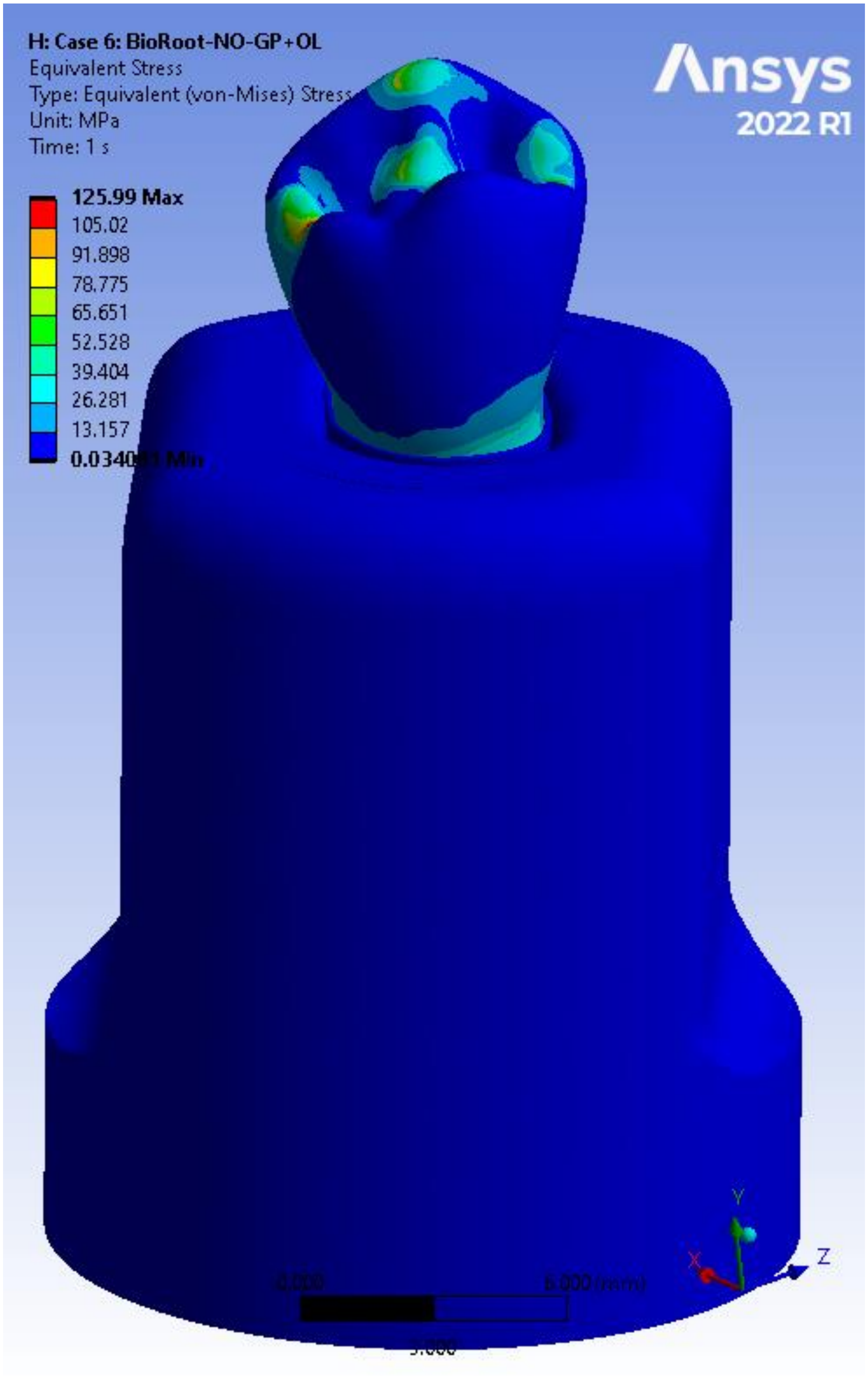
H: Case 6: BioRoot-NO-GP+OL
Equivalent Stress
Type: Equivalent (von-Mises) Stress
Unit: MPa
Time: 1 s
125.99 Max
105.02
91.898
78.775
65.651
52.528
39.404
26.281
13.157
Ansys
2022 R1
Y
Z
X

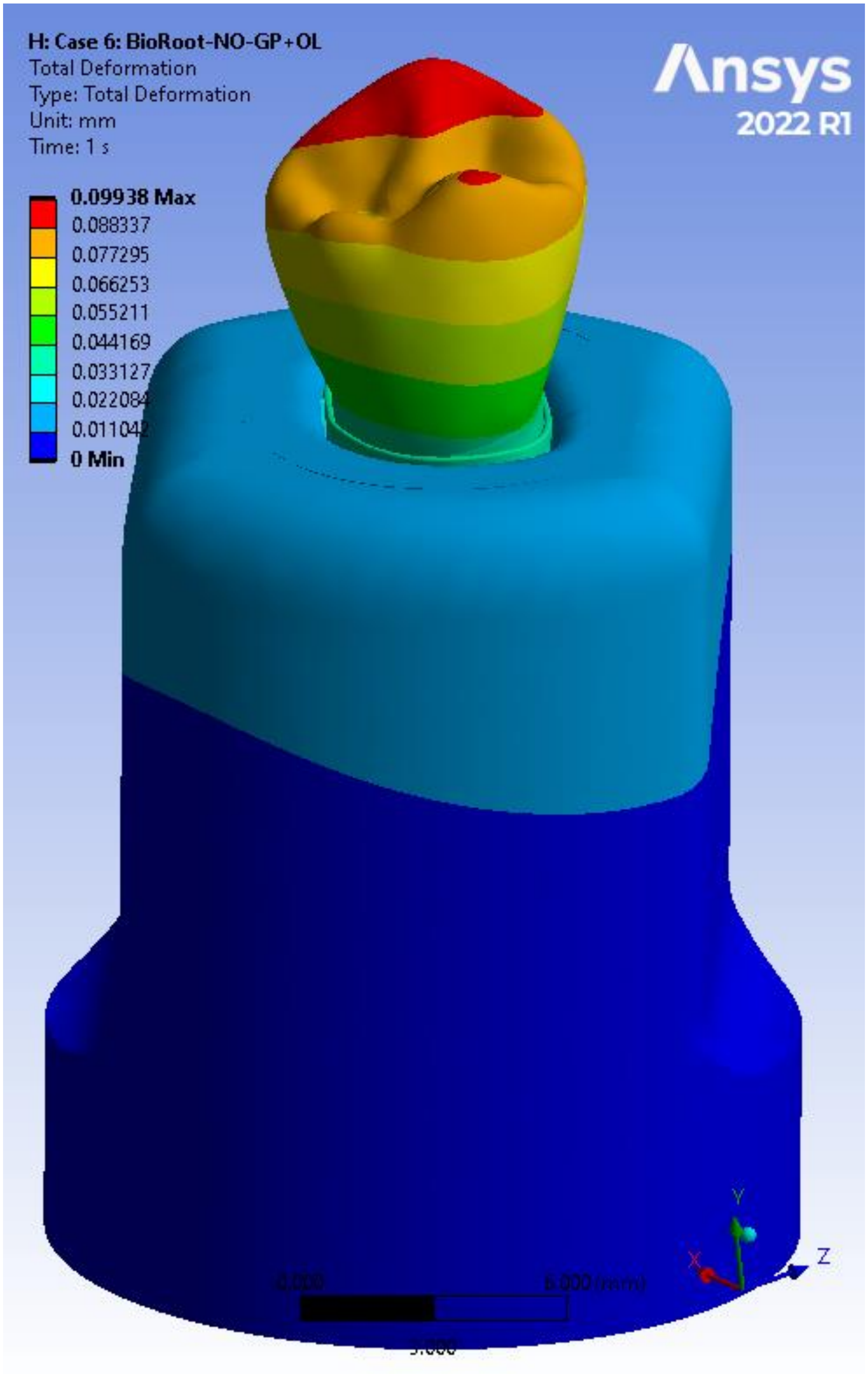
H: Case 6: BioRoot-NO-GP+OL
Total Deformation
Type: Total Deformation
Unit: mm
Time: 1 s
0.09938 Max
0.088337
0.077295
0.066253
0.055211
0.044169
0.033127
0.022084
0.011042
0 Min
Ansys
2022 R1
6.000 (mm)
Y
X
Z

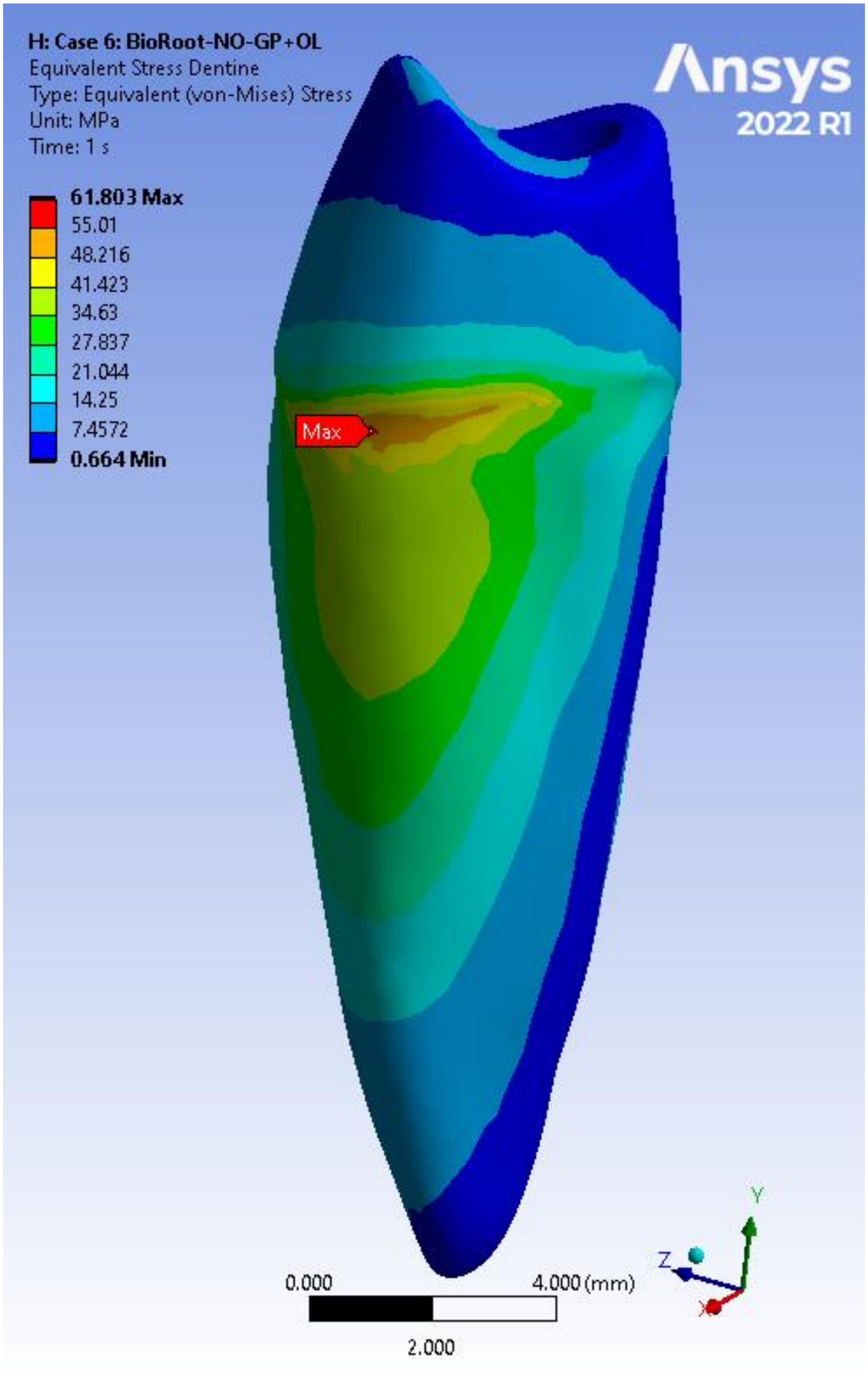
H: Case 6: BioRoot-NO-GP+OL
Equivalent Stress Dentine
Type: Equivalent (von-Mises) Stress
Unit: MPa
Time: 1 s
61.803 Max
55.01
48.216
41.423
34.63
27.837
21.044
14.25
7.4572
0.664 Min
Max
Ansys
2022 R1
0.000
4.000 (mm)
2.000
Y
Z
X

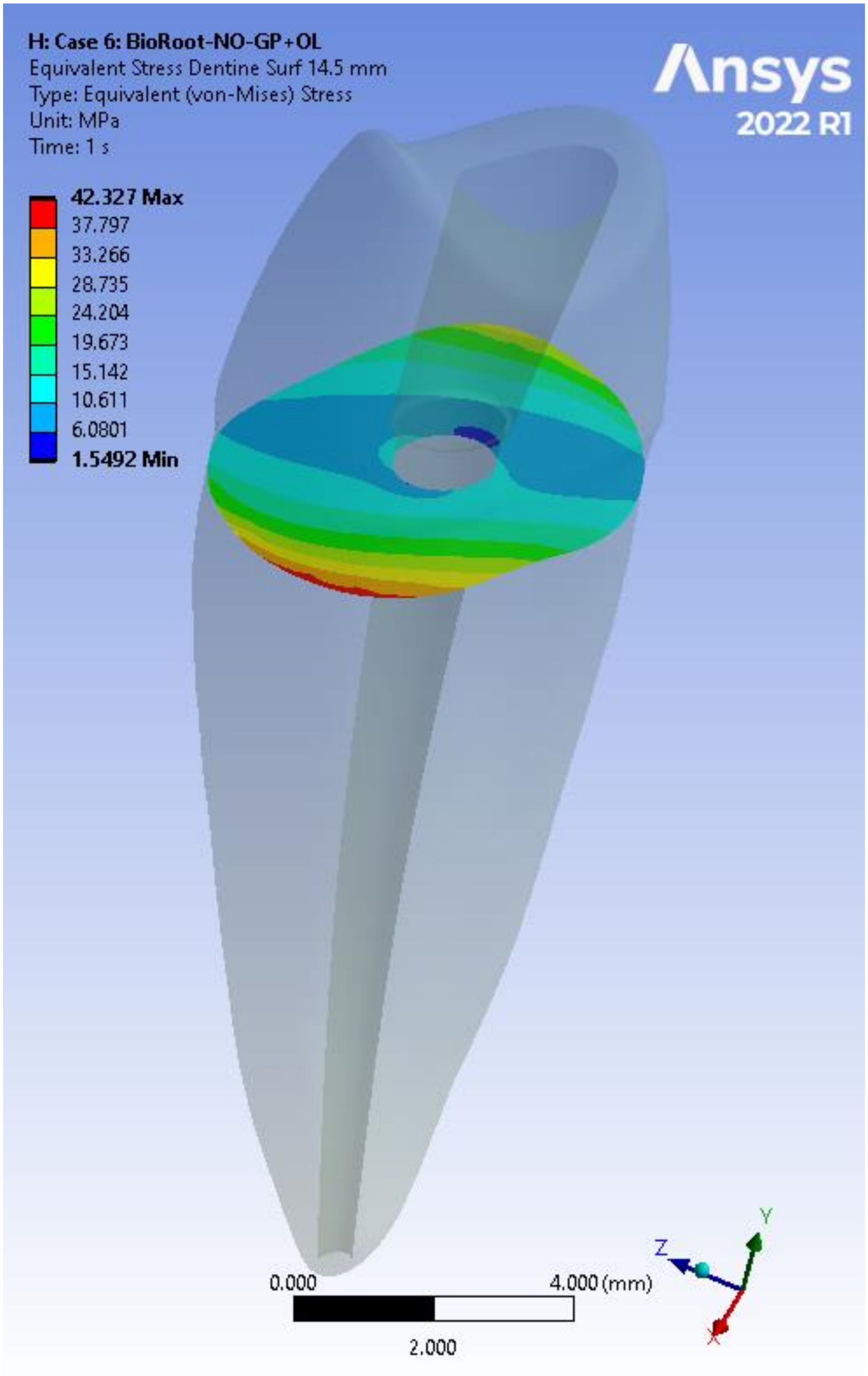
H: Case 6: BioRoot-NO-GP+OL
Equivalent Stress Dentine Surf 14.5 mm
Type: Equivalent (von-Mises) Stress
Unit: MPa
Time: 1 s
42.327 Max
37.797
33.266
28.735
24.204
19.673
15.142
10.611
6.0801
1.5492 Min
Ansys
2022 R1
0.000
4.000 (mm)
2.000
Y
Z
X

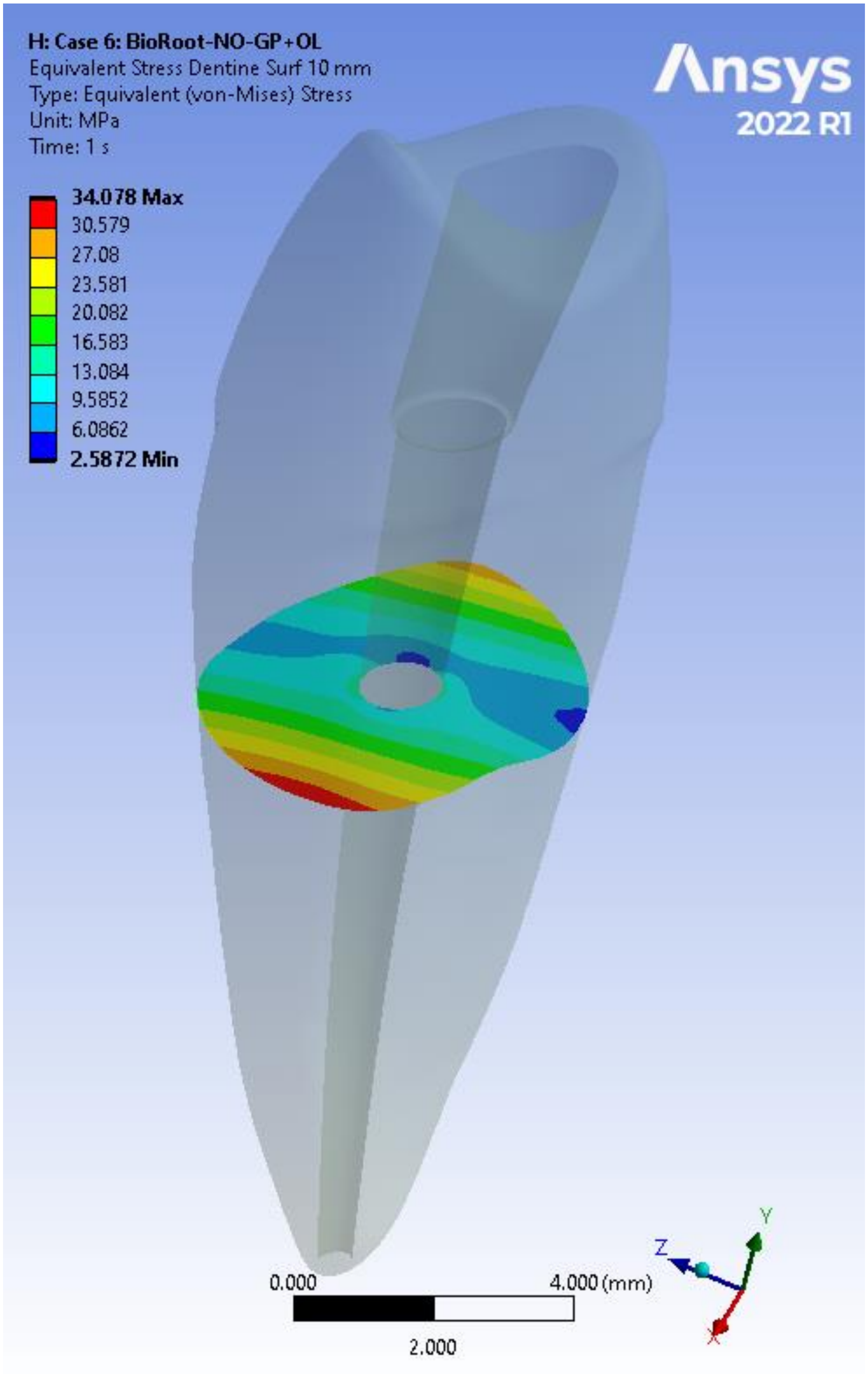

H: Case 6: BioRoot-NO-GP+OL
Equivalent Stress Dentine Surf 10 mm
Type: Equivalent (von-Mises) Stress
Unit: MPa
Time: 1 s
34.078 Max
30.579
27.08
23.581
20.082
16.583
13.084
9.5852
6.0862
2.5872 Min
Ansys
2022 R1
0.000
4.000 (mm)
2.000
Z
Y
X

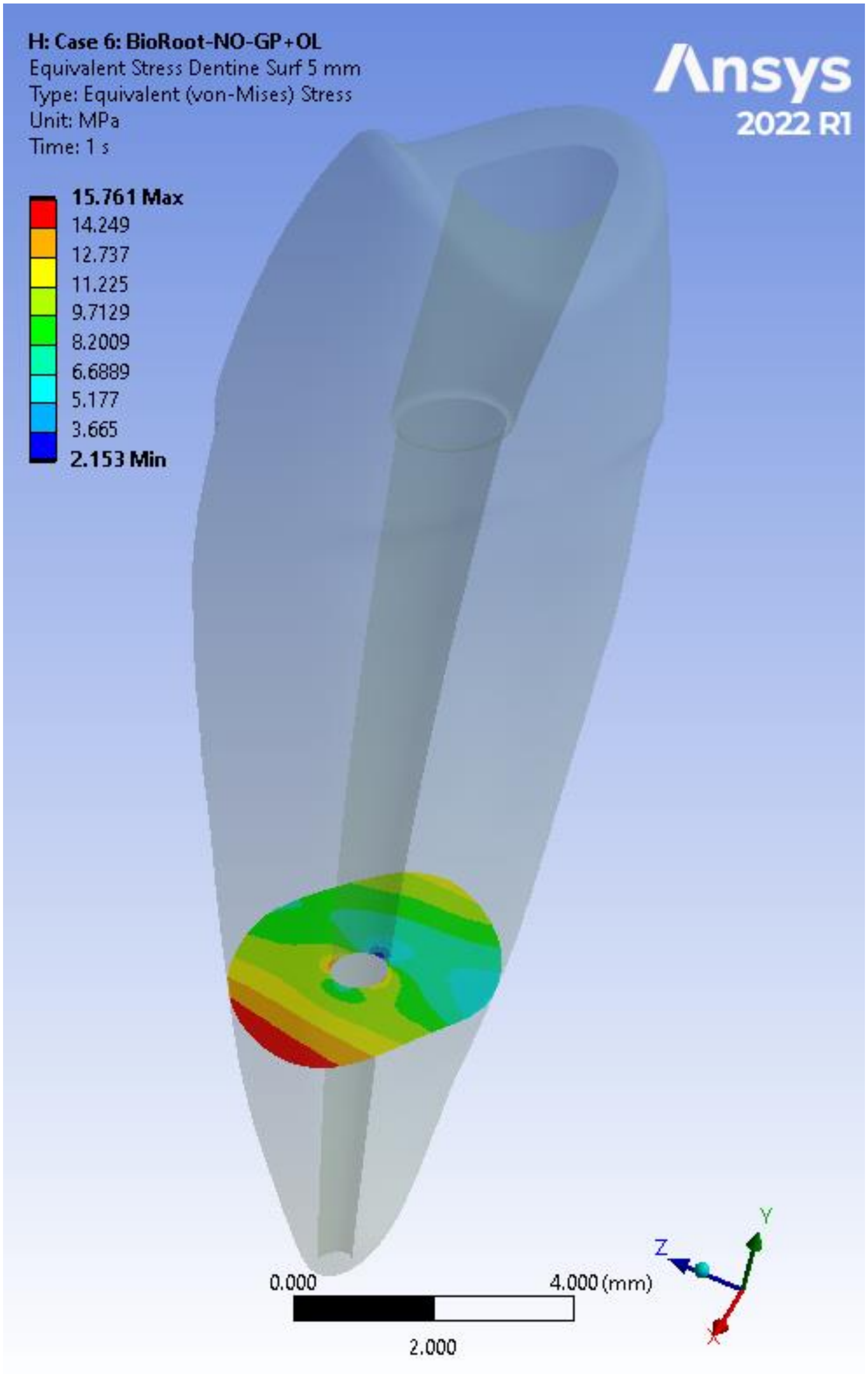
H: Case 6: BioRoot-NO-GP+OL
Equivalent Stress Dentine Surf 5 mm
Type: Equivalent (von-Mises) Stress
Unit: MPa
Time: 1 s
15.761 Max
14.249
12.737
11.225
9.7129
8.2009
6.6889
5.177
3.665
2.153 Min
Ansys
2022 R1
0.000
4.000 (mm)
2.000
Z
Y
X

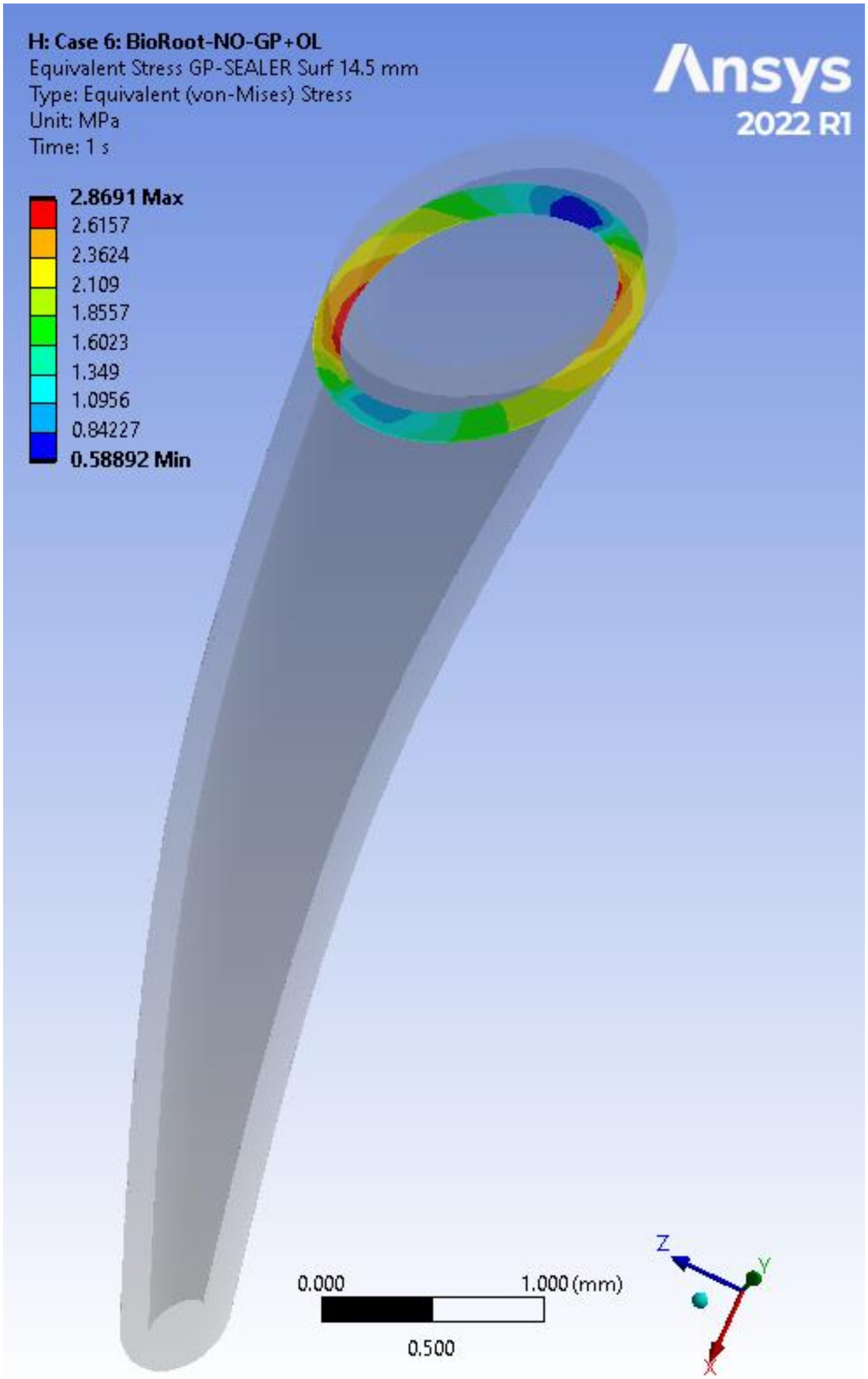
H: Case 6: BioRoot-NO-GP+OL
Equivalent Stress GP-SEALER Surf 14.5 mm
Type: Equivalent (von-Mises) Stress
Unit: MPa
Time: 1 s
2.8691 Max
2.6157
2.3624
2.109
1.8557
1.6023
1.349
1.0956
0.84227
0.58892 Min
Ansys
2022 R1
0.000
1.000 (mm)
0.500
Z
Y
X

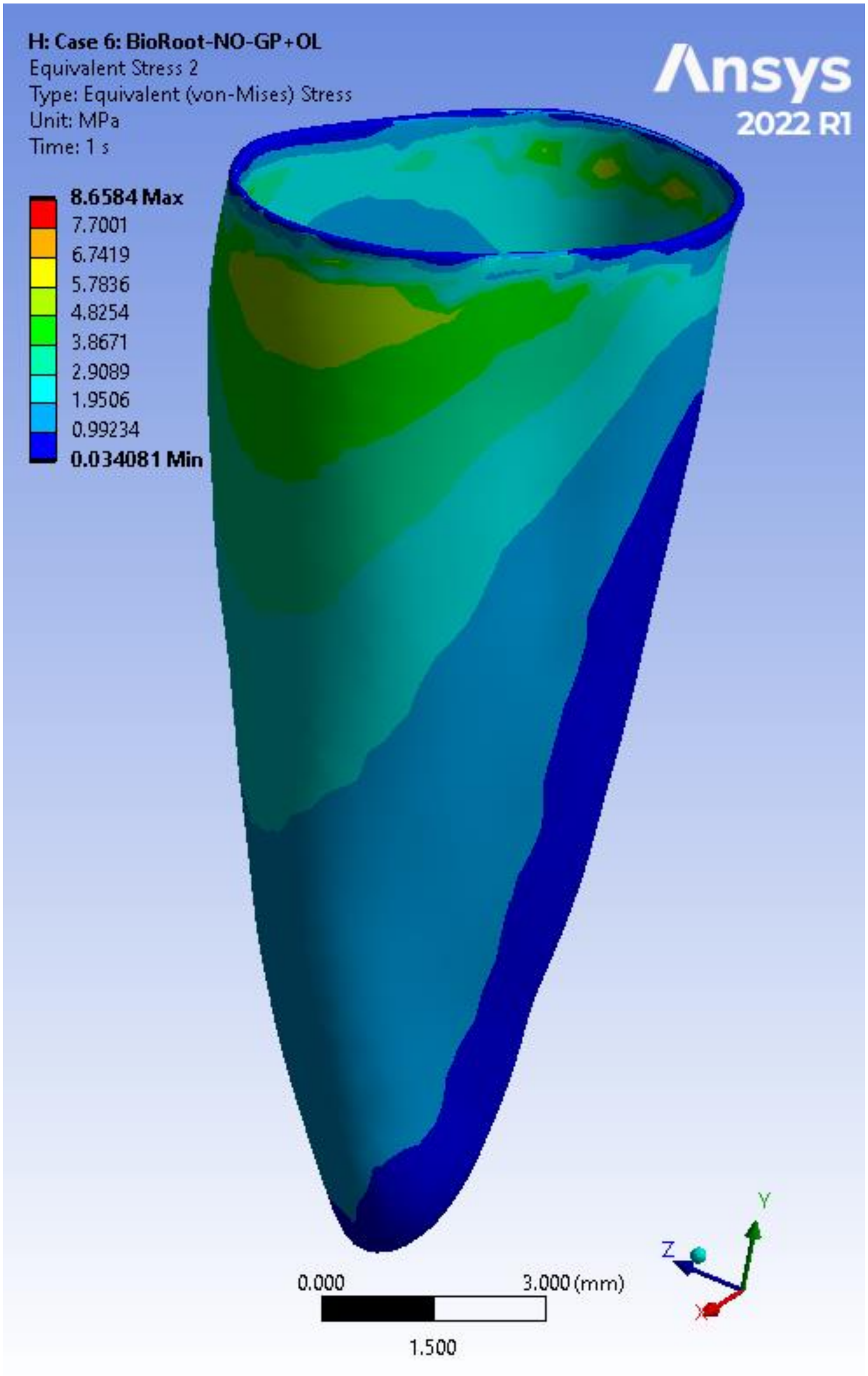
H: Case 6: BioRoot-NO-GP+OL
Equivalent Stress 2
Type: Equivalent (von-Mises) Stress
Unit: MPa
Time: 1 s
8.6584 Max
7.7001
6.7419
5.7836
4.8254
3.8671
2.9089
1.9506
0.99234
0.034081 Min
Ansys
2022 R1
0.000
3.000 (mm)
1.500
Y
Z
X

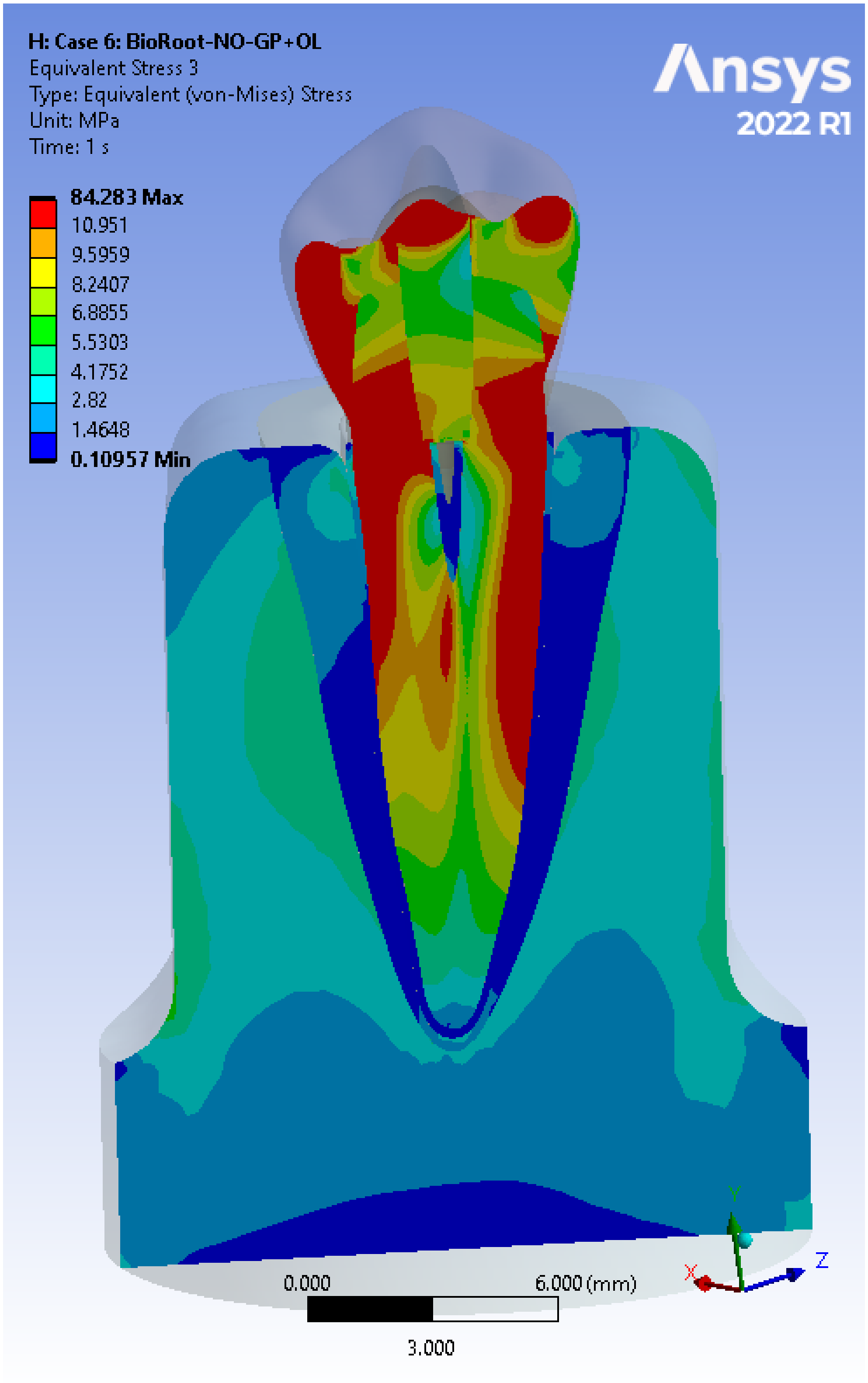

H: Case 6: BioRoot-NO-GP+OL
Equivalent Stress 3
Type: Equivalent (von-Mises) Stress
Unit: MPa
Time: 1 s
84.283 Max
10.951
9.5959
8.2407
6.8855
5.5303
4.1752
2.82
1.4648
0.10957 Min
Ansys
2022 R1
0.000
6.000 (mm)
3.000
Y
X
Z

Case 7

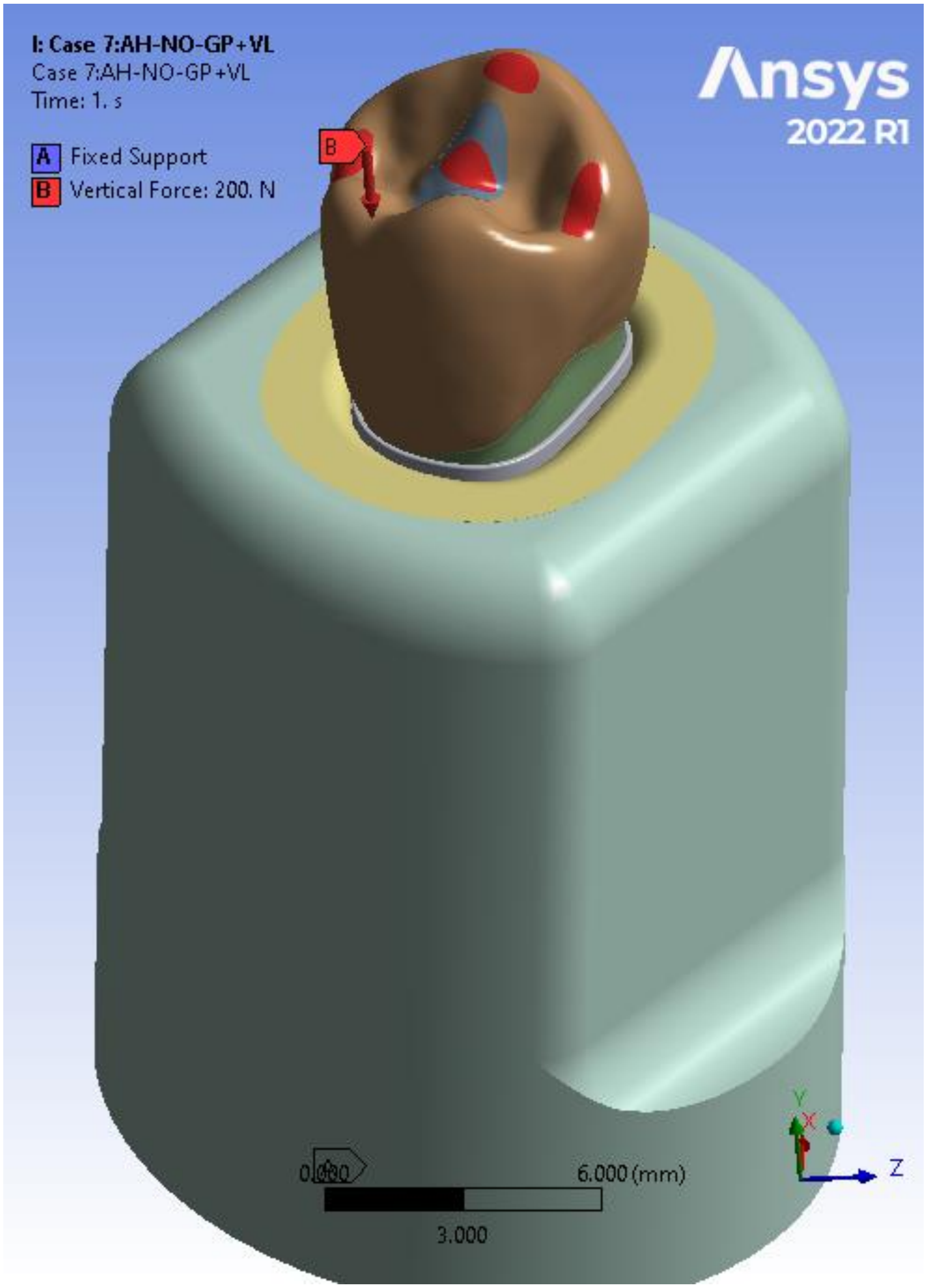

I: Case 7:AH-NO-GP+VL
Case 7:AH-NO-GP+VL
Time: 1. s
A Fixed Support
B Vertical Force: 200. N
Ansys
2022 R1
B
0.000
6.000 (mm)
3.000
Y
X
Z

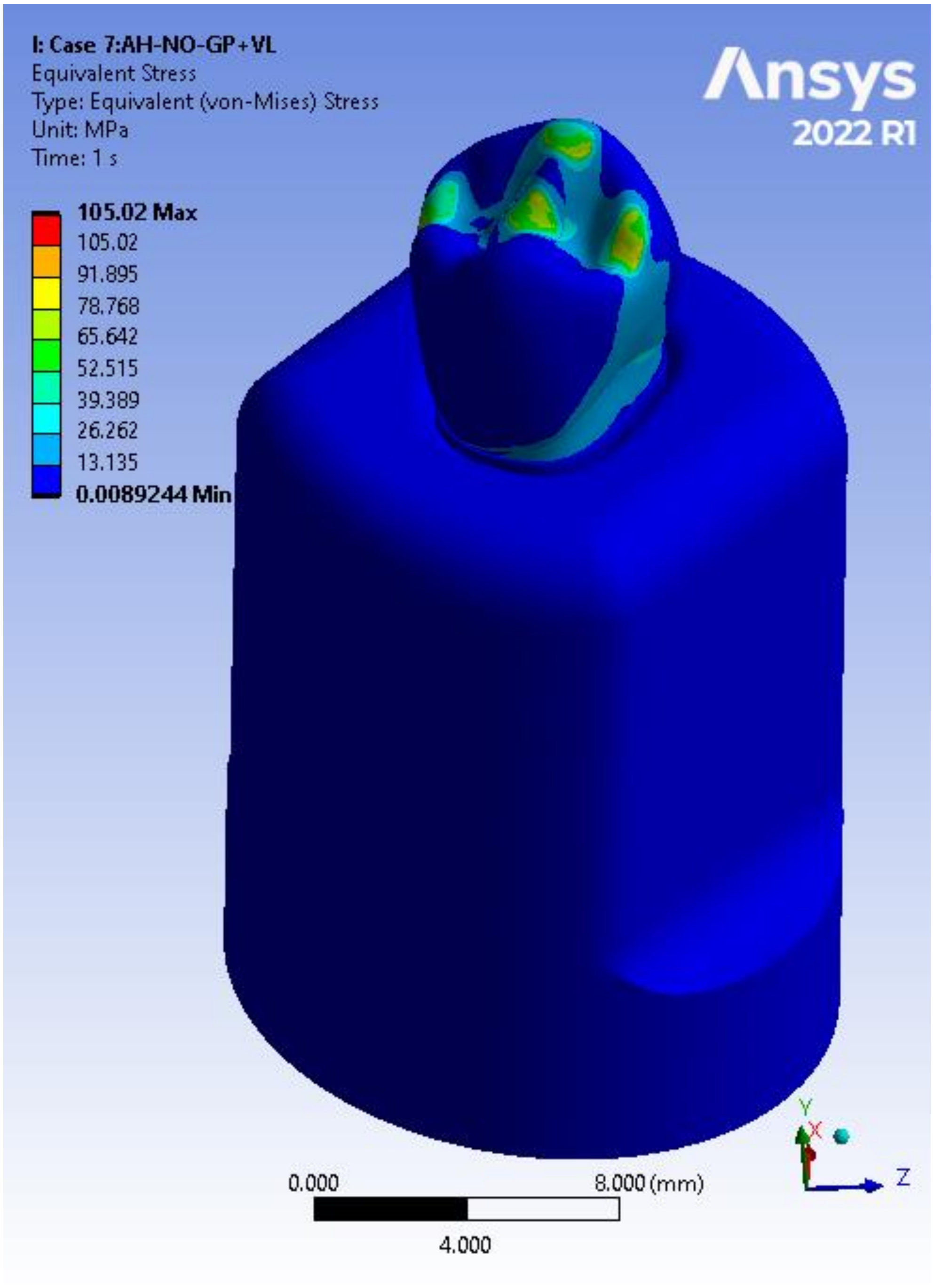
I: Case 7:AH-NO-GP+VL
Equivalent Stress
Type: Equivalent (von-Mises) Stress
Unit: MPa
Time: 1 s
105.02 Max
105.02
91.895
78.768
65.642
52.515
39.389
26.262
13.135
0.0089244 Min
Ansys
2022 R1
0.000
8.000 (mm)
4.000
Y
X
Z

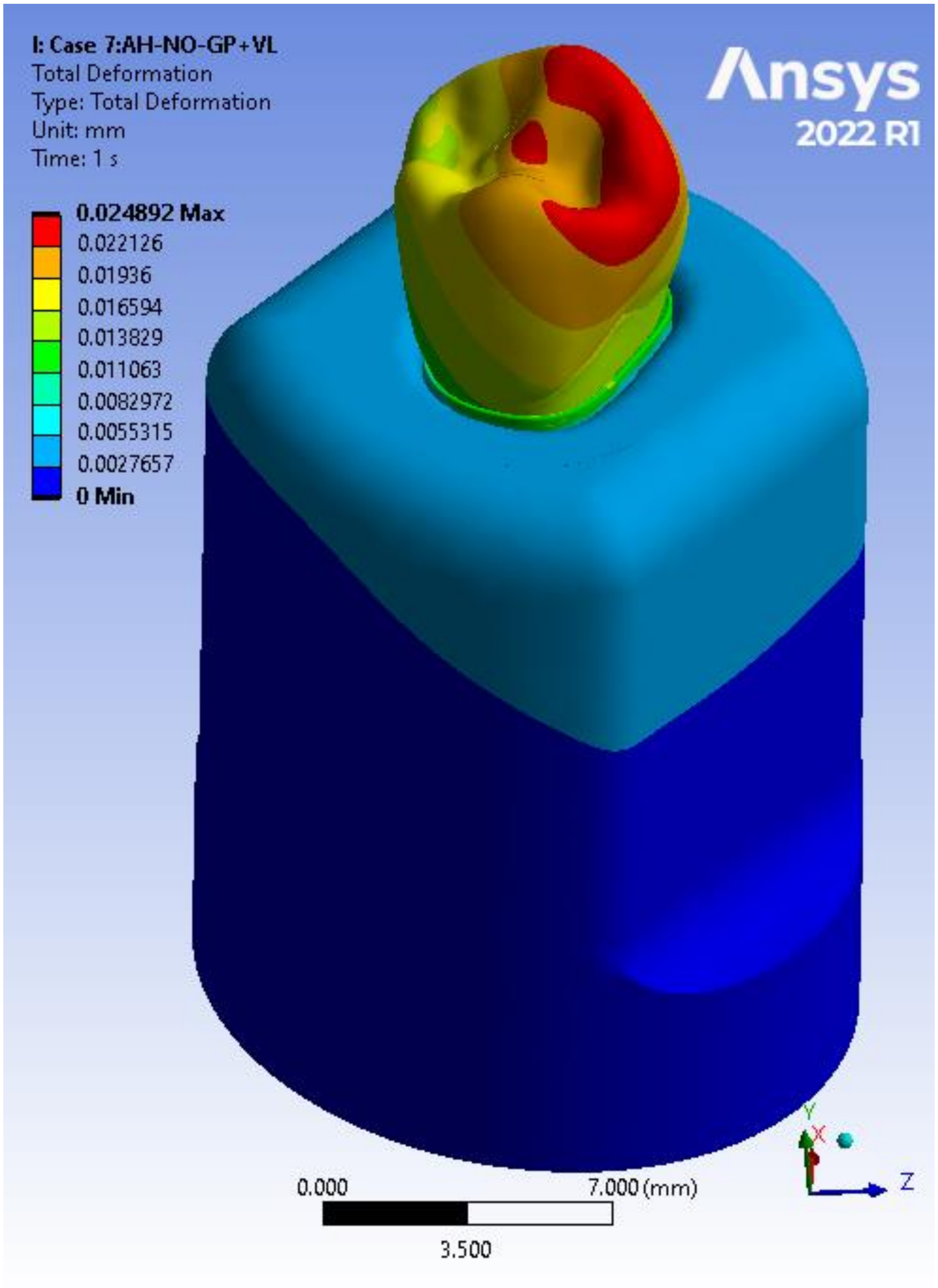
I: Case 7:AH-NO-GP+VL
Total Deformation
Type: Total Deformation
Unit: mm
Time: 1 s
0.024892 Max
0.022126
0.01936
0.016594
0.013829
0.011063
0.0082972
0.0055315
0.0027657
0 Min
Ansys
2022 R1
0.000
7.000 (mm)
3.500
Y
X
Z

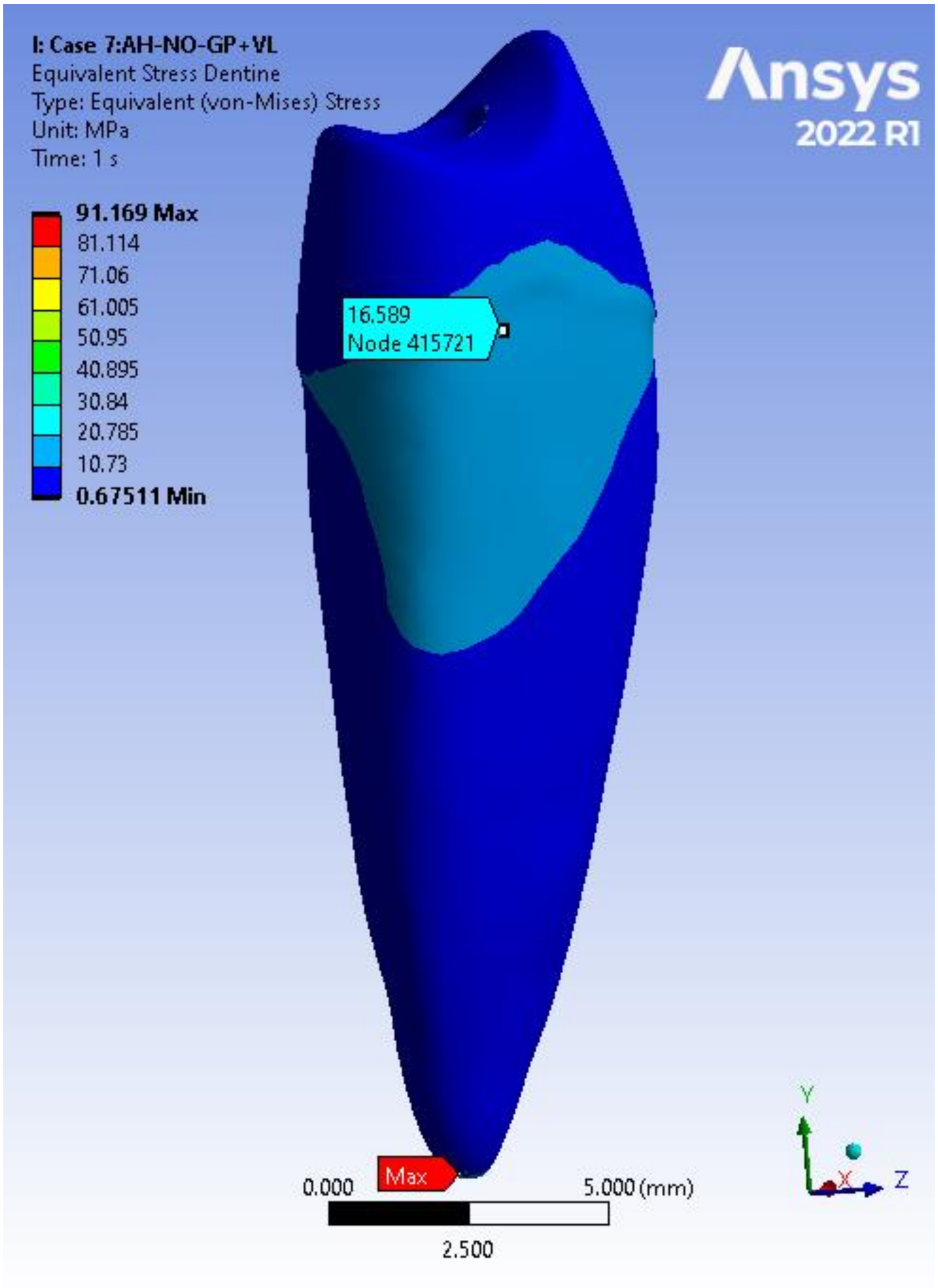
I: Case 7:AH-NO-GP+VL
Equivalent Stress Dentine
Type: Equivalent (von-Mises) Stress
Unit: MPa
Time: 1 s
91.169 Max
81.114
71.06
61.005
50.95
40.895
30.84
20.785
10.73
0.67511 Min
16.589
Node 415721
Ansys
2022 R1
Max
0.000
5.000 (mm)
2.500
Y
X
Z

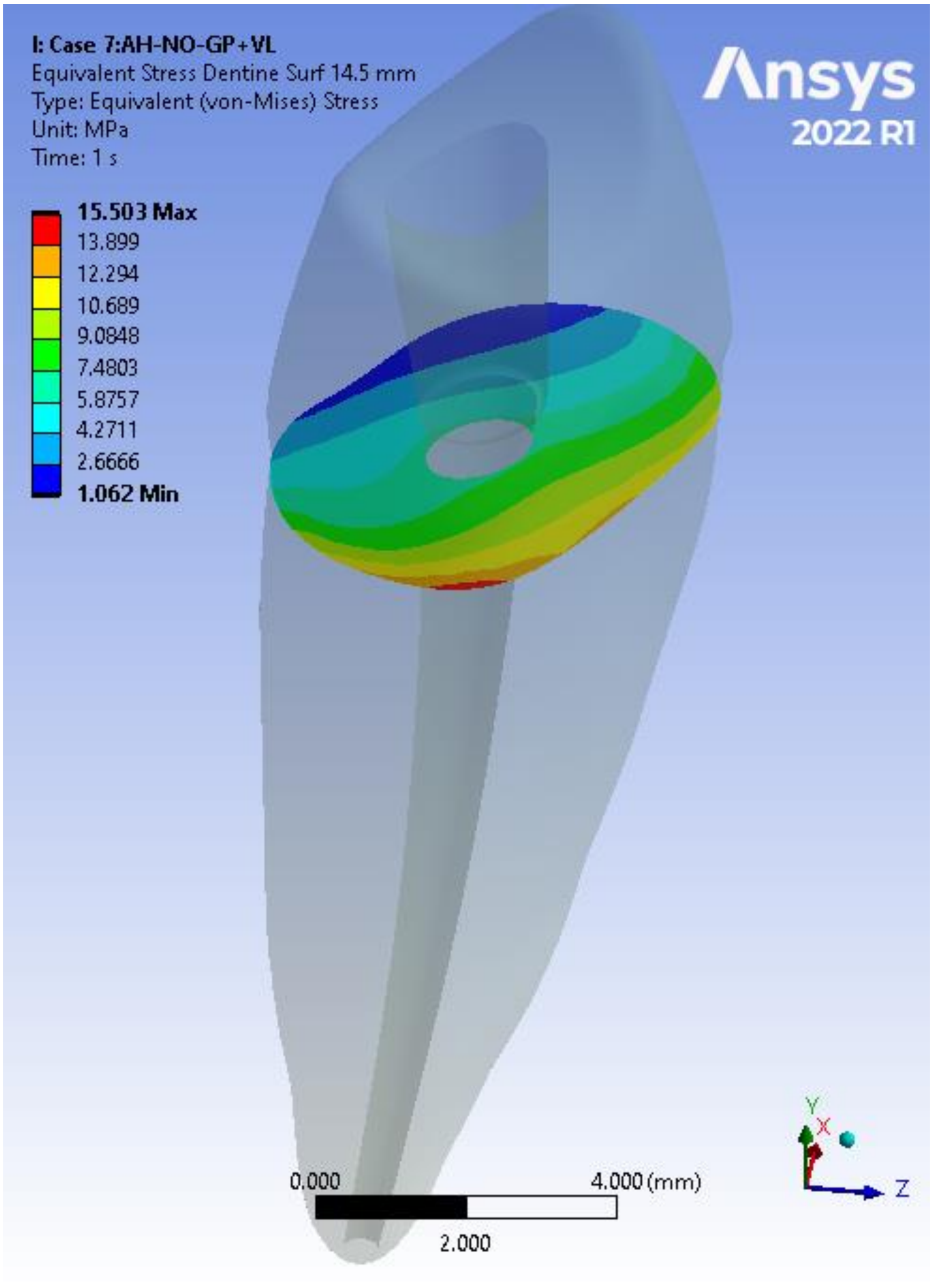

I: Case 7:AH-NO-GP+VL
Equivalent Stress Dentine Surf 14.5 mm
Type: Equivalent (von-Mises) Stress
Unit: MPa
Time: 1 s
15.503 Max
13.899
12.294
10.689
9.0848
7.4803
5.8757
4.2711
2.6666
1.062 Min
Ansys
2022 R1
0.000
4.000 (mm)
2.000
Y
X
Z

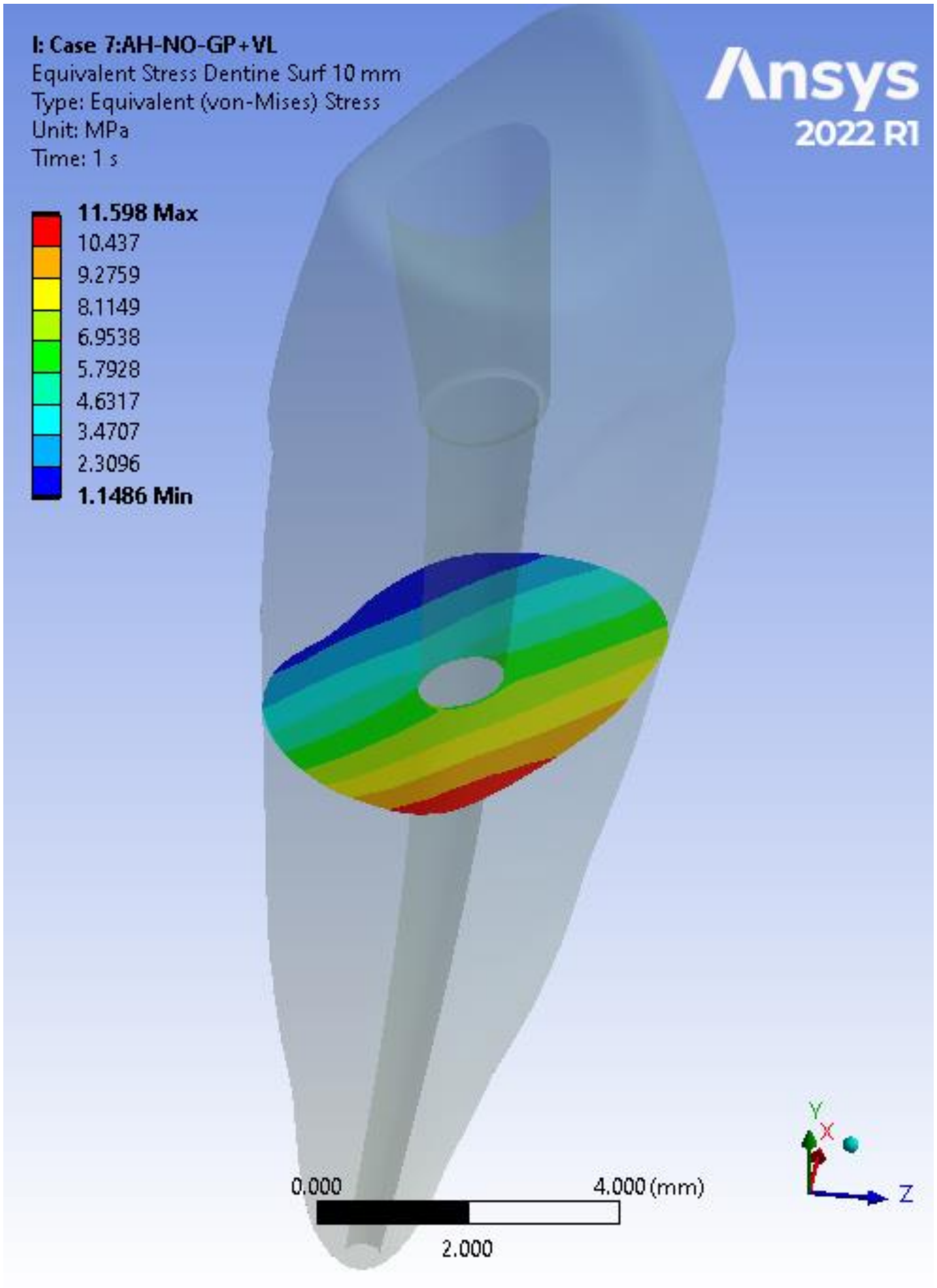
I: Case 7:AH-NO-GP+VL
Equivalent Stress Dentine Surf 10 mm
Type: Equivalent (von-Mises) Stress
Unit: MPa
Time: 1 s
11.598 Max
10.437
9.2759
8.1149
6.9538
5.7928
4.6317
3.4707
2.3096
1.1486 Min
Ansys
2022 R1
0.000
4.000 (mm)
2.000
Y
X
Z

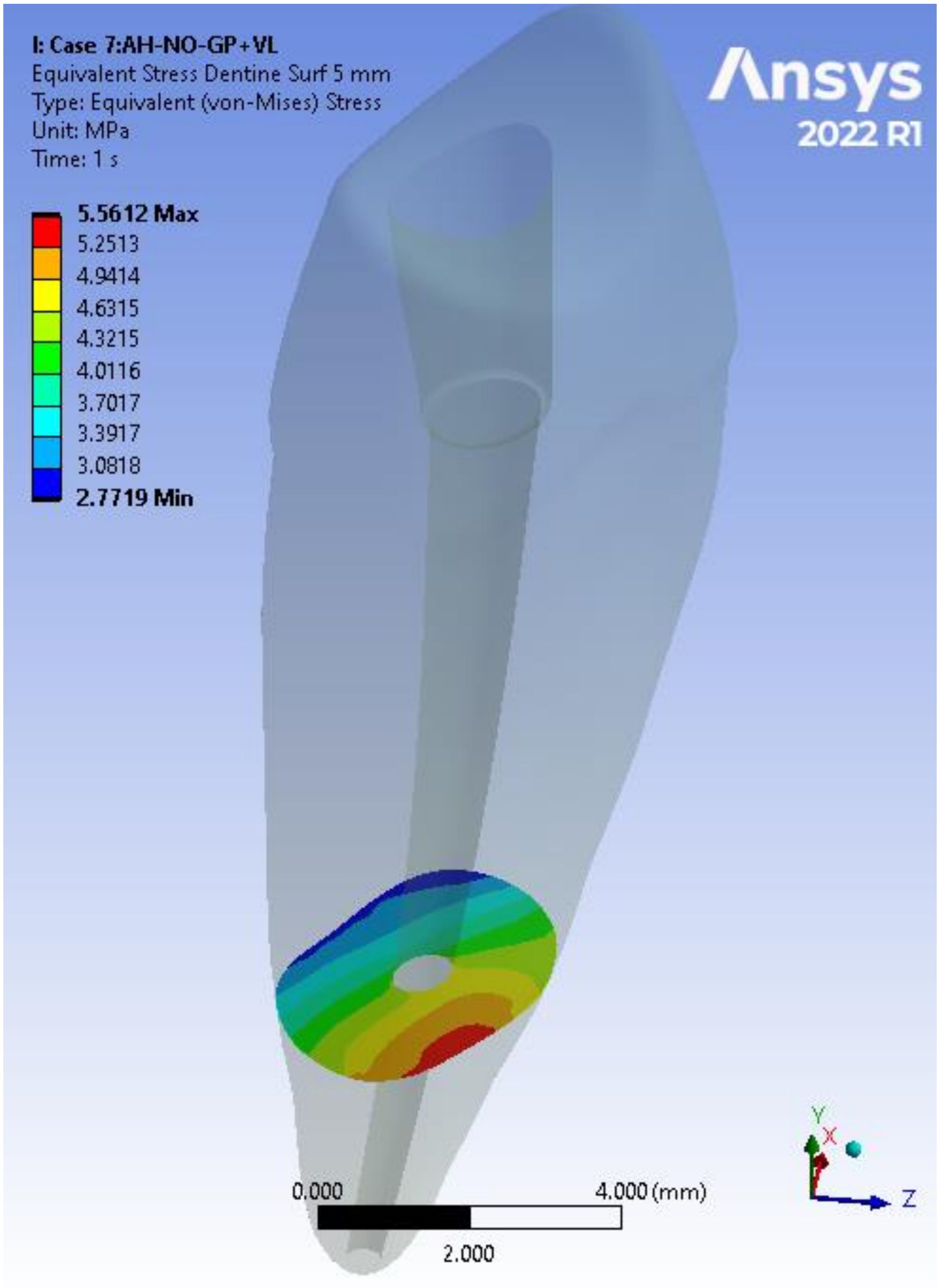

I: Case 7:AH-NO-GP+VL
Equivalent Stress Dentine Surf 5 mm
Type: Equivalent (von-Mises) Stress
Unit: MPa
Time: 1 s
5.5612 Max
5.2513
4.9414
4.6315
4.3215
4.0116
3.7017
3.3917
3.0818
2.7719 Min
Ansys
2022 R1
0.000
4.000 (mm)
2.000
Y
X
Z

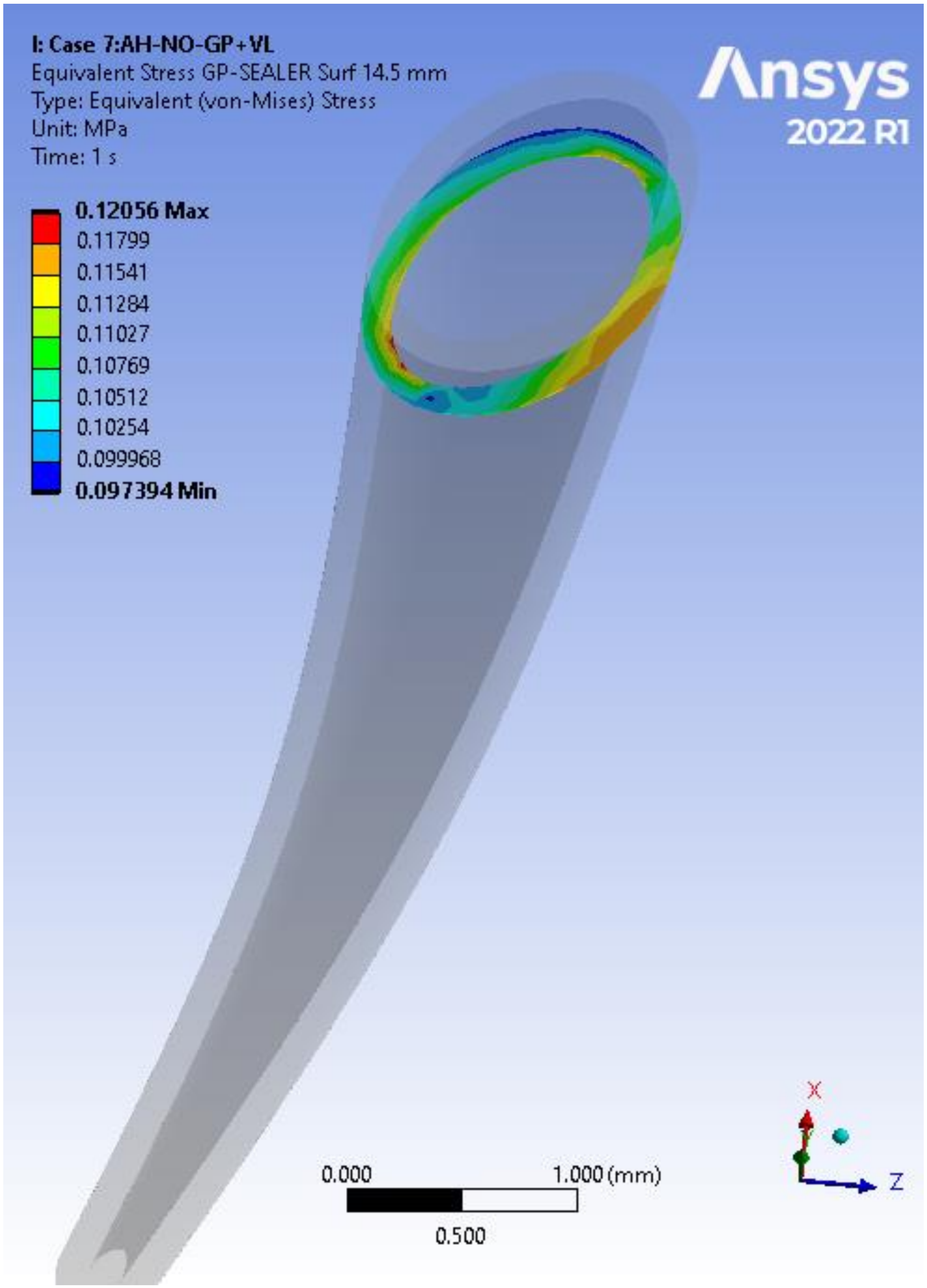
I: Case 7:AH-NO-GP+VL
Equivalent Stress GP-SEALER Surf 14.5 mm
Type: Equivalent (von-Mises) Stress
Unit: MPa
Time: 1 s
0.12056 Max
0.11799
0.11541
0.11284
0.11027
0.10769
0.10512
0.10254
0.099968
0.097394 Min
Ansys
2022 R1
0.000
1.000 (mm)
0.500
X
Z

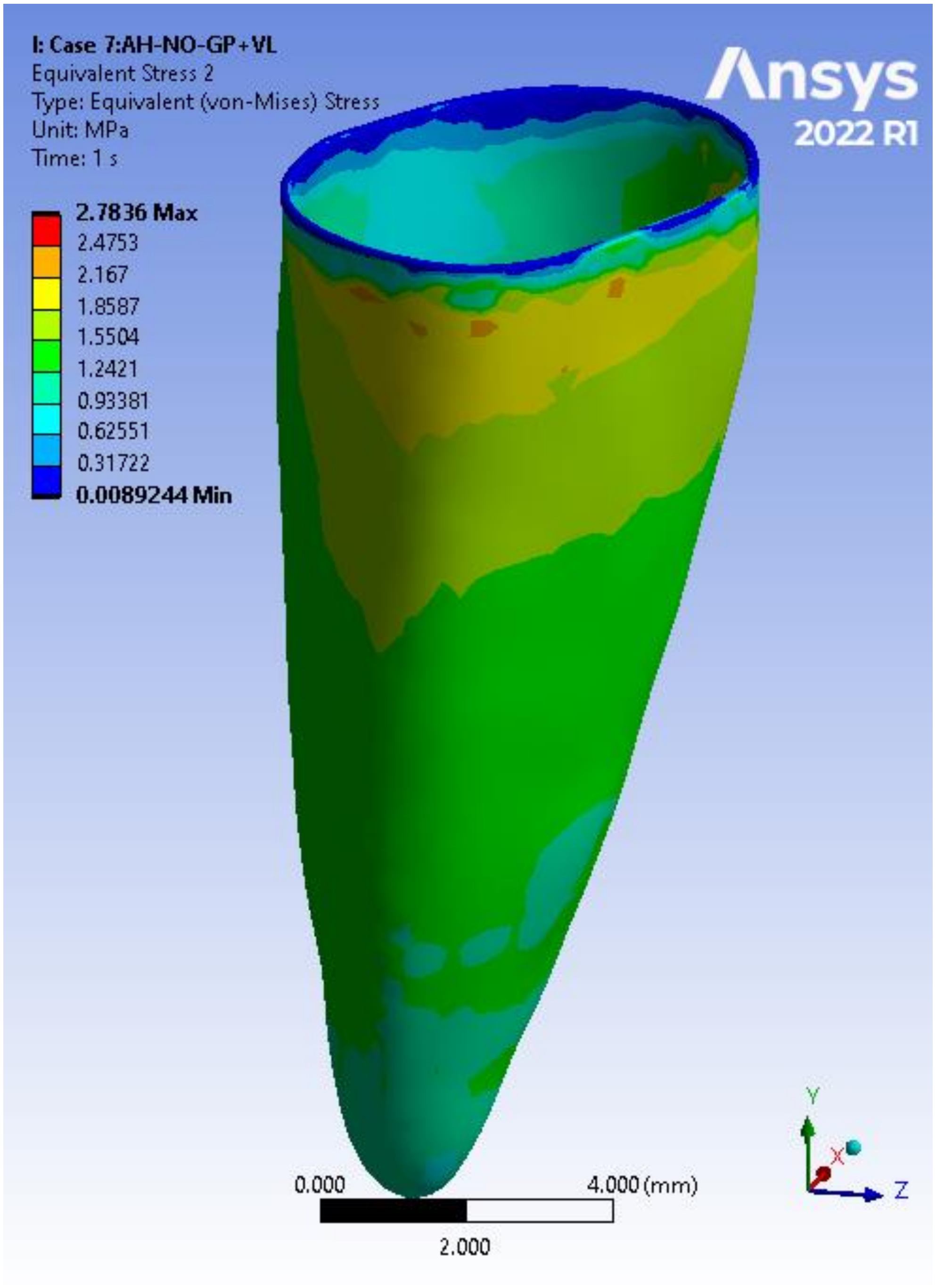
I: Case 7:AH-NO-GP+VL
Equivalent Stress 2
Type: Equivalent (von-Mises) Stress
Unit: MPa
Time: 1 s
2.7836 Max
2.4753
2.167
1.8587
1.5504
1.2421
0.93381
0.62551
0.31722
0.0089244 Min
Ansys
2022 R1
0.000
4.000 (mm)
2.000
Y
X
Z

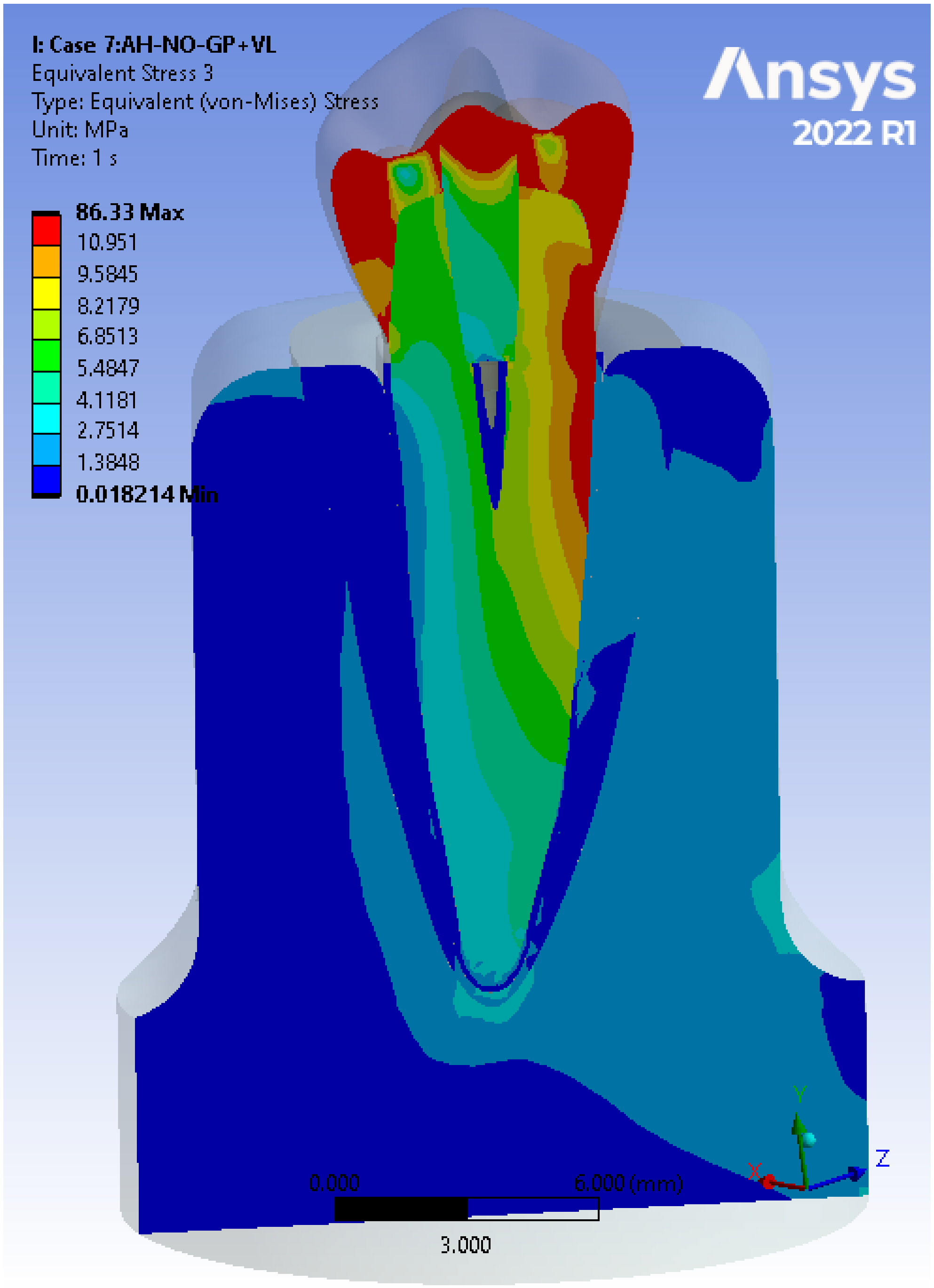
I: Case 7:AH-NO-GP+VL
Equivalent Stress 3
Type: Equivalent (von-Mises) Stress
Unit: MPa
Time: 1 s
86.33 Max
10.951
9.5845
8.2179
6.8513
5.4847
4.1181
2.7514
1.3848
0.018214 Min
Ansys
2022 R1
0.000
6.000 (mm)
3.000
Y
X
Z

Case 8

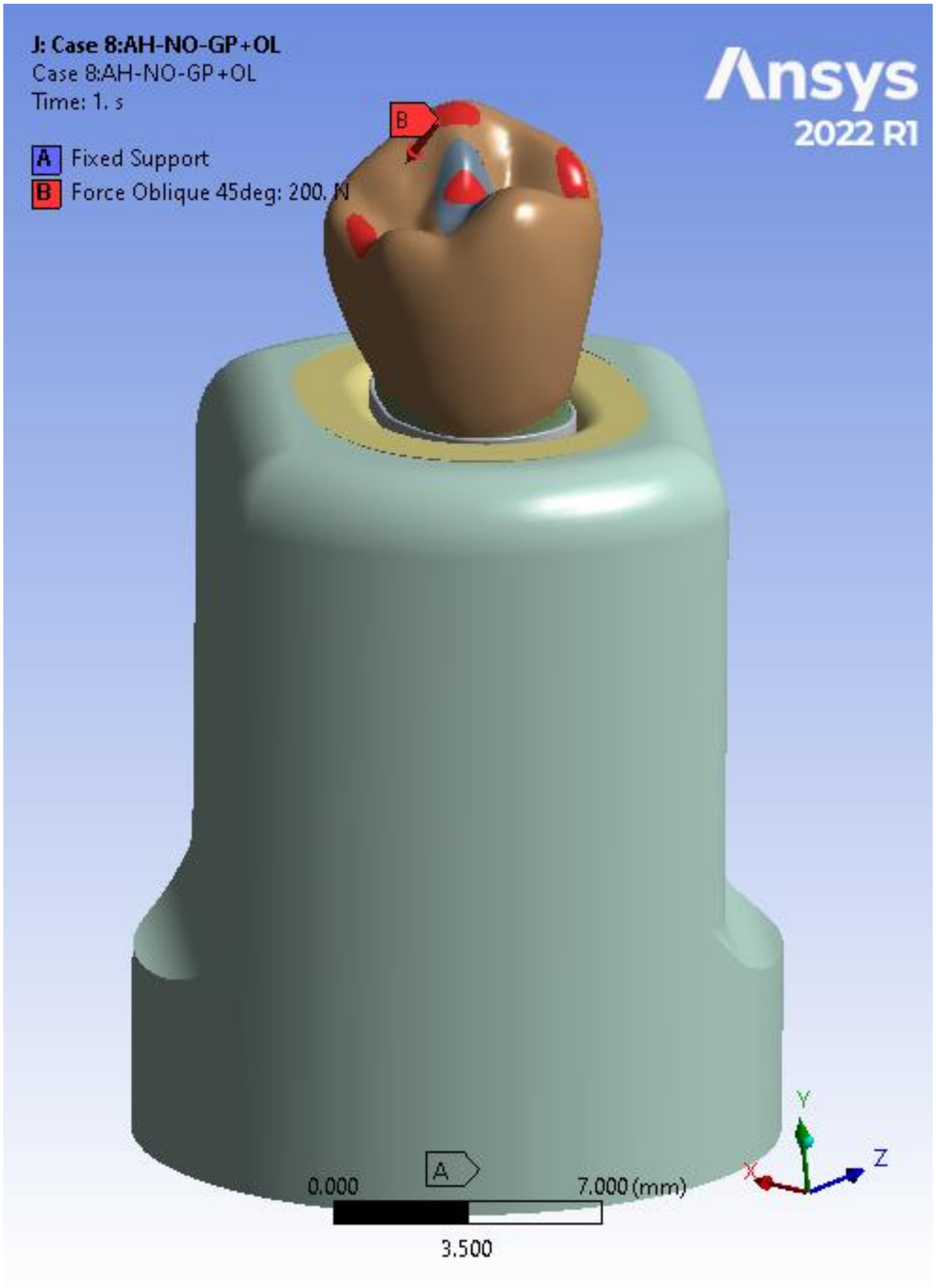
J: Case 8:AH-NO-GP+OL
Case 8:AH-NO-GP+OL
Time: 1. s
A Fixed Support
B Force Oblique 45deg: 200. N
Ansys
2022 R1
B
A
0.000
7.000 (mm)
3.500
Y
Z
X

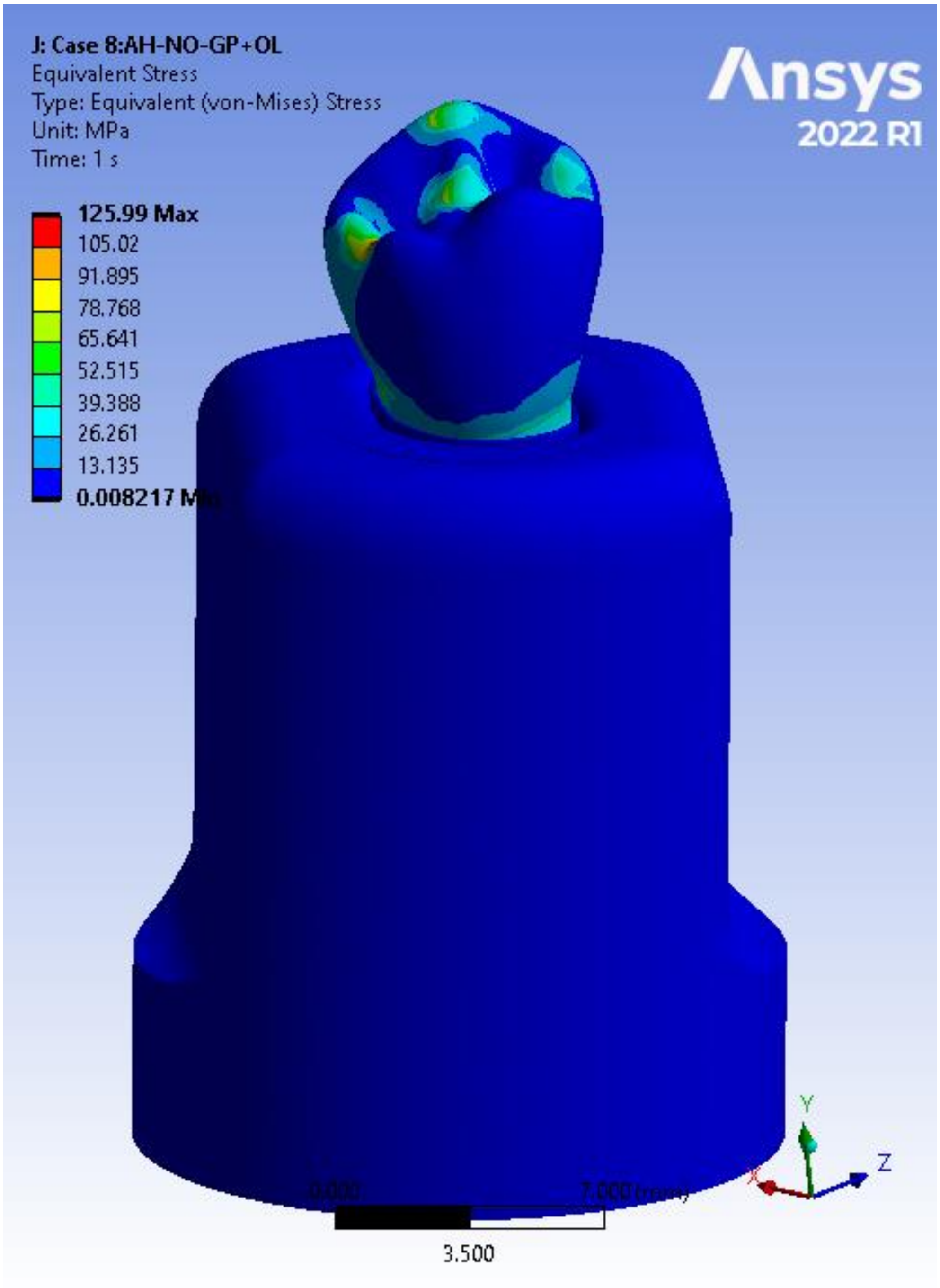

J: Case 8:AH-NO-GP+OL
Equivalent Stress
Type: Equivalent (von-Mises) Stress
Unit: MPa
Time: 1 s
125.99 Max
105.02
91.895
78.768
65.641
52.515
39.388
26.261
13.135
0.008217 Min
Ansys
2022 R1
0.000
7.000 (mm)
3.500
Y
Z
X

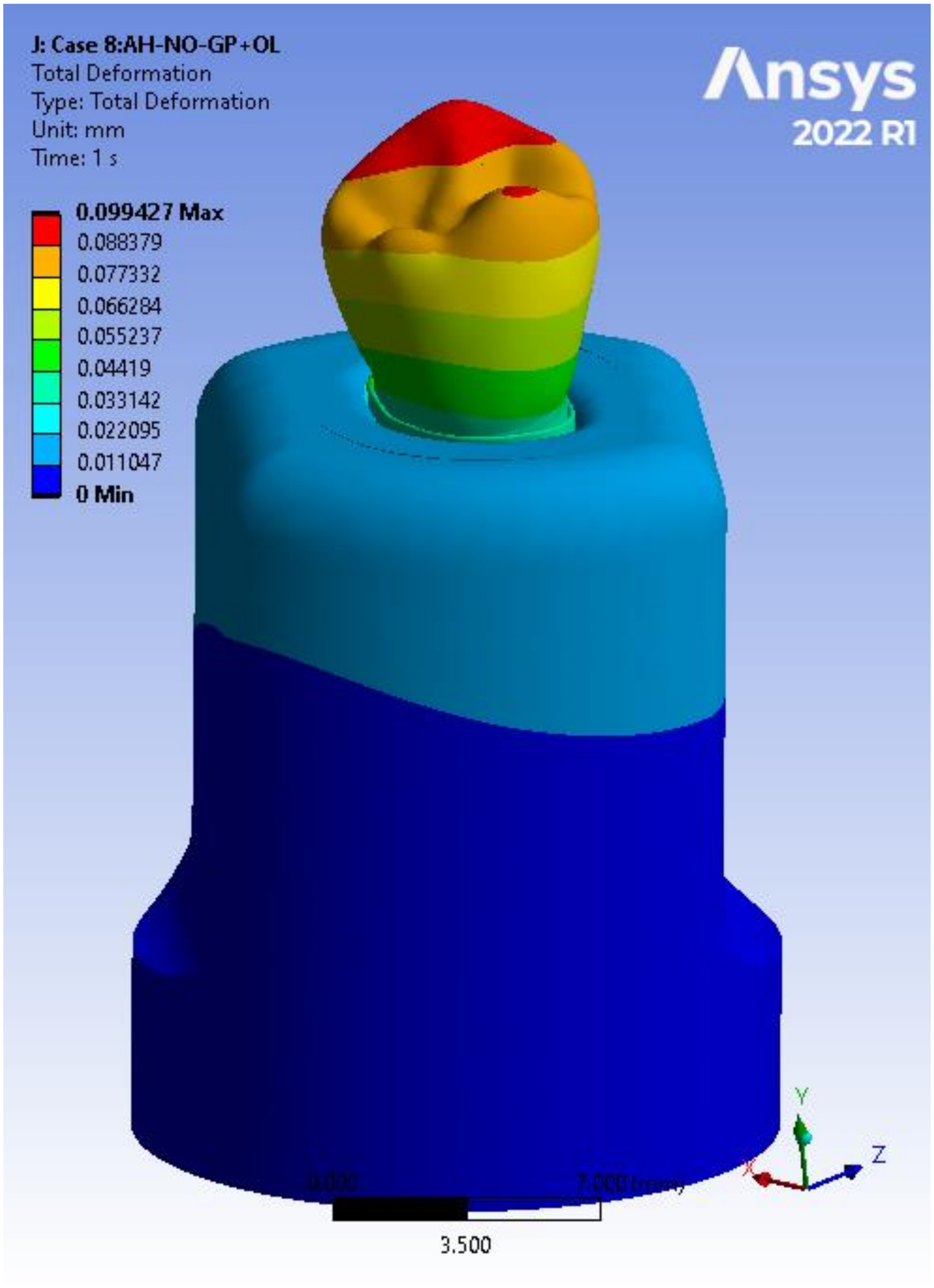
J: Case 8:AH-NO-GP+OL
Total Deformation
Type: Total Deformation
Unit: mm
Time: 1 s
0.099427 Max
0.088379
0.077332
0.066284
0.055237
0.04419
0.033142
0.022095
0.011047
0 Min
Ansys
2022 R1
0.000
7.000 (mm)
3.500
Y
Z
X

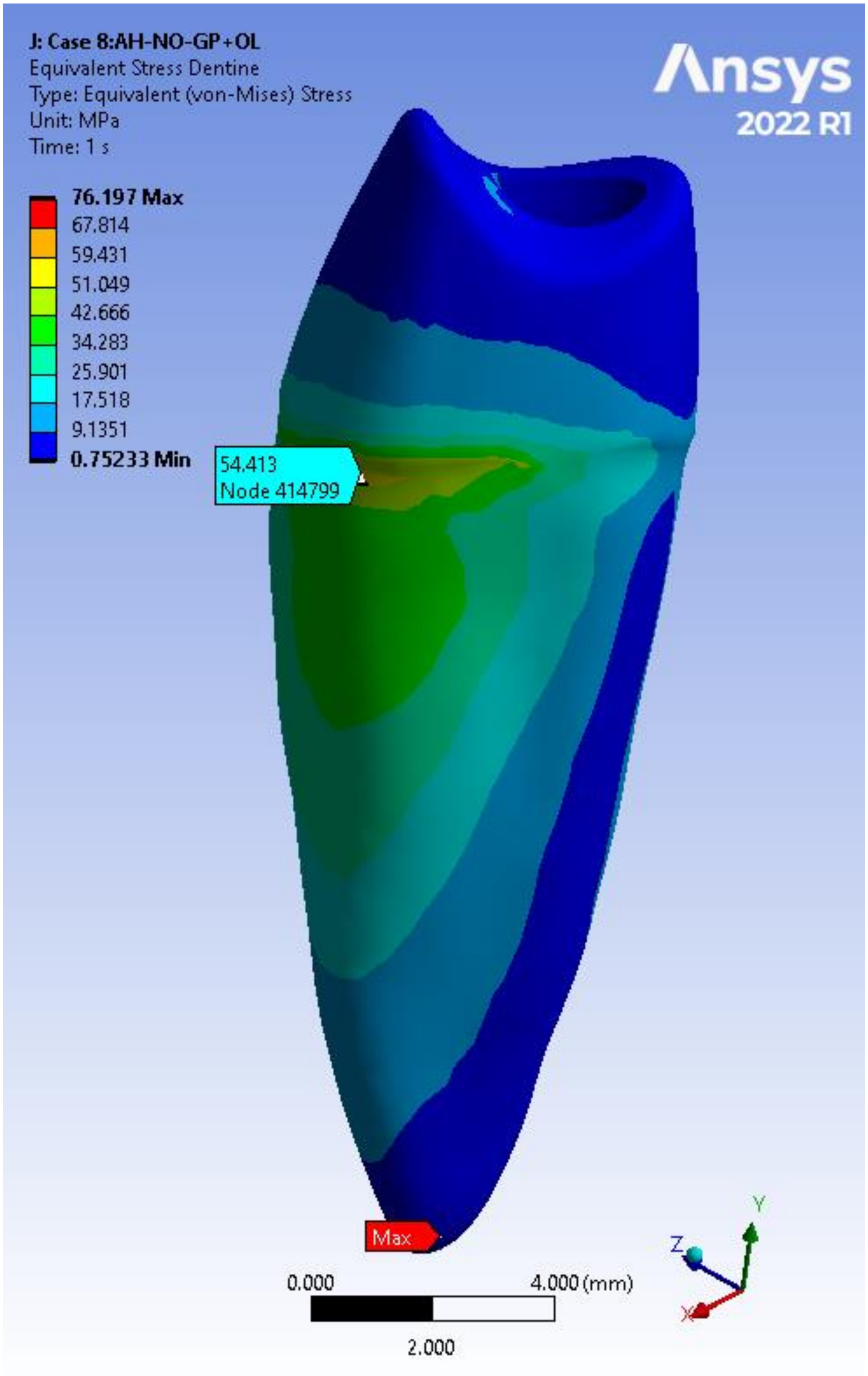
J: Case 8:AH-NO-GP+OL
Equivalent Stress Dentine
Type: Equivalent (von-Mises) Stress
Unit: MPa
Time: 1 s
Ansys
2022 R1
76.197 Max
67.814
59.431
51.049
42.666
34.283
25.901
17.518
9.1351
0.75233 Min
54.413
Node 414799
Max
0.000
4.000 (mm)
2.000
Y
Z
X

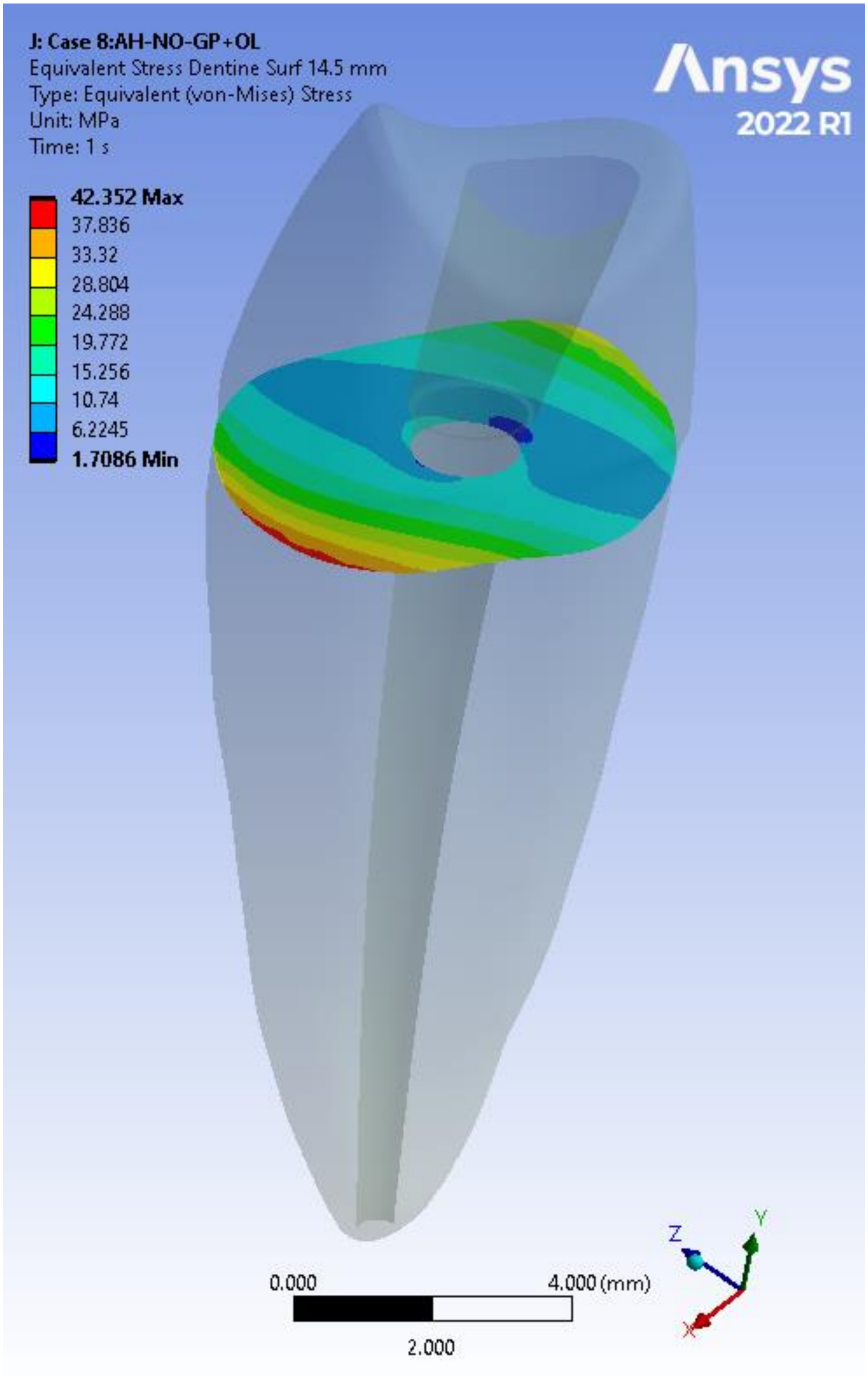

J: Case 8:AH-NO-GP+OL
Equivalent Stress Dentine Surf 14.5 mm
Type: Equivalent (von-Mises) Stress
Unit: MPa
Time: 1 s
42.352 Max
37.836
33.32
28.804
24.288
19.772
15.256
10.74
6.2245
1.7086 Min
Ansys
2022 R1
0.000
4.000 (mm)
2.000
Z
Y
X

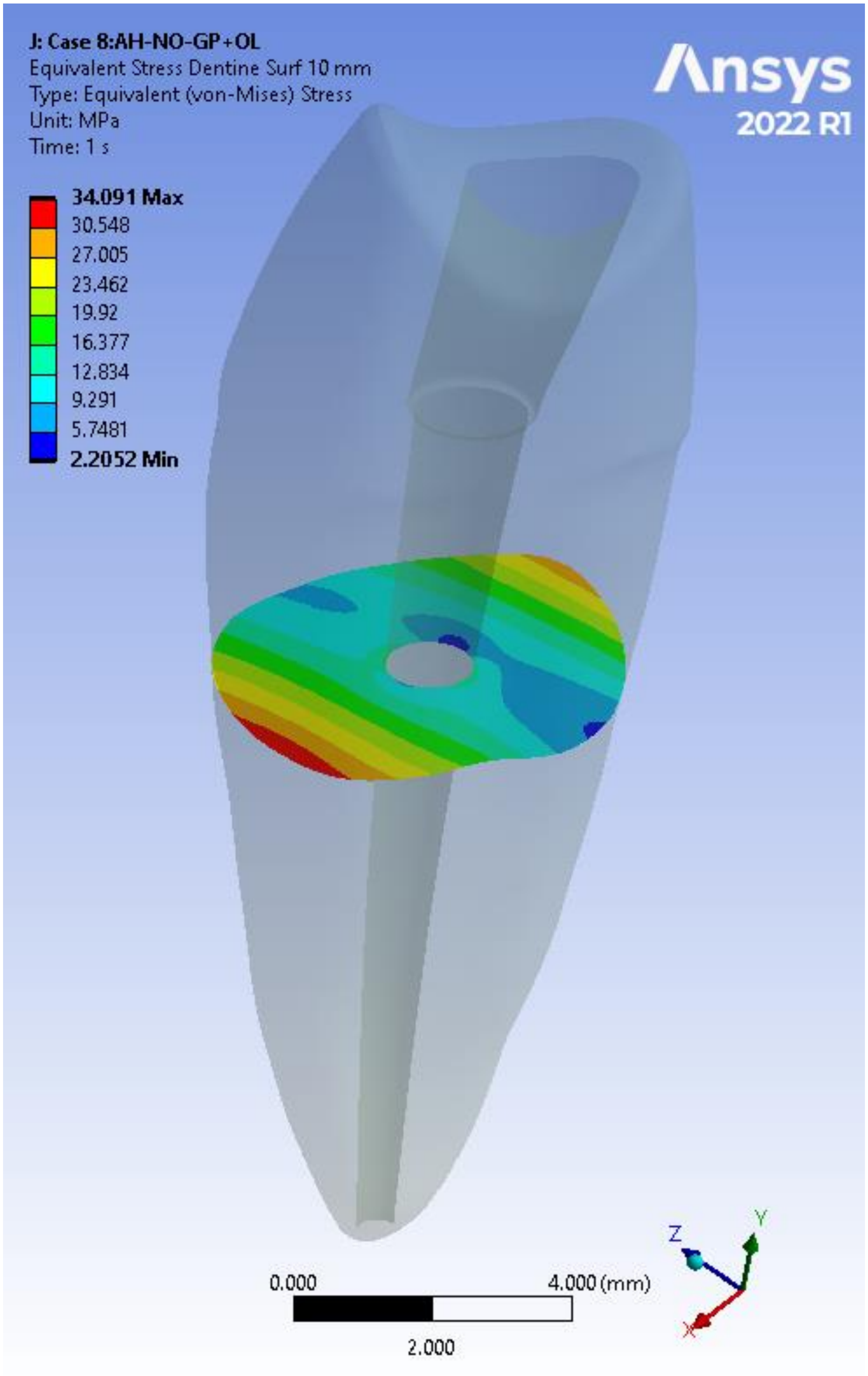
J: Case 8:AH-NO-GP+OL
Equivalent Stress Dentine Surf 10 mm
Type: Equivalent (von-Mises) Stress
Unit: MPa
Time: 1 s
34.091 Max
30.548
27.005
23.462
19.92
16.377
12.834
9.291
5.7481
2.2052 Min
Ansys
2022 R1
0.000
4.000 (mm)
2.000
Z
Y
X

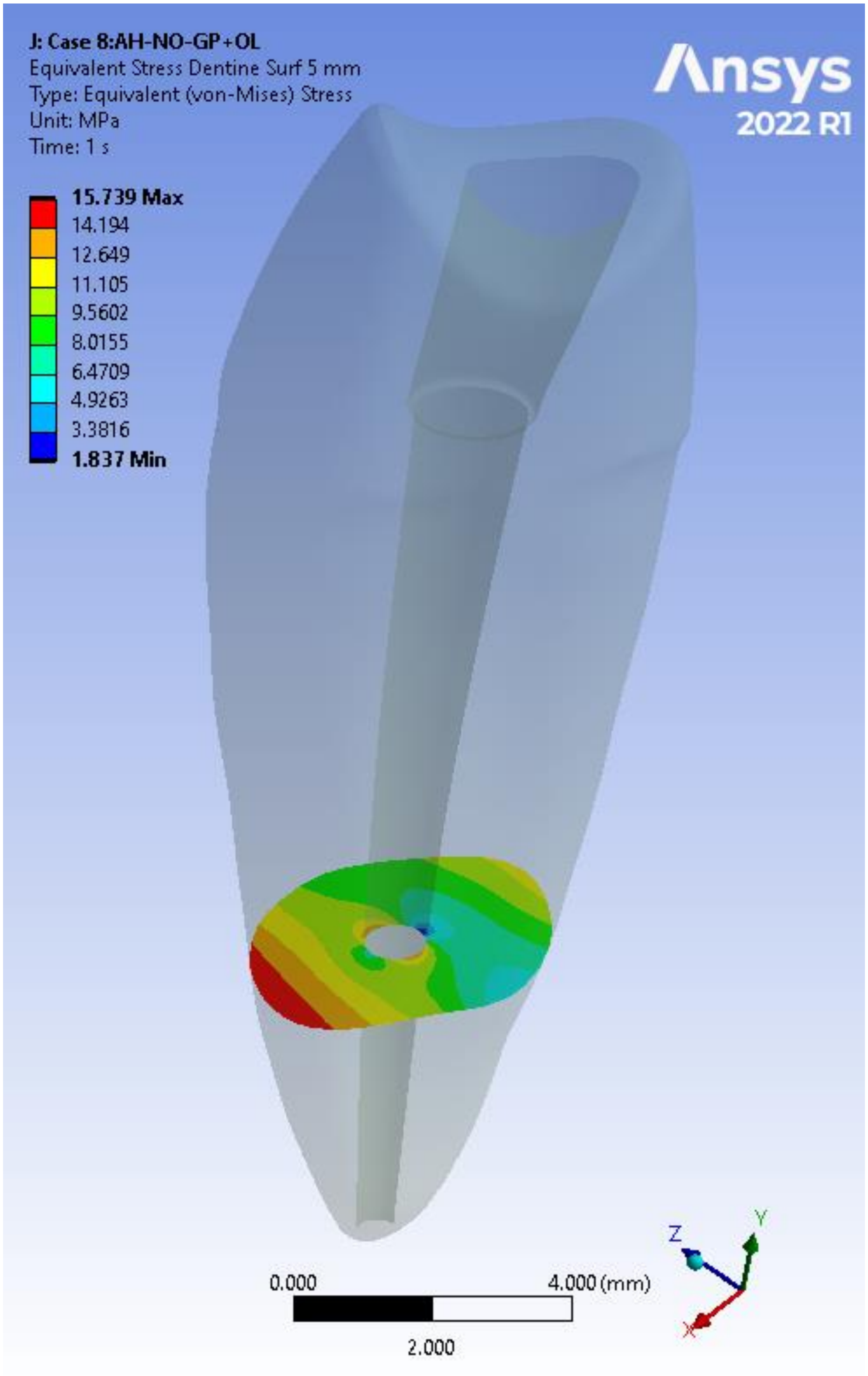
J: Case 8:AH-NO-GP+OL
Equivalent Stress Dentine Surf 5 mm
Type: Equivalent (von-Mises) Stress
Unit: MPa
Time: 1 s
15.739 Max
14.194
12.649
11.105
9.5602
8.0155
6.4709
4.9263
3.3816
1.837 Min
Ansys
2022 R1
0.000
4.000 (mm)
2.000
Z
Y
X

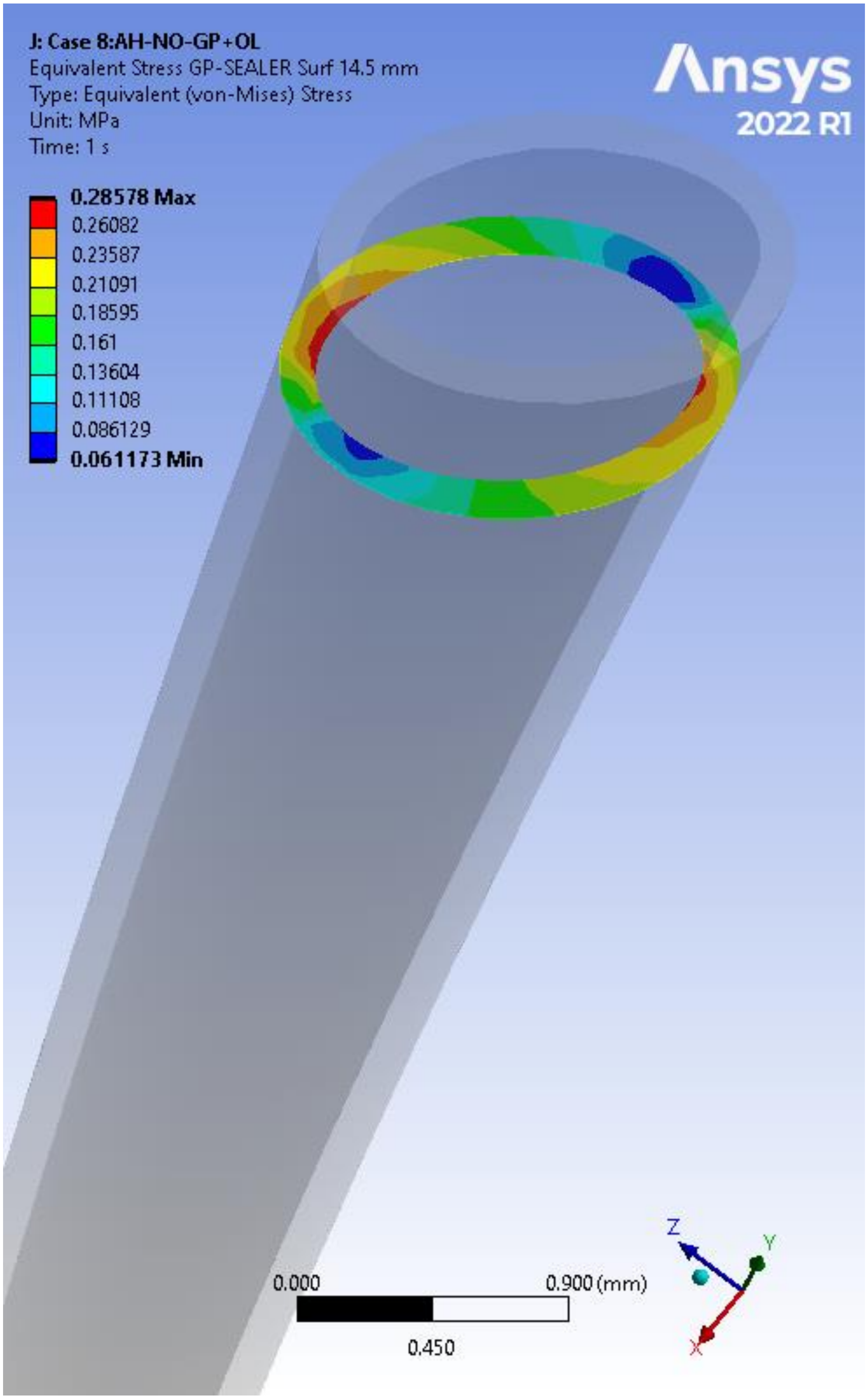
J: Case 8:AH-NO-GP+OL
Equivalent Stress GP-SEALER Surf 14.5 mm
Type: Equivalent (von-Mises) Stress
Unit: MPa
Time: 1 s
0.28578 Max
0.26082
0.23587
0.21091
0.18595
0.161
0.13604
0.11108
0.086129
0.061173 Min
Ansys
2022 R1
0.000
0.900 (mm)
0.450
Z
Y
X

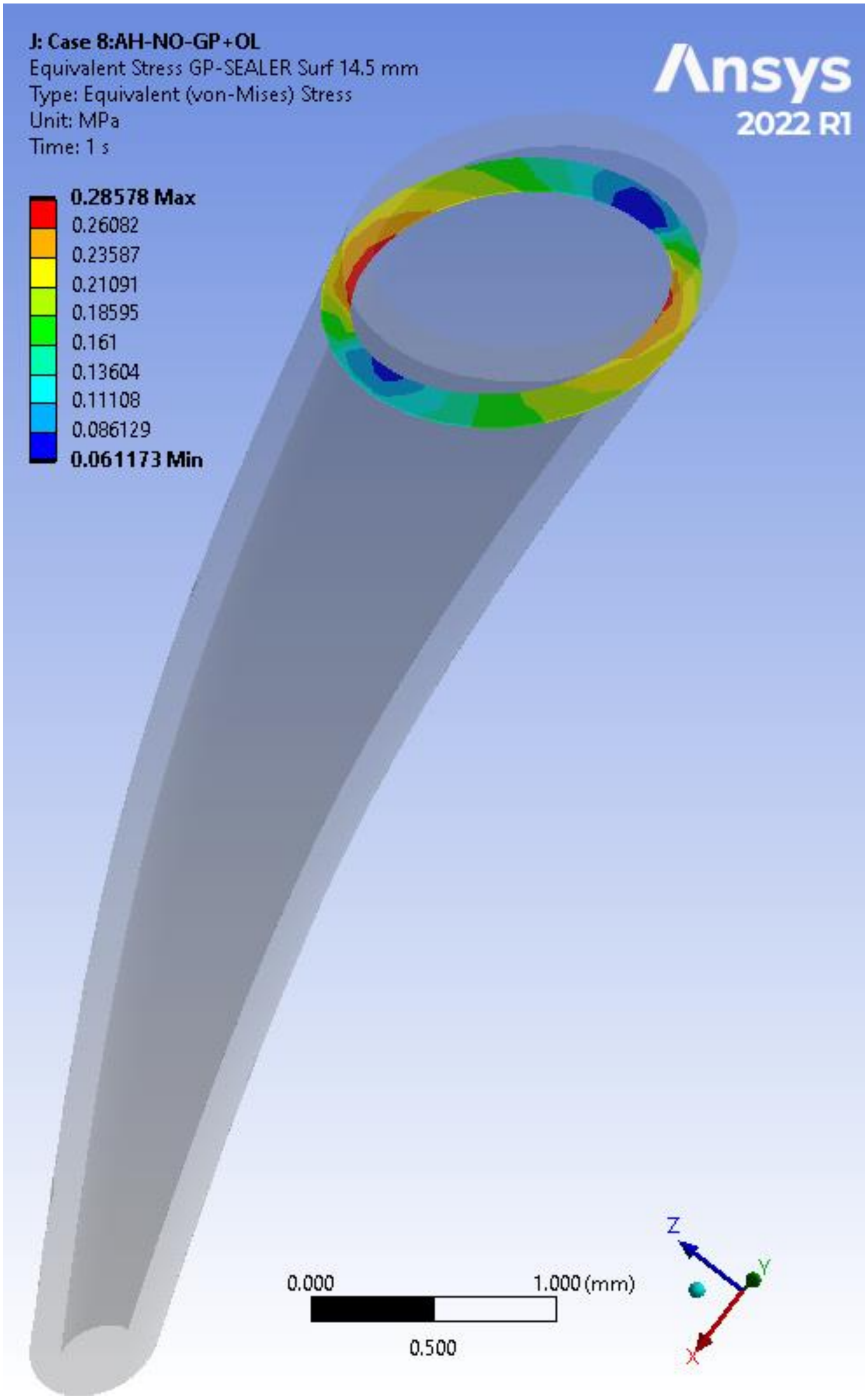
J: Case 8:AH-NO-GP+OL
Equivalent Stress GP-SEALER Surf 14.5 mm
Type: Equivalent (von-Mises) Stress
Unit: MPa
Time: 1 s
0.28578 Max
0.26082
0.23587
0.21091
0.18595
0.161
0.13604
0.11108
0.086129
0.061173 Min
Ansys
2022 R1
0.000
1.000 (mm)
0.500
Z
Y
X

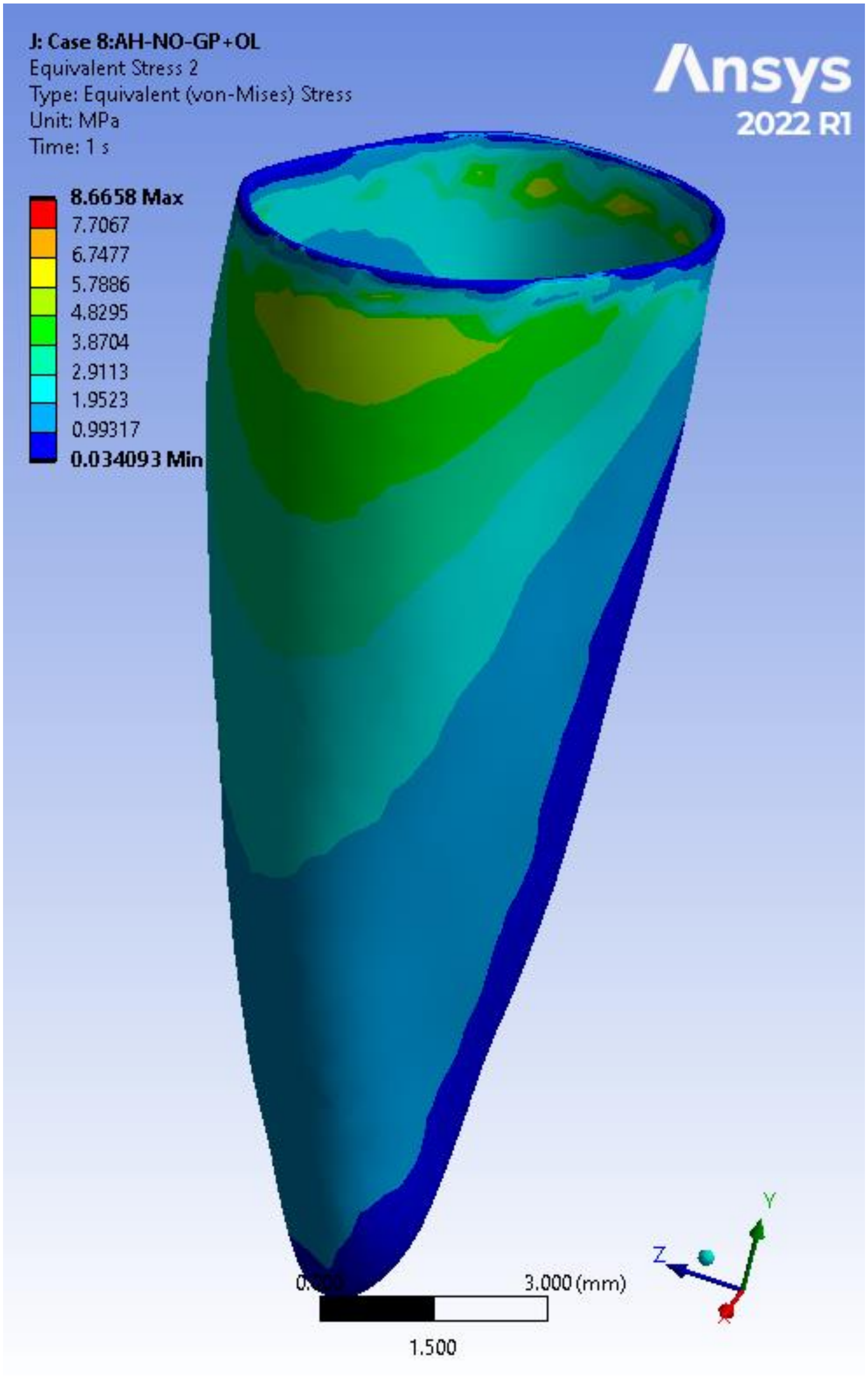
J: Case 8:AH-NO-GP+OL
Equivalent Stress 2
Type: Equivalent (von-Mises) Stress
Unit: MPa
Time: 1 s
8.6658 Max
7.7067
6.7477
5.7886
4.8295
3.8704
2.9113
1.9523
0.99317
0.034093 Min
Ansys
2022 R1
3.000 (mm)
1.500
Y
Z
X

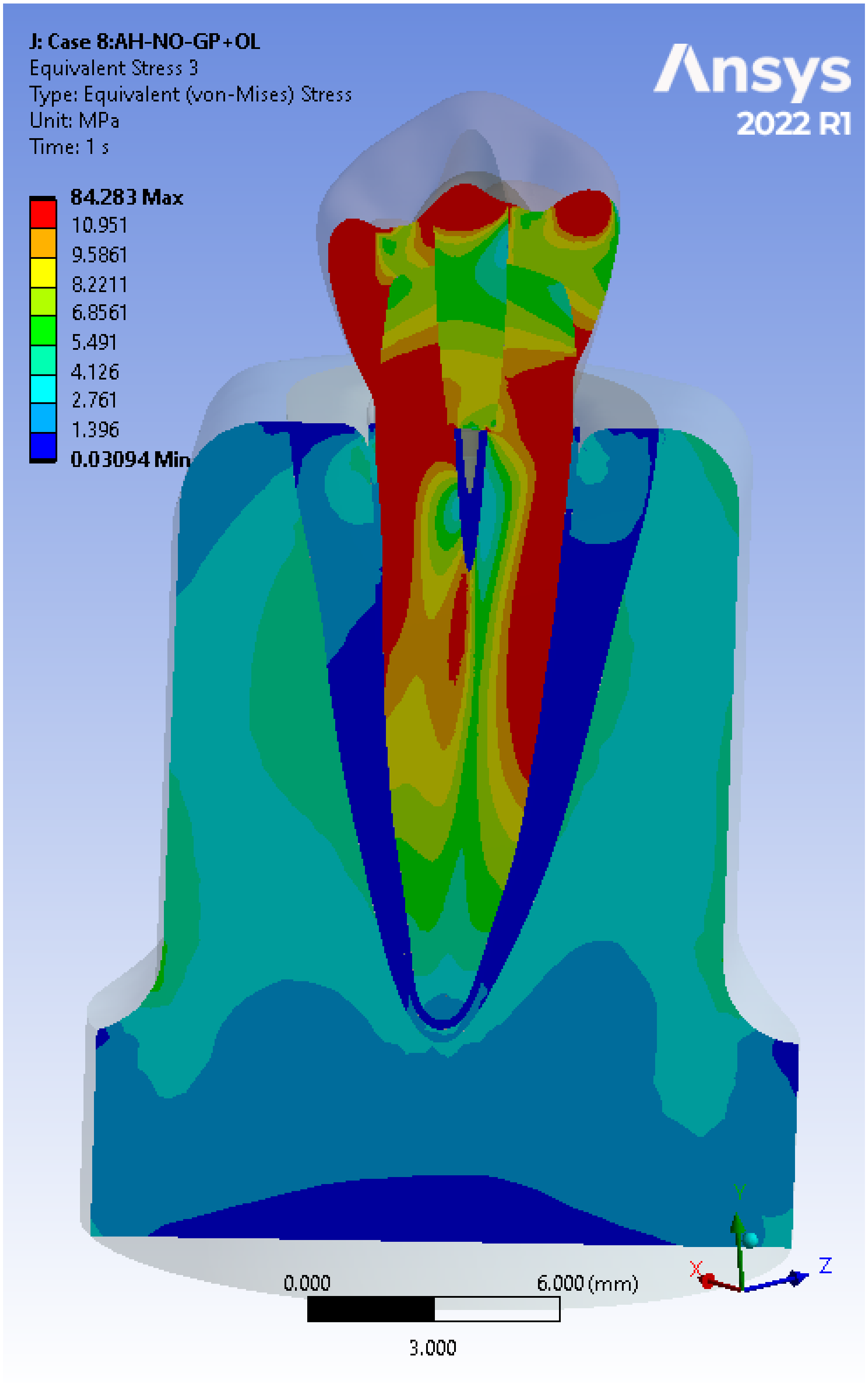

J: Case 8:AH-NO-GP+OL
Equivalent Stress 3
Type: Equivalent (von-Mises) Stress
Unit: MPa
Time: 1 s
84.283 Max
10.951
9.5861
8.2211
6.8561
5.491
4.126
2.761
1.396
0.03094 Min
Ansys
2022 R1
0.000
6.000 (mm)
3.000
Y
X
Z
